# Protein profiles of bacteriophages of the family *Myoviridae*-like induced on *M. haemolytica*

**DOI:** 10.1186/s13568-018-0630-3

**Published:** 2018-06-19

**Authors:** Renata Urban-Chmiel, Andrzej Wernicki, Jacek Wawrzykowski, Andrzej Puchalski, Anna Nowaczek, Marta Dec, Diana Stęgierska, Mohammed Mijbas Mohammed Alomari

**Affiliations:** 1 Sub-department of Veterinary Prevention and Avian Diseases, Institute of Biological Bases of Animal Diseases, Faculty of Veterinary Medicine, University of Life Sciences, 20-033 Lublin, Poland; 2 Department of Biochemistry, Faculty Veterinary Medicine, University of Life Sciences, 20-033 Lublin, Poland; 3 Faculty of Veterinary Medicine, University of Al Muthanna, Samawah, Iraq

**Keywords:** Bacteriophages, *M. haemolytica*, SDS-PAGE, Two-dimensional electrophoresis, Mass spectrometry

## Abstract

The aim of study was to isolate, characterize and analyse the protein profiles of *Myoviridae*-like bacteriophages obtained from *M. haemolytica* using MALDI TOF mass spectrometry. The material consisted of the *M. haemolytica* reference strain ATCC^®^ BAA410, reference serotypes A1, A2, A5, A6, A7, A9, and A11, and wild-type isolates of serotype A1. Bacteriophage morphology was examined with a transmission electron microscope. The proteins were separated in SDS-PAGE and two-dimensional electrophoresis and characterized by MALDI-TOF. Among the phages obtained, seven were specific for strains A1, A2, A5, A6, A7 and 25, and PHL-1 was specific for the BAA410 strain. The protein profiles for the phages were very similar to one another, but differed from the reference phage in that they lacked protein fractions with molecular weights of 22.9, 56.3 and 73.1 kDa. 2D electrophoresis revealed significant differences in the size of proteins and their localization in the pH gradient. The most similar profiles were observed in phages specific for strains BAA-410 and A6. In all profiles two main spots were observed in the molecular weight range from 44 to 70 kDa at pH < 4. The results indicate that 2D electrophoresis is a very useful tool for characterization of phage protein profiles. An important objective was to determine the molecular differences between morphologically similar phages belonging to one family and to find similarities to phages specific for other pathogens. The study also assessed the suitability of the methods used to characterize phages.

**Electronic supplementary material:**

The online version of this article (10.1186/s13568-018-0630-3) contains supplementary material, which is available to authorized users.

## Introduction

All types of bacteria exhibit susceptibility to bacteriophages specific to them. Numerous studies have shown that bacteriophages can be isolated from a variety of environments in which bacteria are present. Examples include natural water bodies, wastewater, soil, plants and animals (Kutateladze and Adamia 2010; Leverentz et al. 2004).

Bacteriophages, apart from genetic material consisting of nucleic acid (RNA or DNA), are composed of a protein coat forming the structure of the phage. Traditional methods for identifying bacteriophages make it possible to determine the family they belong to on the basis of their morphological structure, as well as their range of lytic activity. Restriction analysis of the genetic material of phages can be used to classify them to species and evaluate relatedness within the species, family and group. In some cases (Zhang et al. 2006), comprehensive restriction analysis is not possible because identical restriction profiles are obtained for phages belonging to the same or different families. Numerous studies (Bovet et al. 1990; Ngwai et al. 2005) have confirmed that detailed analysis of protein profiles may be a useful element in differentiating even bacteriophages belonging to the same family.

Molecular methods such as polyacrylamide gel electrophoresis (SDS PAGE) are able to determine the molecular weights of phage proteins, enabling detection of individual protein molecules during identification of viruses and bacteria isolated from different environments (Zimmer et al. 2002). The use of SDS-PAGE in many studies has enabled detection and detailed analysis of the protein profiles of phages specific for *E. coli* and *S. aureus* (Ngwai et al. 2005; Elshayeb et al. 2011).

According to Barrangou et al. (2002), even in bacteriophages belonging to the same family there may be differences in the presence of structural proteins, which are unique to each phage and depend on its morphology. It has been suggested that the pattern of expression of individual structural proteins of phages could be used for morphotyping and differentiation of individual phages even within the same family. Differences in the occurrence of particular proteins in bacteriophages may affect their lytic functions, titre stability and other, as yet unknown life functions. In view of the above, the aim of the present study was the isolation and electrophoretic characterization of protein profiles of phages obtained from *M. haemolytica*.

## Materials and methods

### Bacterial strains

The material consisted of the reference strain *M. haemolytica* serotype 1 (ATCC^®^, American Type Culture Collection) BAA-410™, reference serotypes A1 (P588), A2 (499), A5 (P501), A6 (6174), A7, A9 and A11, and wild type isolates of *M. haemolytica* obtained from cattle: 25, 99, 101 and 1480. The wild-type strains were obtained from our own collection, isolated from cattle with clinical symptoms of BRDC (bovine respiratory disease complex). For collection of *M. haemolytica* strains from cattle the authors obtained the consent of the Second Local Ethics Committee for Animal Experiments in Lublin (Approval Number 39/2009, 09 June 2009).

The full genomic sequence of PH2 *M. haemolytica* phage was presented in publication of Highlander et al. (2006). A similar GenBank accession numbers of *M. haemolytica* reference phages have been avaiable also in references Niu et al. (2015).

Bacteria were stored at 85 °C in 50% (v/v) glycerol in brain heart infusion broth (BHIB; Sigma). Isolates were plated on blood agar (BHIA) containing 5% (v/v) sheep’s blood and incubated overnight at 37 °C. The starter cultures were prepared by inoculating a few colonies from the agar plates into 25-mL volumes of BHIB and incubating them overnight at 37 °C with shaking at 120 r.p.m (Davies and Lee 2006).

### Isolation of bacteriophages

Isolation of bacteriophages was carried out according to Davies and Lee (2006). Briefly, 20-mL volumes of BHIB in 100-mL Erlenmeyer flasks were inoculated with 0.2 mL of an overnight bacterial culture of each isolate of *M. haemolytica* and incubated at 37 °C with shaking at 120 rpm. After 4 h of incubation (logarithmic growth phase of bacteria), mitomycin C (Sigma, Ge) was added to a final concentration of 0.3 mg/mL and incubation was continued for a further 12 h. The induction of phages (bacterial cell lysis) was monitored by measuring the optical density (OD 660 nm) after the addition of mitomycin C and comparing it to control bacteria cultures without mitomycin. The phage-rich broth was centrifuged at 3.00×*g* for 20 min at 4 °C and filtered through 0.45 mm Millipore filters. Lytic properties were determined by the double layer plate method according to Huff et al. (2002).

### Electron microscopy analysis

Bacteriophage morphology was examined with a transmission electron microscope using negative-stained slides with 5% uranyl acetate solution (Xie et al. 2005). The slides were examined with a Zeiss LEO 902 electron microscope at an acceleration voltage of 80 kV and a magnification range of 20,000–250,000.

### Preparation of protein extracts of bacteriophages

The 0.5 mL suspension of bacteriophages was treated with 96% EtOH and incubated for 24 h at − 20 °C. Following removal of the ethanol layer, the precipitated protein was resuspended in 100 μl of single-strength SDS-PAGE sample lysis buffer (62.5 mM Tris–HCl, 2% SDS, 6% 2-mercaptoethanol, 10% glycerol, 0.1% bromophenol blue, pH 6.8) and heated at 100 °C for 10 min. Protein concentration was determined by Bradford’s reagent (Sigma-Aldrich, Ge).

### Sodium dodecyl sulphate polyacrylamide gel electrophoresis (SDS-PAGE)

SDS-PAGE electrophoresis was performed according to Laemmli (1970). Separation was carried out in 10% resolving gel (Tris–HCl buffer with pH 8.8), and 4% polyacrylamide in Tris–HCl buffer with pH 6.8 was used as a stacking gel. Electrophoresis was carried out in standard Tris–glycine chamber buffer at a constant current of 100 mA. A molecular weight standard (Perfect TM Color Protein Ladder, EurX, PL) with a molecular weight range from 7 to 240 kDa was used in the electrophoretic separation, after which the gels were stained with Coomassie blue (Sigma, Ge). The stained electropherograms were analysed with a densitometer in Quantity One software (BioRad, Ge).

### Two-dimensional protein analysis (2D electrophoresis)

Immediately before 2D-PAGE analysis, 50 µg of protein from the samples was dissolved in 125 µL of buffer containing 7 M urea, 2 M thiourea, 4% CHAPS and 30 mM TRIS (pH 8.8).

All samples contained 50 µg of protein each were placed on 11 cm IPG strips with a linear pH range of 3–10. The strips were covered with mineral oil and active rehydration was carried out at 50 V for 12 h at 15 °C. Focusing began at 500 V for 30 min, continued at 1000 V for 30 min and was completed at 5000 V until a total value of 4.5 kVh was attained. Focusing was performed in a PROTEAN^®^ i12™ IEF System (Bio-RAD, USA).

After focusing the strips were equilibrated in reducing buffer (100 mM dithiothreitol; DTT, 6 M urea, 30% v/v glycerol, 2% SDS, pH 8.8) twice for 10 min each time, and then in alkylating buffer (150 mM IAA, 6 M urea, 30% v/v glycerol, 2% SDS) twice for 10 min each time at room temp. Second-dimension separation was carried out in a PROTEAN^®^ II xi Cell (Bio-RAD, Warsaw) in 12.5% polyacrylamide gel at a constant current of 25 mA/gel for 6 h at 15 °C (O’Farrell 1975).

Statistical analysis of the 2D electropherograms obtained was performed in ImageMaster 2D Platinum 7.0 software (USA).

### Ultraflex III MALDI TOF/TOF identification of selected common protein spots

Selected spots of interest were excised from the gels, chopped into pieces, and transferred into 0.5 mL tubes. The gel pieces were washed 3 times with 100 µL of 100 mM NH_4_HCO_3_ buffer (pH 8.5) (Sigma, Poznań, Poland) for 5 min. Subsequently, the gel pieces were dehydrated by adding 100 µL ACN and dried in a CentriVap (Labconco, local seller A.G.A Analitical, Warsaw, Poland) (room temperature, 15 min) and allowed to re-swell in 100 µL of 10 mM DTT in 50 mM NH_4_HCO_3_ buffer for the purpose of reduction (56 °C, 60 min). After cooling to room temperature, the solution was replaced by 100 µL of 50 mM iodoacetamide in 50 mM NH_4_HCO_3_ buffer and the gel pieces were incubated in the dark for 45 min at room temperature. The gel pieces were washed three times with 100 mL of 100 mM NH_4_HCO_3_ buffer for 5 min at room temperature, dehydrated with 100 µL ACN, and dried in the CentriVap (Labconco, local seller A.G.A Analytical, Warsaw, Poland) for 15 min.

Enzymatic digestion of the proteins was carried out on ice by stepwise addition of 10 µL of 12.5 ng/mL trypsin (Trypsin Gold, mass spectrometry grade, Promega, Madison, USA) in 50 mM NH_4_HCO_3_ buffer until they were completely rehydrated. Finally, 30 µL of 50 mM NH_4_HCO_3_ buffer was added to keep the gel pieces covered during digestion at 37 °C overnight. After digestion, the supernatant was collected and the peptides were extracted three times with 50 µL of 70% ACN with 1.5% TFA by sonification for 15 min at room temperature in an ultrasonic water bath (Ultron U-507, Ultron, Dywity, Poland) The supernatant was collected and dried in the CentriVap (Labconco, local seller A.G.A Analytical, Warsaw, Poland) for 45 min at 40 °C.

The peptide pellet was suspended in 10 µL of 0.1% TFA and purified with µC18 ZipTip (Eppendorf, Poznań, Poland) according to the manufacturer’s instructions. A 1 µL volume of the purified peptide mixture was placed on a pre-spotted HCCA-PAC (with 3.5-dimethoxy-4-hydroxycinnamic acid) frame (Bruker, Poznań, Poland) and allowed to dry in room temperature. Mass spectra were acquired with an Ultraflex III MALDI TOF/TOF spectrometer (Bruker, Poznań, Poland). Acquisition was performed in positive ion reflector mode with a 25-kV acceleration voltage. Flex analysis 3.0 software (Bruker-Daltonics) was used to select the monoisotopic peptide masses. Peptides and proteins from mass spectrometry data were identified using the MASCOT algorithm (Mascot 2.2 software, Matrix Science Ltd, London, United Kingdom) interrogating the Swiss-Prot database (UniProt release 2016_08) restricted to human taxonomy. Search parameters were set as follows: enzyme—trypsin, modification obligatory—carbamidomethylation cysteine, possible modification—oxidation of methionine, error of 50 ppm (Webster and Oxley 2005).

## Results

### Morphology of bacteriophages

**Fig. 1:**
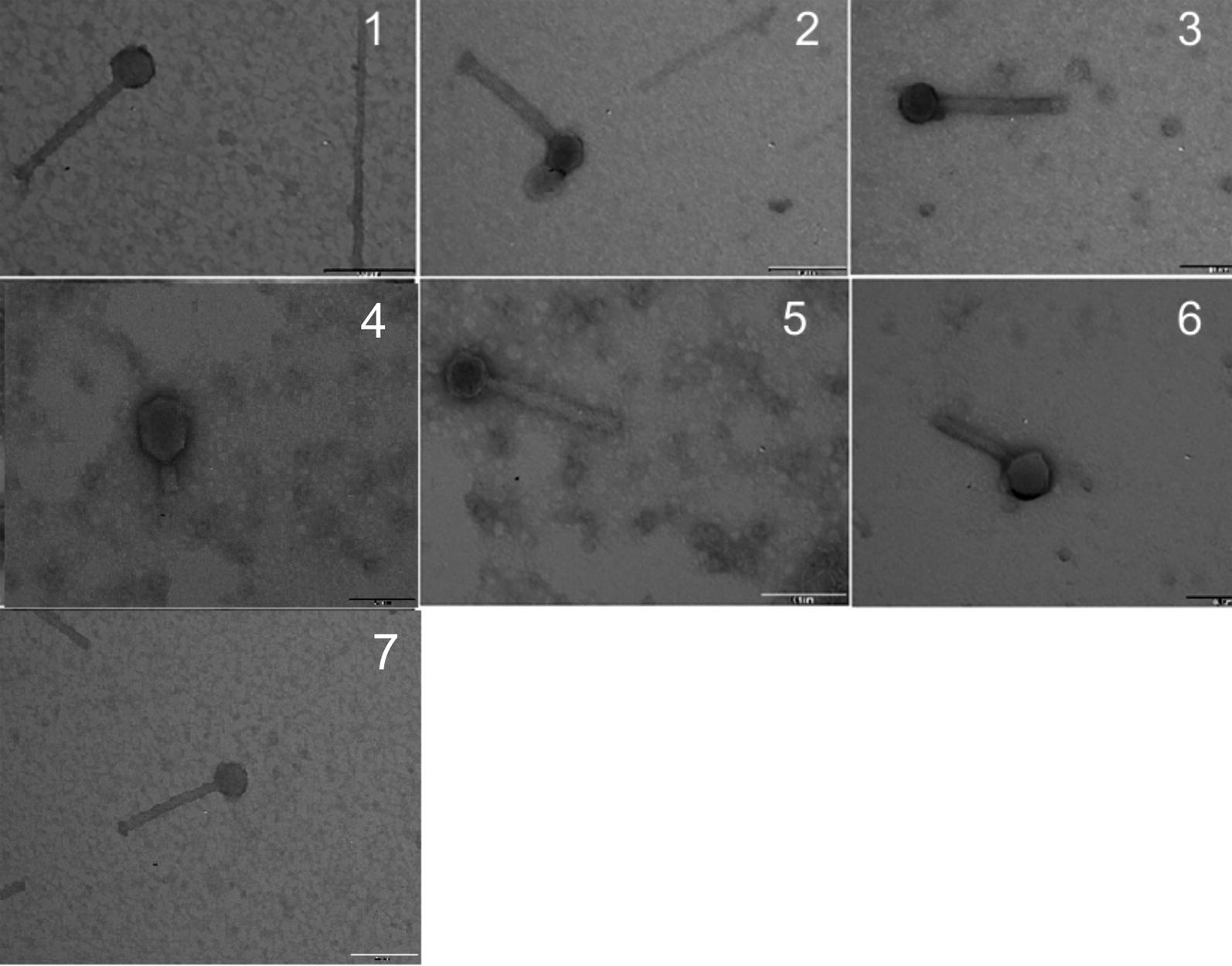
Negative-stained electron micrographs of phages induced in isolates of *M. haemolytica Myoviridae*-type phage. 1—phage induced from *M. haemolytica* BAA-410; 2-φ A1, 2—φ A2; 3—φ A3; 4—φ A5; 5—φ A6; 6—φ A7; 7—φ 25

Among the bacteriophages obtained, seven phages specific for strains A1, A2, A5, A6, A7 and 25 and the reference phage PHL-2 obtained from *M. haemolytica* strain ATCC BAA-410 were set apart for comparative analysis of protein profiles. All bacteriophages subjected to electrophoretic analysis were identified as belonging to the family *Siphoviridae* or *Myoviridae* in the order *Caudovirales* on the basis of their morphological structure. However, only *Myoviridae*-like phages were used for the comparative analysis (Fig. 1).

**Fig. 2:**
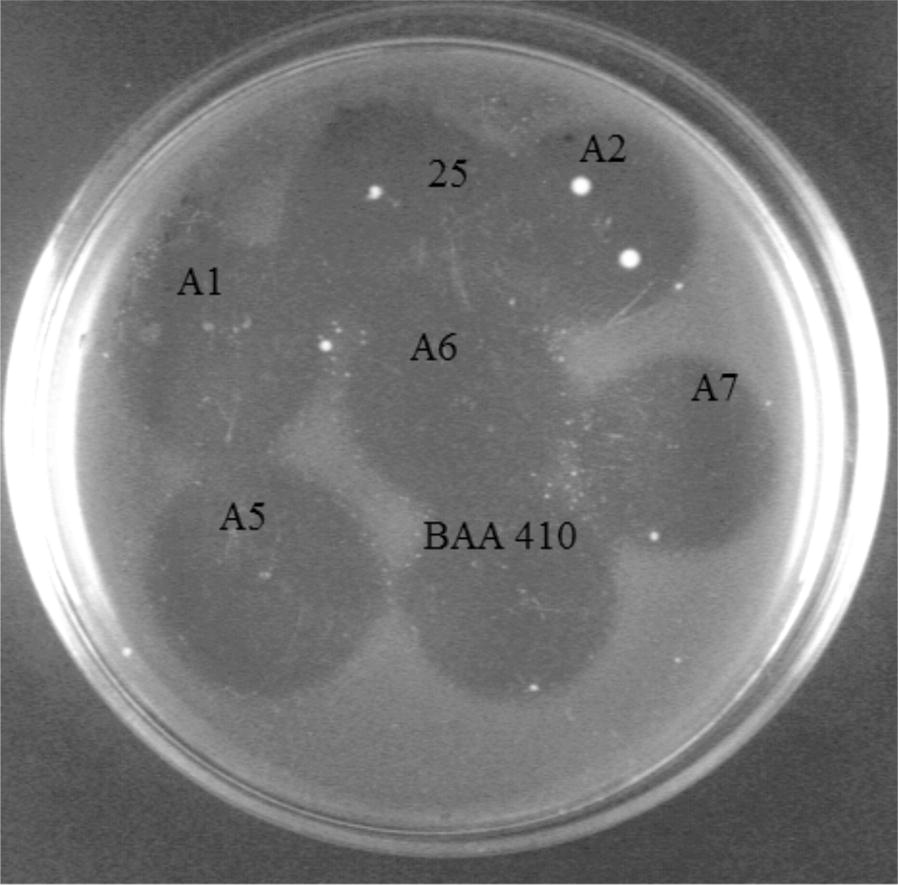
Lytic zones (plaques) of the lysogenic *Myoviridae* phages specific for *M. haemolytica* strains

The bacteriophages chosen for electrophoretic analysis exhibited significant morphological similarity and had lysogenic properties characterized by the formation of cloudy plaques in the form of ring-shaped zones of bacterial growth inhibition on double-layer plates with BHI agar (Fig. 2).

### SDS-PAGE and proteomic results

**Fig. 3:**
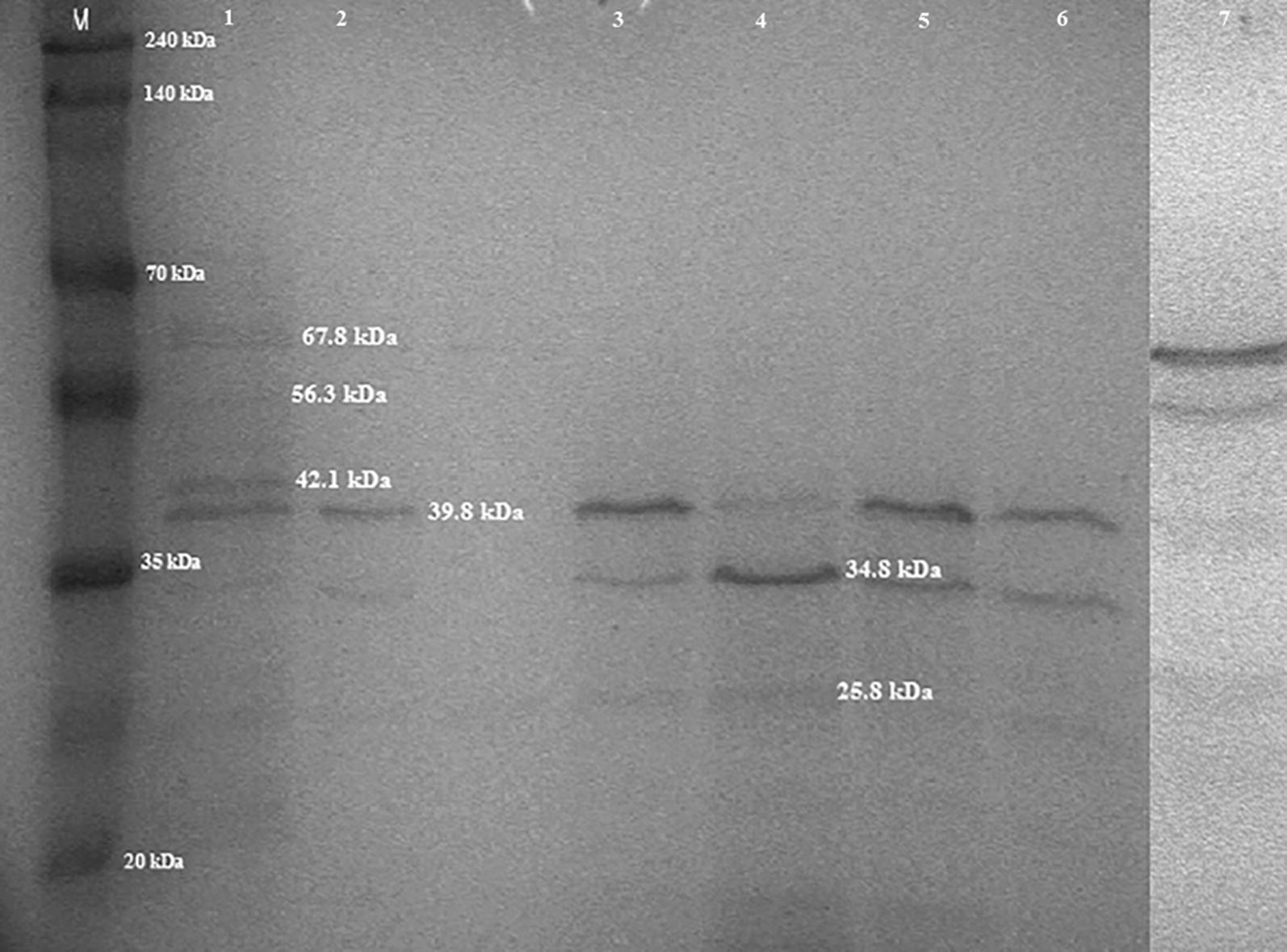
Electrophoretic profiles (SDS-PAGE) of phages of the family *Myoviridae* specific for *M. haemolytica.* M-Perfect™ColorProtein Ladder, EurX, with a mass range from 7 to 240 kDa, Line 1—phage induced from *M. haemolytica* BAA-410; Line 2—φ A1, 2-φ A2; Line 3—φ A3; Line 4—φ A5; Line 5—φ A6; Line 6—φ A7; Line 7—φ25

Analysis of the electrophoregrams in SDS-PAGE electrophoresis revealed the presence of two main protein fractions with molecular weights of 39.8 and 34.8 kDa in all the phage profiles analysed Fig. 3. Moreover, in all profiles a band with a molecular weight of 25.8 kDa was present. The protein profiles obtained for phages A1, A2, A5, A6 and A7 were very similar to one another, but differed from the reference phage in that they lacked protein fractions with molecular weights of 22.9, 56.3 and 73.13 kDa, which were observed in phage PHL-2. In the case of the phage obtained from *M. haemolytica* field strain no. 25 belonging to serotype 1, the protein profile exhibited low similarity to the reference phage PHL-2 like, induced from *M. haemolytica* strain BAA-410 (Fig. 3).

**Fig. 4:**
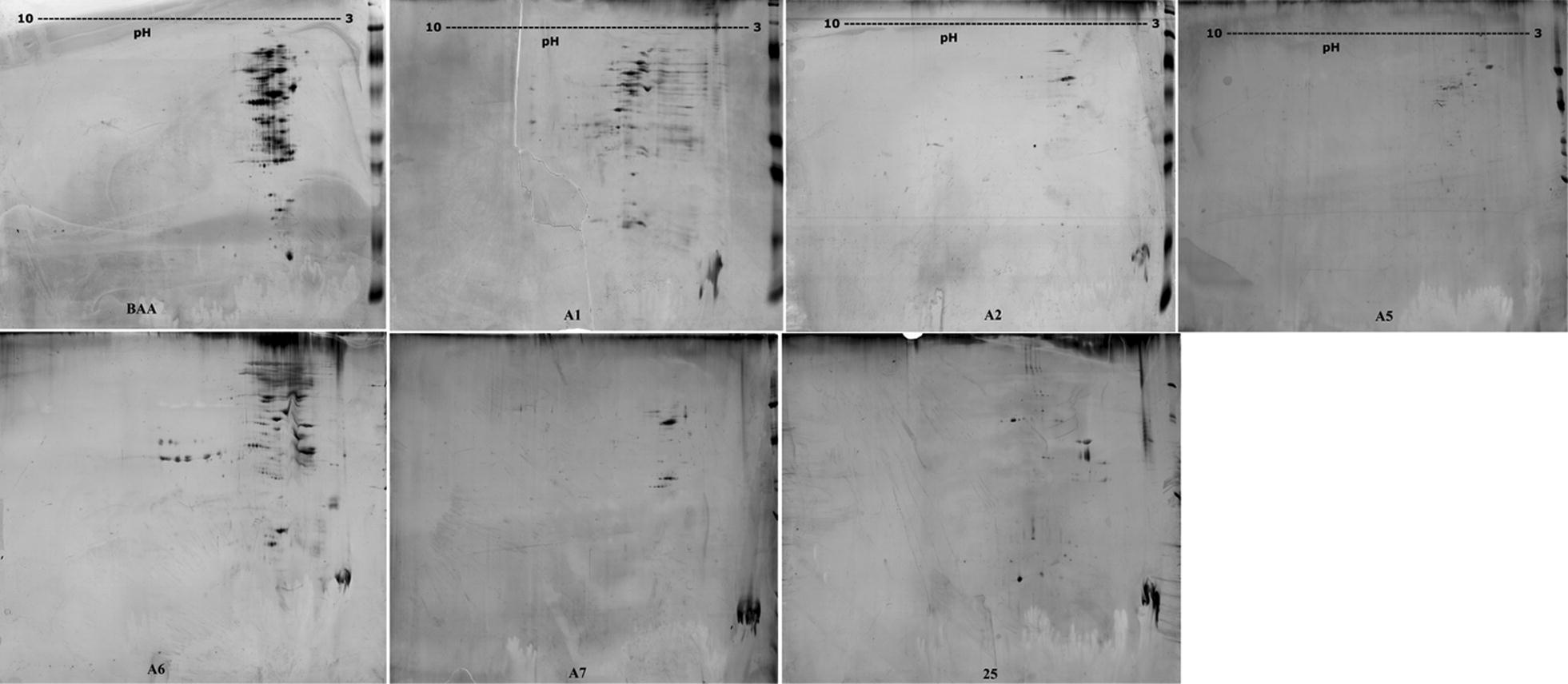
Protein profiles in 2D electrophoretic images from bacteriophages specific for *M. haemolytica* serotype ATCC BAA-410, reference serotypes A1 (P588), A2 (499), A5 (P501), A6 (6174) and A7, and field strain 25 obtained from calves with respiratory syndrome; M—Mol. Weight. Standard 2D (No. Cat. 161-0320, BioRad, USA)

Analysis of the electrophoregrams in 2D electrophoresis revealed significant differences in the size of proteins and their localization in the pH gradient. The most similar protein profiles were observed in the case of phages from strains BAA-410 and A6 Fig. 4. The phages induced from strains A2 and A7 were also very similar. In all profiles the occurrence of two main spots was observed with a molecular weight range from 44 to 70 kDa at pH < 4. Most of the protein spots were in the molecular weight range from 31 to 69 kDa. Statistical analysis of the degree of similarity of the protein profiles (Image Master 2D Platinum 7.0) with the phage obtained from strain BAA-410 showed the highest similarity in the case of the protein profiles of phages induced on strains A6 and BAA-410 and also strains A2 and 25 (Fig. 4).

**Fig. 5:**
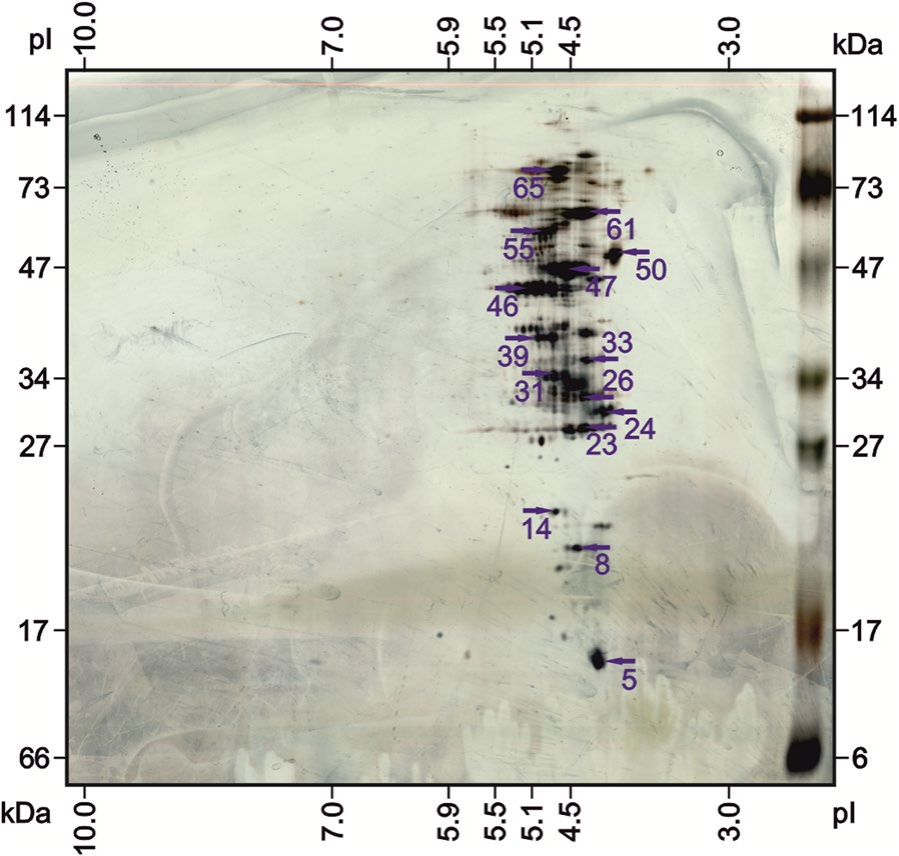
The Protein profiles of spots chosen for MALDI-TOF identification obtained from phages specific for *M. haemolytica* strains

Analysis of selected common protein spots n 2D electrophoresis (Fig. 5) using MALDI-TOF mass spectrometry showed a significant percentage of similarity (more than 70%) in the case of two protein spots with molecular weights of 15.02 and 15.067 kDa. These protein sequences showed high similarity to Late genes activator OS = Bacillus phage B103 and OS = Bacillus phage Nf. One of the spots analysed (17.028 kDa) was more than 40% similar to Gene 29 protein OS = Mycobacterium phage D29. The remaining spots showed a similarity percentage of less than 40%. However, spot number 47 (MW 54.006) was identified as the uncharacterized protein 017L OS = *Mannheimia haemolytica* Mu-like phage (Table 1). All information contained in reports from the database, relevant active pdf files with the results of searches for selected proteins along with matching sequences to reference proteins from databases (SwissProt) have been attached as Additional file 1.

**Table 1:** The MALDI-TOF identification of selected common spots obtained from various phages specific for *M. haemolytica* strains

Spot label	Protein	UniProt	Score	Protein sequence coverage (%)	Mass values matched:	MW (kDa)	pI
5	Gag-Pol polyprotein OS = *Human immunodeficiency virus* type 1 group M subtype J (isolate SE9280)	POL_HV1S2	60	21	23	163,169	8.90
8	Probable tape measure protein OS = *Pseudomonas phage* PAJU2	TMP_BPPAJ	81	17	13	99,205	5.14
14	Large structural phosphoprotein OS = Human *herpesvirus* 7 (strain JI)	P100_HHV7 J	66	16	9	86,698	6.37
19							
23	Major inner protein P1 OS = *Pseudomonas phage* phi6	P1_BPPH6	66	13	9	85,105	6.34
24	Late genes activator OS = *Bacillus phage* B103	VG4_BPB03	72	72	6	15,020	9.66
26	Late genes activator OS = *Bacillus phage* Nf	VG4_BPNF	59	75	5	15,067	9.75
31	Gag-Pol polyprotein OS = *Human immunodeficiency virus* type 1 group M subtype J (isolate SE9280)	POL_HV1S2	114	19	20	163,169	8.90
33	Defense against restriction protein B OS = *Enterobacteria* phage P1	DARB_BPP1	161	13	30	251,899	5.41
39	RNA replication protein OS = *Shallot* virus X	RDRP_SHVX	83	10	15	195,947	8.56
46	RNA replication protein OS = *Shallot* virus X	RDRP_SHVX	115	14	20	195,947	8.56
47	Uncharacterized protein 017L OS = *Mannheimia haemolytica* Mu like phage	017L_FRG3G	59	31	18	54,006	6.15
50	Putative structural protein VP3 OS = *Lymantria dispar* cypovirus 1 (isolate Rao)	VP3_LDCPR	137	18	19	140,252	5.35
55	Antitoxin phd OS = *Enterobacteria* phage P1	PHD_BPP1	77	35	5	8128	5.08
61	DNA-directed DNA polymerase OS = *Vibrio* phage KVP40 (isolate Vibrio parahaemolyticus/Japan/Matsuzaki/1991)	DPOL_BPKVM	93	22	17	98,921	5.59
65	Gene 29 protein OS = *Mycobacterium* phage D29	VG29_BPMD2	72	41	6	17,028	5.61

## Discussion

Analysis of the phage isolates induced from *M. haemolytica* showed slight variation in protein profiles in SDS-PAGE electrophoresis and significant differences in protein profiles in 2D electrophoresis. This is interesting in that the bacteriophages obtained were all identified as belonging to the family *Myoviridae* on the basis of morphological analysis under an electron microscope.

As shown in a study by Jarvis et al. (1993), analysis of the protein profiles of phages in SDS-PAGE electrophoresis can reveal what properties (e.g. morphology, protein or DNA composition, genome structure, or restriction endonuclease pattern) are more conserved than others and which will evolve depending on the type of bacteria they replicate on.

In the present study, in the case of the protein profiles observed in SDS-PAGE electrophoresis, very similar profiles were obtained for 5 phages induced from *M. haemolytica* strains of serotypes A1, A2, A3, A5, A6 and A7, while in the case of one (φ25) the image differed slightly and showed significant similarity to phage PHL2 (BAA410), which confirms earlier research Urban-Chmiel et al. (2015). It should be emphasized here that individual protein fractions were localized within a molecular weight range from 25.8 to 73.13 kDa. The results of the study confirm those obtained by other researchers, who observed certain similarities between phages isolated from different bacteria, often belonging to different species or genera. For example, Jarvis et al. (1993) found that despite the fact that their analysis was conducted on phages obtained from different bacteria—*Bacillus subtilis*, *B. thuringiensis*, *Enterococcus* sp., *Lactobacillus plantarum* and *Staph. aureus*—belonging to the same family *Myoviridae*, the protein profiles obtained in SDS-PAGE electrophoresis were very similar. However, they were not identical and were characterized by the occurrence of 5–7 protein fractions localized in the molecular weight range from 46 to 69 kDa. In a study by Lavigne et al. (2006), using the T7-like *Pseudomonas* bacteriophage pKMV, 12 virion proteins were identified, of which six had previously been identified as structural proteins due to the similarity between structural protein sequences and known phage proteins. Five additional proteins, whose sequences showed little similarity to known phage proteins, were classified as structural proteins. In another study, by Zhang et al. (2006), no differences were observed in the electrophoresis protein profiles of phages specific for *Lactobacillus fermentum*, and the number of protein fractions in the molecular weight range from 25.8 to 79.6 kDa varied from 5 to 7. The authors state that all the phages belonged to the family *Siphoviridae* and had lysogenic properties, and the restriction profiles obtained in restriction analysis were identical and did not allow for a comprehensive characterization. Similarly, in a study by Ngangbam and Devi (2012), SDS-PAGE electrophoretic separation of phages specific for *Salmonella* strains revealed 5 fractions, with molecular weights of 56, 52, 48, and 33 kDa. As the authors point out, similar results were obtained as early as 1978 by other researchers in the case of phages T2 and T384 specific for *E coli* strains (Hantke 1978).

The authors of another study (Radhakrishnan and Ananthasubramanian 2012) conducted on phages specific for *Pseudomonas fluorescens*, suggest that the molecular weights of the major proteins do not vary among phage lysates specific to the same bacterial strains.

In the protein fractions obtained in the present study we also observed the occurrence of main fractions with a molecular weight of 34.8 or 39.8 kDa, and the number of bands obtained ranged from 5 to 7 fractions, which confirms results obtained by other authors (Zhang et al. 2006; Lavigne et al. 2006; Ceyssens et al. 2008).

In the case of the protein profiles obtained in 2D electrophoresis a varied number of spots was observed, corresponding to individual proteins, in numbers ranging from 15 to > 130. In a study by Roberts et al. (2004), 15 main proteins were obtained in 2D analysis of protein profiles of coliphage T1. According to the authors, the range of occurrence of proteins depends on the size, spatial structure and morphology of the phages.

Analysis of the 2D electrophoresis profiles of the phages revealed the presence of proteins common to phages specific for other bacteria, such as *Bacillus*, *Mycobacterium* or *Pseudomonas*.

According to Maxwell and Frappier (2007), identification of the main proteins of the capsid and tail of phages, which are available, for example, in a genome database, may enable morphogenetic annotation of the region. Therefore it can be concluded that two-dimensional analysis of protein profiles in conjunction with mass spectrometry analysis is a more precise tool for comprehensive characterization of bacteriophages, particularly when restriction analysis is inadequate. As wrote Das et al. (2014) using of a combination of molecular biology methods both with conventional microbiological technics is necessary to obtain a better understanding of microbial diversity.

To sum up the results indicate that although the bacteriophages obtained mostly belong to the same family, *Myoviridae*, and were induced on the same species of bacteria, there are differences in their morphological structure and protein profiles, which may influence their virulence, stability and range of lytic activity. Moreover, the profiles obtained exhibit a certain similarity to other phage proteins induced for other bacteria, which was confirmed in the MALDI-TOF analysis and in other studies. We can conclude from the results that the methods used in the present study could be very helpful in differentiating the protein profiles of phages, even those belonging to the same family.

However the presented in this study results were the preliminary study and will be still continued, and after submitting patent applications for the obtained “new” phages, their full amino acid sequences will be made available in GenBank.

## Additional file

**Additional file 1.** Mascot search results( POL_HV1S2): the aminosequences of the obtained bacteriophages proteins.

## Abbreviations

2D electrophoresis: two-dimensional gel electrophoresis
ATCC: American Type Culture Collection
BHIB: brain heart infusion broth
BHIA: brain heart infusion agar
BRDC: bovine respiratory disease complex
DTT: dithiothreitol
SDS PAGE: sodium dodecyl sulfate
