# Supplementary material for: Protein profiles of bacteriophages of the family Myoviridae-like induced on M. haemolytica
Source: AMB Express. 2018 Jun 19;8:102. doi: 10.1186/s13568-018-0630-3 (PMC6008273; doi:10.1186/s13568-018-0630-3)
Supplement: Supplementary file 1 — Additional file 1. Mascot search results( POL_HV1S2): the aminosequences of the obtained bacteriophages proteins. [file 13568_2018_630_MOESM1_ESM.pdf]

MATRIX SCIENCE MASCOT Search Results

Protein View: POL_HV1S2

Gag-Pol polyprotein OS=Human immunodeficiency virus type 1 group M subtype J (isolate SE9280) GN=gag-pol PE=3 SV=3

**Database:**	SwissProt
**Score:**	60
**Expect:**	0.016
**Monoisotopic mass (M_r):**	163169
**Calculated pI:**	8.90
**Taxonomy:**	HIV-1 M:J_SE9280

Sequence similarity is available as an NCBI BLAST search of POL_HV1S2 against nr.

Search parameters

**Enzyme:**	Trypsin: cuts C-term side of KR unless next residue is P.
**Fixed modifications:**	Carbamidomethyl (C)
**Variable modifications:**	Acetyl (N-term), Oxidation (M)
**Mass values searched:**	69
**Mass values matched:**	23

Protein sequence coverage: 21%

Matched peptides shown in ***bold red***.

1 **MGARASILSG GK**LDDWEKIR LRPGGKKKYR IKHLVWASRE LDR**FALNPGL**
51 **LESAKGCQQI LVQLQPALQT GTQEIK**SLYN TVATLYCVHQ RIEIKDTMEA
101 LEKIEEIQNK NKQQAQKAET DKKDNSQVSQ NYPIVQNLQG QPVHQALSPR
151 TLNAWVKVIE EKAFSPEVIP MFSALSEGAT PQDLNTMLNT IGGHQAAMQM
201 LKDTINEEAA EWDRVHPVHA GPIAPGQVRE PRGSDIAGTT STLQEQIGWM
251 TGNPPIPVGE IYKRWIILGL NKIVR**MYSPV SILDIR**QGPK EPFR**DYVDRF**
301 **FK**ALR**AEQAT QDVKNWMTDT LLVQNANPDC K**TILKALGSG ATLEEMMTAC
351 QGVGGPGHKA RVLAEAMSQV TNTNIMMQRG NFRDHKRIVK CFNCGKQGHI
401 AKNCRAPRKK GCWKCGKEGH QMKDCTERQA NFFREDLAFQ QREAR**ELSPE**
451 **QTR**ANSPTSR EPRARR**GDPL PETGAEGQGT VSSNFPQITL WQRPLVTIR**I
501 GGQLR**EALLD TGADDTVLED IDLPR**KWKPK **MIGGIGGFIK VR**QYNEVPIE
551 IEGKKAIGTV LIGPTPVNII GRNMLTQLGC TLNFPISPIE TVPVKLKPGM
601 DGPKIK**QWPL TEEKIK**ALTQ ICAEMEEEGK ISRVGPENPY NTPVFAIKKK
651 DSTKWRK**LVD FRELNKRTQD FWEVQLGIPH PAGLK**KKKSV TVLDVGDAYF
701 SVPLYEDFRK YTAFTIPSIN NETPGIRYQY NVLPQGWK**GS PAIFQCSMTK**
751 **ILKPFR**ERNP EIVIYQYMDD LYVGSDLEIE QHRRKIKELR EHLLKWGFTT
801 PDKKHQKEPP FLWMGYELHP DKWTVQPIQL PEKEDWTVND IQKLVGKLNW
851 ASQIYPGIKV KQLCKLLKGA KALTDIVPLT R**EAELELAEN K**EILKEPVHG
901 VYYDSAKELI AEVQKQGLDQ WTYQIYQEPF KNLKTGKYAK RR**SAHTNDVK**
951 QLAEVVQKIA LEAIVIWGKT PKFRLPIQRE TWETWWTDYW QATWIPEWEF
1001 VNTPPLVKLW YQLEKEPIMG AETFYVDGAS NRETKTGKAG YVTDKGRQKV
1051 VTLTDTTNQK TELHAIYLAL RDSGLEVNIV TDSQYALGII QAQPDKSESE
1101 LVNQIIEELI KKEK**VYLSWV PAHK**GIGGNE QVDKLVSSGI RKVLFLDGID
1151 KAQEDHEKYH SNWRAMASDF NLPPVVAKEI VASCDKCQLK GEAMHGQVDC
1201 SPGIWQLDCT HLEGKVILVA VHVASGYIEA EVIPAETGQE AAFFILKLAG
1251 RWPVK**VIHTD NGSNFTSGAV KAACWWADIK** QEFGIPYNPQ SQGVVESMNK
1301 ELKKIIGQVR EQAEHLKTAV QMAVFIHNFK RK**GGIGGYSA GERIIDIIAT**
1351 **DIQTRELQKQ ITK**IQNFRVY YRDSRDPIWK GPAKLPWKGE GAVVIQDNSE
1401 IKVVPRRKAK IIRDYGK**QMA GDDCVAGR**QD ED

Unformatted sequence string: 1432 residues (for pasting into other applications).

Sort by (●) residue number ( ) increasing mass ( ) decreasing mass
Show (●) matched peptides only ( ) predicted peptides also

Start – End	Observed	Mr(expt)	Mr(calc)	Delta	M	Peptide
1 – 12	1189.6234	1188.6161	1188.6285	-0.0123	1	-.MGARASILSGGK.L + Acetyl (N-term)
1 – 12	1205.6364	1204.6291	1204.6234	0.0057	1	-.MGARASILSGGK.L + Acetyl (N-term); Oxidation (M)
44 – 55	1259.6112	1258.6039	1258.6921	-0.0882	0	R.FALNPGLLESAK.G
44 – 76	3635.8343	3634.8271	3634.9392	-0.1122	1	R.FALNPGLLESAKGCQQILVQLQPALQTGTQEIK.S + Acetyl (N-term)
276 – 286	1293.6560	1292.6487	1292.6798	-0.0312	0	R.MYSPVSILDIR.Q
276 – 286	1309.6603	1308.6530	1308.6748	-0.0217	0	R.MYSPVSILDIR.Q + Oxidation (M)
295 – 302	1089.5564	1088.5492	1088.5291	0.0201	1	R.DYVDRFFK.A
306 – 331	3048.3671	3047.3599	3047.3964	-0.0366	1	R.AEQATQDVKNWMTDTLLVQNANPDCK.T + Acetyl (N-term); Oxidation (M)
446 – 453	1001.4989	1000.4916	1000.4825	0.0091	0	R.ELSPEQTR.A + Acetyl (N-term)
467 – 499	3564.8131	3563.8058	3563.8373	-0.0314	0	R.GDPLPETGAEGQGTVSSNFPQITLWQRPLVTIR.I
506 – 525	2213.0610	2212.0537	2212.0747	-0.0210	0	R.EALLDTGADDTVLEDIDLPR.K + Acetyl (N-term)
531 – 542	1263.6533	1262.6460	1262.7169	-0.0709	1	K.MIGGIGGFIKVR.Q + Oxidation (M)
607 – 616	1271.6012	1270.5940	1270.6921	-0.0981	1	K.QWPLTEEKIK.A
658 – 666	1133.5718	1132.5646	1132.6240	-0.0595	1	K.LVDFRELNK.R
667 – 685	2234.0351	2233.0278	2233.1644	-0.1366	1	K.RTQDFWEVQLGIPHPAGLK.K + Acetyl (N-term)
739 – 756	2122.9798	2121.9726	2122.1067	-0.1342	1	K.GSPAIFQCSMTKILKPFR.E + Acetyl (N-term)
882 – 891	1145.6022	1144.5950	1144.5611	0.0338	0	R.EAELELAENK.E
943 – 950	913.4671	912.4599	912.4301	0.0298	0	R.SAHTNDVK.Q + Acetyl (N-term)
1115 – 1124	1199.5732	1198.5659	1198.6499	-0.0840	0	K.VYLSWVPAHK.G

Start - End	Observed	Mr(expt)	Mr(calc)	Delta	M	Peptide
1256 - 1280	2748.2308	2747.2235	2747.3126	-0.0891	1	K.VIHTDNGSNFTSGAVKAACWWADIK.Q
1333 - 1355	2376.1557	2375.1484	2375.2445	-0.0961	1	K.GGIGGYSAGERIIDIIATDIQTR.E
1356 - 1363	1029.5386	1028.5313	1028.5866	-0.0552	1	R.ELQKQITK.I + Acetyl (N-term)
1418 - 1428	1221.6058	1220.5985	1220.4914	0.1071	0	K.QMAGDDCVAGR.Q + Acetyl (N-term)

No match to: 702.0286, 723.9502, 749.4159, 765.4097, 793.4173, 837.4331, 853.4336, 855.0203, 869.4420, 877.0482, 893.0062, 897.4607, 925.4828, 941.4812, 957.4825, 969.5138, 985.5125, 1013.5398, 1046.5269, 1057.5581, 1066.0327, 1073.5657, 1082.0137, 1101.5873, 1117.5876, 1161.6192, 1177.6093, 1233.6552, 1249.6573, 1337.6968, 2144.9671, 2216.0322, 2232.0431, 2249.0157, 2342.9638, 2398.9862, 2663.2277, 2677.1684, 2691.2113, 2720.1913, 2732.1463, 2760.2360, 2831.1185, 3052.5268, 3323.6010, 3493.7676

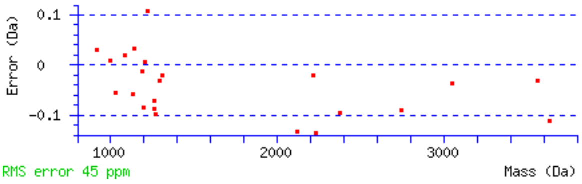

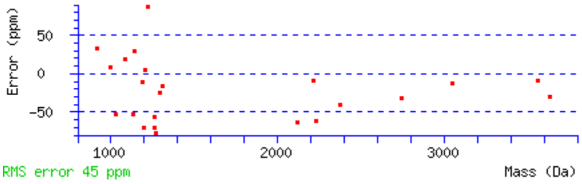


```
ID   POL_HV1S2               Reviewed;        1432 AA.
AC   Q9WC54;
DT   25-JUL-2006, integrated into UniProtKB/Swiss-Prot.
DT   23-JAN-2007, sequence version 3.
DT   28-FEB-2018, entry version 137.
DE   RecName: Full=Gag-Pol polyprotein;
DE   AltName: Full=Pr160Gag-Pol;
DE   Contains:
DE     RecName: Full=Matrix protein p17;
DE              Short=MA;
DE   Contains:
DE     RecName: Full=Capsid protein p24;
DE              Short=CA;
DE   Contains:
DE     RecName: Full=Spacer peptide 1 {ECO:0000250|UniProtKB:P12497};
DE              Short=SP1;
DE     AltName: Full=p2;
DE   Contains:
DE     RecName: Full=Nucleocapsid protein p7;
DE              Short=NC;
DE   Contains:
DE     RecName: Full=Transframe peptide;
DE              Short=TF;
DE   Contains:
DE     RecName: Full=p6-pol;
DE              Short=p6*;
DE   Contains:
DE     RecName: Full=Protease;
DE              EC=3.4.23.16;
DE     AltName: Full=PR;
DE     AltName: Full=Retropepsin;
DE   Contains:
DE     RecName: Full=Reverse transcriptase/ribonuclease H;
DE              EC=2.7.7.49;
DE              EC=2.7.7.7;
DE              EC=3.1.26.13;
DE     AltName: Full=Exoribonuclease H;
DE              EC=3.1.13.2;
DE     AltName: Full=p66 RT;
DE   Contains:
DE     RecName: Full=p51 RT;
DE   Contains:
DE     RecName: Full=p15;
DE   Contains:
DE     RecName: Full=Integrase;
DE              Short=IN;
DE              EC=2.7.7.- {ECO:0000250|UniProtKB:P04585};
DE              EC=3.1.-.- {ECO:0000250|UniProtKB:P04585};
GN   Name=gag-pol;
OS   Human immunodeficiency virus type 1 group M subtype J (isolate SE9280)
OS   (HIV-1).
OC   Viruses; Retro-transcribing viruses; Retroviridae; Orthoretrovirinae;
OC   Lentivirus; Primate lentivirus group.
OX   NCBI_TaxID=388905;
OH   NCBI_TaxID=9606; Homo sapiens (Human).
RN   [1]
RP   NUCLEOTIDE SEQUENCE [GENOMIC DNA].
RX   PubMed=10052760; DOI=10.1089/088922299311475;
RA   Laukkanen T., Albert J., Liitsola K., Green S.D., Carr J.K.,
RA   Leitner T., McCutchan F.E., Salminen M.O.;
RT   "Virtually full-length sequences of HIV type 1 subtype J reference
RT   strains.";
RL   AIDS Res. Hum. Retroviruses 15:293-297(1999).
CC   -!- FUNCTION: Gag-Pol polyprotein: Mediates, with Gag polyprotein, the
CC       essential events in virion assembly, including binding the plasma
CC       membrane, making the protein-protein interactions necessary to
CC       create spherical particles, recruiting the viral Env proteins, and
CC       packaging the genomic RNA via direct interactions with the RNA
CC       packaging sequence (Psi). Gag-Pol polyprotein may regulate its own
CC       translation, by the binding genomic RNA in the 5'-UTR. At low
CC       concentration, the polyprotein would promote translation, whereas
CC       at high concentration, the polyprotein would encapsidate genomic
CC       RNA and then shut off translation. {ECO:0000250}.
CC   -!- FUNCTION: Matrix protein p17: Targets the polyprotein to the
CC       plasma membrane via a multipartite membrane-binding signal, that
CC       includes its myristoylated N-terminus. Matrix protein is part of
CC       the pre-integration complex. Implicated in the release from host
CC       cell mediated by Vpu. Binds to RNA.
CC       {ECO:0000250|UniProtKB:P12497}.
CC   -!- FUNCTION: Capsid protein p24: Forms the conical core that
```

```
CC       encapsulates the genomic RNA-nucleocapsid complex in the virion.
CC       Most core are conical, with only 7% tubular. The core is
CC       constituted by capsid protein hexamer subunits. The core is
CC       disassembled soon after virion entry (By similarity). Host
CC       restriction factors such as TRIM5-alpha or TRIMCyp bind retroviral
CC       capsids and cause premature capsid disassembly, leading to blocks
CC       in reverse transcription. Capsid restriction by TRIM5 is one of
CC       the factors which restricts HIV-1 to the human species. Host PIN1
CC       apparently facilitates the virion uncoating. On the other hand,
CC       interactions with PDZD8 or CYPA stabilize the capsid.
CC       {ECO:0000250|UniProtKB:P04585, ECO:0000250|UniProtKB:P12497}.
CC   -!- FUNCTION: Nucleocapsid protein p7: Encapsulates and protects viral
CC       dimeric unspliced genomic RNA (gRNA). Binds these RNAs through its
CC       zinc fingers. Acts as a nucleic acid chaperone which is involved
CC       in rearangement of nucleic acid secondary structure during gRNA
CC       retrotranscription. Also facilitates template switch leading to
CC       recombination. As part of the polyprotein, participates in gRNA
CC       dimerization, packaging, tRNA incorporation and virion assembly.
CC       {ECO:0000250|UniProtKB:P04585}.
CC   -!- FUNCTION: Protease: Aspartyl protease that mediates proteolytic
CC       cleavages of Gag and Gag-Pol polyproteins during or shortly after
CC       the release of the virion from the plasma membrane. Cleavages take
CC       place as an ordered, step-wise cascade to yield mature proteins.
CC       This process is called maturation. Displays maximal activity
CC       during the budding process just prior to particle release from the
CC       cell. Also cleaves Nef and Vif, probably concomitantly with viral
CC       structural proteins on maturation of virus particles. Hydrolyzes
CC       host EIF4GI and PABP1 in order to shut off the capped cellular
CC       mRNA translation. The resulting inhibition of cellular protein
CC       synthesis serves to ensure maximal viral gene expression and to
CC       evade host immune response (By similarity).
CC       {ECO:0000250|UniProtKB:P04585, ECO:0000255|PROSITE-
CC       ProRule:PRU00275}.
CC   -!- FUNCTION: Reverse transcriptase/ribonuclease H: Multifunctional
CC       enzyme that converts the viral RNA genome into dsDNA in the
CC       cytoplasm, shortly after virus entry into the cell. This enzyme
CC       displays a DNA polymerase activity that can copy either DNA or RNA
CC       templates, and a ribonuclease H (RNase H) activity that cleaves
CC       the RNA strand of RNA-DNA heteroduplexes in a partially processive
CC       3' to 5' endonucleasic mode. Conversion of viral genomic RNA into
CC       dsDNA requires many steps. A tRNA(3)-Lys binds to the primer-
CC       binding site (PBS) situated at the 5'-end of the viral RNA. RT
CC       uses the 3' end of the tRNA primer to perform a short round of
CC       RNA-dependent minus-strand DNA synthesis. The reading proceeds
CC       through the U5 region and ends after the repeated (R) region which
CC       is present at both ends of viral RNA. The portion of the RNA-DNA
CC       heteroduplex is digested by the RNase H, resulting in a ssDNA
CC       product attached to the tRNA primer. This ssDNA/tRNA hybridizes
CC       with the identical R region situated at the 3' end of viral RNA.
CC       This template exchange, known as minus-strand DNA strong stop
CC       transfer, can be either intra- or intermolecular. RT uses the 3'
CC       end of this newly synthesized short ssDNA to perform the RNA-
CC       dependent minus-strand DNA synthesis of the whole template. RNase
CC       H digests the RNA template except for two polypurine tracts (PPTs)
CC       situated at the 5'-end and near the center of the genome. It is
CC       not clear if both polymerase and RNase H activities are
CC       simultaneous. RNase H probably can proceed both in a polymerase-
CC       dependent (RNA cut into small fragments by the same RT performing
CC       DNA synthesis) and a polymerase-independent mode (cleavage of
CC       remaining RNA fragments by free RTs). Secondly, RT performs DNA-
CC       directed plus-strand DNA synthesis using the PPTs that have not
CC       been removed by RNase H as primers. PPTs and tRNA primers are then
CC       removed by RNase H. The 3' and 5' ssDNA PBS regions hybridize to
CC       form a circular dsDNA intermediate. Strand displacement synthesis
CC       by RT to the PBS and PPT ends produces a blunt ended, linear dsDNA
CC       copy of the viral genome that includes long terminal repeats
CC       (LTRs) at both ends. {ECO:0000250|UniProtKB:P04585}.
CC   -!- FUNCTION: Integrase: Catalyzes viral DNA integration into the host
CC       chromosome, by performing a series of DNA cutting and joining
CC       reactions. This enzyme activity takes place after virion entry
CC       into a cell and reverse transcription of the RNA genome in dsDNA.
CC       The first step in the integration process is 3' processing. This
CC       step requires a complex comprising the viral genome, matrix
CC       protein, Vpr and integrase. This complex is called the pre-
CC       integration complex (PIC). The integrase protein removes 2
CC       nucleotides from each 3' end of the viral DNA, leaving recessed CA
CC       OH's at the 3' ends. In the second step, the PIC enters cell
CC       nucleus. This process is mediated through integrase and Vpr
CC       proteins, and allows the virus to infect a non dividing cell. This
CC       ability to enter the nucleus is specific of lentiviruses, other
CC       retroviruses cannot and rely on cell division to access cell
CC       chromosomes. In the third step, termed strand transfer, the
CC       integrase protein joins the previously processed 3' ends to the 5'
CC       ends of strands of target cellular DNA at the site of integration.
CC       The 5'-ends are produced by integrase-catalyzed staggered cuts, 5
CC       bp apart. A Y-shaped, gapped, recombination intermediate results,
CC       with the 5'-ends of the viral DNA strands and the 3' ends of
CC       target DNA strands remaining unjoined, flanking a gap of 5 bp. The
CC       last step is viral DNA integration into host chromosome. This
CC       involves host DNA repair synthesis in which the 5 bp gaps between
CC       the unjoined strands are filled in and then ligated. Since this
CC       process occurs at both cuts flanking the HIV genome, a 5 bp
CC       duplication of host DNA is produced at the ends of HIV-1
CC       integration. Alternatively, Integrase may catalyze the excision of
CC       viral DNA just after strand transfer, this is termed
CC       disintegration. {ECO:0000250|UniProtKB:P04585}.
CC   -!- CATALYTIC ACTIVITY: Specific for a P1 residue that is hydrophobic,
CC       and P1' variable, but often Pro. {ECO:0000255|PROSITE-
CC       ProRule:PRU00275}.
CC   -!- CATALYTIC ACTIVITY: Endohydrolysis of RNA in RNA/DNA hybrids.
CC       Three different cleavage modes: 1. sequence-specific internal
CC       cleavage of RNA. Human immunodeficiency virus type 1 and Moloney
CC       murine leukemia virus enzymes prefer to cleave the RNA strand one
CC       nucleotide away from the RNA-DNA junction. 2. RNA 5'-end directed
```

```
CC       cleavage 13-19 nucleotides from the RNA end. 3. DNA 3'-end
CC       directed cleavage 15-20 nucleotides away from the primer terminus.
CC       {ECO:0000250}.
CC   -!- CATALYTIC ACTIVITY: 3'-end directed exonucleolytic cleavage of
CC       viral RNA-DNA hybrid. {ECO:0000250}.
CC   -!- CATALYTIC ACTIVITY: Deoxynucleoside triphosphate + DNA(n) =
CC       diphosphate + DNA(n+1). {ECO:0000255|PROSITE-ProRule:PRU00405}.
CC   -!- COFACTOR:
CC       Name=Mg(2+); Xref=ChEBI:CHEBI:18420; Evidence={ECO:0000250};
CC       Note=Binds 2 magnesium ions for reverse transcriptase polymerase
CC       activity. {ECO:0000250};
CC   -!- COFACTOR:
CC       Name=Mg(2+); Xref=ChEBI:CHEBI:18420; Evidence={ECO:0000250};
CC       Note=Binds 2 magnesium ions for ribonuclease H (RNase H) activity.
CC       Substrate-binding is a precondition for magnesium binding.
CC       {ECO:0000250};
CC   -!- COFACTOR:
CC       Name=Mg(2+); Xref=ChEBI:CHEBI:18420; Evidence={ECO:0000250};
CC       Note=Magnesium ions are required for integrase activity. Binds at
CC       least 1, maybe 2 magnesium ions. {ECO:0000250};
CC   -!- ENZYME REGULATION: Protease: The viral protease is inhibited by
CC       many synthetic protease inhibitors (PIs), such as amprenavir,
CC       atazanavir, indinavir, loprinavir, nelfinavir, ritonavir and
CC       saquinavir. Use of protease inhibitors in tritherapy regimens
CC       permit more ambitious therapeutic strategies. Reverse
CC       transcriptase/ribonuclease H: RT can be inhibited either by
CC       nucleoside RT inhibitors (NRTIs) or by non nucleoside RT
CC       inhibitors (NNRTIs). NRTIs act as chain terminators, whereas
CC       NNRTIs inhibit DNA polymerization by binding a small hydrophobic
CC       pocket near the RT active site and inducing an allosteric change
CC       in this region. Classical NRTIs are abacavir, adefovir (PMEA),
CC       didanosine (ddI), lamivudine (3TC), stavudine (d4T), tenofovir
CC       (PMPA), zalcitabine (ddC), and zidovudine (AZT). Classical NNRTIs
CC       are atevirdine (BHAP U-87201E), delavirdine, efavirenz (DMP-266),
CC       emivirine (I-EBU), and nevirapine (BI-RG-587). The tritherapies
CC       used as a basic effective treatment of AIDS associate two NRTIs
CC       and one NNRTI. {ECO:0000250}.
CC   -!- SUBUNIT: Matrix protein p17: Homotrimer; further assembles as
CC       hexamers of trimers (By similarity). Matrix protein p17: Interacts
CC       with gp41 (via C-terminus) (By similarity). Matrix protein p17:
CC       interacts with host CALM1; this interaction induces a
CC       conformational change in the Matrix protein, triggering exposure
CC       of the myristate group (By similarity). Matrix protein p17:
CC       interacts with host AP3D1; this interaction allows the polyprotein
CC       trafficking to multivesicular bodies during virus assembly (By
CC       similarity). Matrix protein p17: Part of the pre-integration
CC       complex (PIC) which is composed of viral genome, matrix protein,
CC       Vpr and integrase (By similarity). Capsid protein p24: Homodimer;
CC       the homodimer further multimerizes as homohexamers or
CC       homopentamers. Capsid protein p24: Interacts with human PPIA/CYPA
CC       (By similarity); This interaction stabilizes the capsid. Capsid
CC       protein p24: Interacts with human NUP153 (By similarity). Capsid
CC       protein p24: Interacts with host PDZD8; this interaction
CC       stabilizes the capsid (By similarity). Capsid protein p24:
CC       Interacts with monkey TRIM5; this interaction destabilizes the
CC       capsid (By similarity).Protease: Homodimer, whose active site
CC       consists of two apposed aspartic acid residues. Reverse
CC       transcriptase/ribonuclease H: Heterodimer of p66 RT and p51 RT (RT
CC       p66/p51). Heterodimerization of RT is essential for DNA polymerase
CC       activity. Despite the sequence identities, p66 RT and p51 RT have
CC       distinct folding. Integrase: Homodimer; possibly can form
CC       homotetramer. Integrase: Part of the pre-integration complex (PIC)
CC       which is composed of viral genome, matrix protein, Vpr and
CC       integrase. Integrase: Interacts with human SMARCB1/INI1 and human
CC       PSIP1/LEDGF isoform 1. Integrase: Interacts with human KPNA3; this
CC       interaction might play a role in nuclear import of the pre-
CC       integration complex (By similarity). Integrase: Interacts with
CC       human NUP153; this interaction might play a role in nuclear import
CC       of the pre-integration complex (By similarity).
CC       {ECO:0000250|UniProtKB:P04585, ECO:0000250|UniProtKB:P12497}.
CC   -!- SUBCELLULAR LOCATION: Gag-Pol polyprotein: Host cell membrane;
CC       Lipid-anchor. Host endosome, host multivesicular body. Note=These
CC       locations are linked to virus assembly sites. The main location is
CC       the cell membrane, but under some circumstances, late endosomal
CC       compartments can serve as productive sites for virion assembly.
CC       {ECO:0000250|UniProtKB:P12497}.
CC   -!- SUBCELLULAR LOCATION: Matrix protein p17: Virion membrane; Lipid-
CC       anchor {ECO:0000305}. Host nucleus {ECO:0000250}. Host cytoplasm
CC       {ECO:0000250}.
CC   -!- SUBCELLULAR LOCATION: Capsid protein p24: Virion {ECO:0000305}.
CC   -!- SUBCELLULAR LOCATION: Nucleocapsid protein p7: Virion
CC       {ECO:0000305}.
CC   -!- SUBCELLULAR LOCATION: Reverse transcriptase/ribonuclease H: Virion
CC       {ECO:0000305}.
CC   -!- SUBCELLULAR LOCATION: Integrase: Virion {ECO:0000305}. Host
CC       nucleus {ECO:0000305}. Host cytoplasm {ECO:0000305}. Note=Nuclear
CC       at initial phase, cytoplasmic at assembly. {ECO:0000305}.
CC   -!- ALTERNATIVE PRODUCTS:
CC       Event=Ribosomal frameshifting; Named isoforms=2;
CC         Comment=Translation results in the formation of the Gag
CC         polyprotein most of the time. Ribosomal frameshifting at the
CC         gag-pol genes boundary occurs at low frequency and produces the
CC         Gag-Pol polyprotein. This strategy of translation probably
CC         allows the virus to modulate the quantity of each viral protein.
CC         Maintenance of a correct Gag to Gag-Pol ratio is essential for
CC         RNA dimerization and viral infectivity.;
CC       Name=Gag-Pol polyprotein;
CC         IsoId=Q9WC54-1; Sequence=Displayed;
CC         Note=Produced by -1 ribosomal frameshifting.;
CC       Name=Gag polyprotein;
CC         IsoId=Q9WC53-1; Sequence=External;
CC         Note=Produced by conventional translation.;
CC   -!- DOMAIN: Reverse transcriptase/ribonuclease H: RT is structured in
CC       five subdomains: finger, palm, thumb, connection and RNase H.
```

```
CC       Within the palm subdomain, the 'primer grip' region is thought to
CC       be involved in the positioning of the primer terminus for
CC       accommodating the incoming nucleotide. The RNase H domain
CC       stabilizes the association of RT with primer-template.
CC       {ECO:0000250}.
CC   -!- DOMAIN: Reverse transcriptase/ribonuclease H: The tryptophan
CC       repeat motif is involved in RT p66/p51 dimerization (By
CC       similarity). {ECO:0000250}.
CC   -!- DOMAIN: Integrase: The core domain contains the D-x(n)-D-x(35)-E
CC       motif, named for the phylogenetically conserved glutamic acid and
CC       aspartic acid residues and the invariant 35 amino acid spacing
CC       between the second and third acidic residues. Each acidic residue
CC       of the D,D(35)E motif is independently essential for the 3'-
CC       processing and strand transfer activities of purified integrase
CC       protein. {ECO:0000250}.
CC   -!- PTM: Gag-Pol polyprotein: Specific enzymatic cleavages by the
CC       viral protease yield mature proteins. The protease is released by
CC       autocatalytic cleavage. The polyprotein is cleaved during and
CC       after budding, this process is termed maturation. Proteolytic
CC       cleavage of p66 RT removes the RNase H domain to yield the p51 RT
CC       subunit. Nucleocapsid protein p7 might be further cleaved after
CC       virus entry. {ECO:0000250|UniProtKB:P04585, ECO:0000255|PROSITE-
CC       ProRule:PRU00405}.
CC   -!- PTM: Matrix protein p17: Tyrosine phosphorylated presumably in the
CC       virion by a host kinase. Phosphorylation is apparently not a major
CC       regulator of membrane association. {ECO:0000250|UniProtKB:P04585}.
CC   -!- PTM: Capsid protein p24: Phosphorylated possibly by host MAPK1;
CC       this phosphorylation is necessary for Pin1-mediated virion
CC       uncoating. {ECO:0000250|UniProtKB:P12493}.
CC   -!- PTM: Nucleocapsid protein p7: Methylated by host PRMT6, impairing
CC       its function by reducing RNA annealing and the initiation of
CC       reverse transcription. {ECO:0000250|UniProtKB:P03347}.
CC   -!- MISCELLANEOUS: Reverse transcriptase/ribonuclease H: Error-prone
CC       enzyme that lacks a proof-reading function. High mutations rate is
CC       a direct consequence of this characteristic. RT also displays
CC       frequent template switching leading to high recombination rate.
CC       Recombination mostly occurs between homologous regions of the two
CC       copackaged RNA genomes. If these two RNA molecules derive from
CC       different viral strains, reverse transcription will give rise to
CC       highly recombinated proviral DNAs. {ECO:0000250}.
CC   -!- MISCELLANEOUS: HIV-1 lineages are divided in three main groups, M
CC       (for Major), O (for Outlier), and N (for New, or Non-M, Non-O).
CC       The vast majority of strains found worldwide belong to the group
CC       M. Group O seems to be endemic to and largely confined to Cameroon
CC       and neighboring countries in West Central Africa, where these
CC       viruses represent a small minority of HIV-1 strains. The group N
CC       is represented by a limited number of isolates from Cameroonian
CC       persons. The group M is further subdivided in 9 clades or subtypes
CC       (A to D, F to H, J and K).
CC   -!- MISCELLANEOUS: Resistance to inhibitors associated with mutations
CC       are observed both in viral protease and in reverse transcriptase.
CC       Most of the time, single mutations confer only a modest reduction
CC       in drug susceptibility. Combination of several mutations is
CC       usually required to develop a high-level drug resistance. These
CC       mutations are predominantly found in clade B viruses and not in
CC       other genotypes. They are listed in the clade B representative
CC       isolate HXB2 (AC P04585).
CC   -!- WEB RESOURCE: Name=HIV drug resistance mutations;
CC       URL="https://www.iasusa.org/content/hiv-drug-resistance-mutations";
CC   -!- WEB RESOURCE: Name=hivdb; Note=HIV drug resistance database;
CC       URL="http://hivdb.stanford.edu";
CC   -!- WEB RESOURCE: Name=BioAfrica: HIV bioinformatics in Africa;
CC       URL="http://www.bioafrica.net/index.html";
DR   EMBL; AF082394; AAD17757.1; ALT_SEQ; Genomic_DNA.
DR   ProteinModelPortal; Q9WC54; -.
DR   SMR; Q9WC54; -.
DR   PRO; PR:Q9WC54; -.
DR   GO; GO:0042025; C:host cell nucleus; IEA:UniProtKB-SubCell.
DR   GO; GO:0020002; C:host cell plasma membrane; IEA:UniProtKB-SubCell.
DR   GO; GO:0072494; C:host multivesicular body; IEA:UniProtKB-SubCell.
DR   GO; GO:0019013; C:viral nucleocapsid; IEA:UniProtKB-KW.
DR   GO; GO:0055036; C:virion membrane; IEA:UniProtKB-SubCell.
DR   GO; GO:0004190; F:aspartic-type endopeptidase activity; IEA:UniProtKB-KW.
DR   GO; GO:0003677; F:DNA binding; IEA:UniProtKB-KW.
DR   GO; GO:0003887; F:DNA-directed DNA polymerase activity; IEA:UniProtKB-KW.
DR   GO; GO:0004533; F:exoribonuclease H activity; IEA:UniProtKB-EC.
DR   GO; GO:0008289; F:lipid binding; IEA:UniProtKB-KW.
DR   GO; GO:0003723; F:RNA binding; IEA:UniProtKB-KW.
DR   GO; GO:0003964; F:RNA-directed DNA polymerase activity; IEA:UniProtKB-KW.
DR   GO; GO:0004523; F:RNA-DNA hybrid ribonuclease activity; IEA:InterPro.
DR   GO; GO:0005198; F:structural molecule activity; IEA:InterPro.
DR   GO; GO:0008270; F:zinc ion binding; IEA:InterPro.
DR   GO; GO:0015074; P:DNA integration; IEA:UniProtKB-KW.
DR   GO; GO:0006310; P:DNA recombination; IEA:UniProtKB-KW.
DR   GO; GO:0075713; P:establishment of integrated proviral latency; IEA:UniProtKB-KW.
DR   GO; GO:0039651; P:induction by virus of host cysteine-type endopeptidase activity involved in apoptotic process; IEA:UniProtKB-KW
DR   GO; GO:0039657; P:suppression by virus of host gene expression; IEA:UniProtKB-KW.
DR   GO; GO:0046718; P:viral entry into host cell; IEA:UniProtKB-KW.
DR   GO; GO:0044826; P:viral genome integration into host DNA; IEA:UniProtKB-KW.
DR   GO; GO:0075732; P:viral penetration into host nucleus; IEA:UniProtKB-KW.
DR   CDD; cd05482; HIV_retropepsin_like; 1.
DR   Gene3D; 1.10.10.200; -; 1.
DR   Gene3D; 1.10.1200.30; -; 1.
DR   Gene3D; 1.10.150.90; -; 1.
DR   Gene3D; 1.10.375.10; -; 1.
DR   Gene3D; 2.30.30.10; -; 1.
DR   Gene3D; 2.40.70.10; -; 1.
DR   Gene3D; 3.30.420.10; -; 2.
DR   InterPro; IPR001969; Aspartic_peptidase_AS.
DR   InterPro; IPR000721; Gag_p24.
DR   InterPro; IPR017856; Integrase-like_N.
DR   InterPro; IPR036862; Integrase_C_dom_sf_retrovir.
DR   InterPro; IPR001037; Integrase_C_retrovir.
DR   InterPro; IPR001584; Integrase_cat-core.
```

```
DR   InterPro; IPR003308; Integrase_Zn-bd_dom_N.
DR   InterPro; IPR000071; Lentvrl_matrix_N.
DR   InterPro; IPR012344; Matrix_HIV/RSV_N.
DR   InterPro; IPR001995; Peptidase_A2_cat.
DR   InterPro; IPR021109; Peptidase_aspartic_dom_sf.
DR   InterPro; IPR034170; Retropepsin-like_cat_dom.
DR   InterPro; IPR018061; Retropepsins.
DR   InterPro; IPR008916; Retrov_capsid_C.
DR   InterPro; IPR008919; Retrov_capsid_N.
DR   InterPro; IPR010999; Retrovr_matrix.
DR   InterPro; IPR012337; RNaseH-like_sf.
DR   InterPro; IPR002156; RNaseH_domain.
DR   InterPro; IPR036397; RNaseH_sf.
DR   InterPro; IPR000477; RT_dom.
DR   InterPro; IPR010659; RVT_connect.
DR   InterPro; IPR010661; RVT_thumb.
DR   InterPro; IPR001878; Znf_CCHC.
DR   InterPro; IPR036875; Znf_CCHC_sf.
DR   Pfam; PF00540; Gag_p17; 1.
DR   Pfam; PF00607; Gag_p24; 1.
DR   Pfam; PF00552; IN_DBD_C; 1.
DR   Pfam; PF02022; Integrase_Zn; 1.
DR   Pfam; PF00075; RNase_H; 1.
DR   Pfam; PF00665; rve; 1.
DR   Pfam; PF00077; RVP; 1.
DR   Pfam; PF00078; RVT_1; 1.
DR   Pfam; PF06815; RVT_connect; 1.
DR   Pfam; PF06817; RVT_thumb; 1.
DR   Pfam; PF00098; zf-CCHC; 2.
DR   PRINTS; PR00234; HIV1MATRIX.
DR   SMART; SM00343; ZnF_C2HC; 2.
DR   SUPFAM; SSF46919; SSF46919; 1.
DR   SUPFAM; SSF47836; SSF47836; 1.
DR   SUPFAM; SSF47943; SSF47943; 1.
DR   SUPFAM; SSF50122; SSF50122; 1.
DR   SUPFAM; SSF50630; SSF50630; 1.
DR   SUPFAM; SSF53098; SSF53098; 2.
DR   SUPFAM; SSF57756; SSF57756; 1.
DR   PROSITE; PS50175; ASP_PROT_RETROV; 1.
DR   PROSITE; PS00141; ASP_PROTEASE; 1.
DR   PROSITE; PS50994; INTEGRASE; 1.
DR   PROSITE; PS51027; INTEGRASE_DBD; 1.
DR   PROSITE; PS50879; RNASE_H; 1.
DR   PROSITE; PS50878; RT_POL; 1.
DR   PROSITE; PS50158; ZF_CCHC; 2.
DR   PROSITE; PS50876; ZF_INTEGRASE; 1.
PE   3: Inferred from homology;
KW   Activation of host caspases by virus; AIDS; Aspartyl protease;
KW   Capsid protein; DNA integration; DNA recombination; DNA-binding;
KW   DNA-directed DNA polymerase; Endonuclease;
KW   Eukaryotic host gene expression shutoff by virus;
KW   Eukaryotic host translation shutoff by virus; Host cell membrane;
KW   Host cytoplasm; Host endosome; Host gene expression shutoff by virus;
KW   Host membrane; Host nucleus; Host-virus interaction; Hydrolase;
KW   Lipid-binding; Lipoprotein; Magnesium; Membrane; Metal-binding;
KW   Modulation of host cell apoptosis by virus; Multifunctional enzyme;
KW   Myristate; Nuclease; Nucleotidyltransferase; Phosphoprotein; Protease;
KW   Repeat; Ribosomal frameshifting; RNA-binding;
KW   RNA-directed DNA polymerase; Transferase; Viral genome integration;
KW   Viral nucleoprotein; Viral penetration into host nucleus;
KW   Viral release from host cell; Virion; Virion maturation;
KW   Virus entry into host cell; Zinc; Zinc-finger.
FT   INIT_MET      1      1       Removed; by host. {ECO:0000250}.
FT   CHAIN         2   1432       Gag-Pol polyprotein.
FT                                /FTId=PRO_0000261281.
FT   CHAIN         2    132       Matrix protein p17. {ECO:0000250}.
FT                                /FTId=PRO_0000246556.
FT   CHAIN       133    363       Capsid protein p24. {ECO:0000250}.
FT                                /FTId=PRO_0000246557.
FT   PEPTIDE     364    376       Spacer peptide 1. {ECO:0000250}.
FT                                /FTId=PRO_0000246558.
FT   CHAIN       377    431       Nucleocapsid protein p7. {ECO:0000250}.
FT                                /FTId=PRO_0000246559.
FT   PEPTIDE     432    439       Transframe peptide. {ECO:0000255}.
FT                                /FTId=PRO_0000246731.
FT   CHAIN       440    485       p6-pol. {ECO:0000255}.
FT                                /FTId=PRO_0000246560.
FT   CHAIN       486    584       Protease. {ECO:0000250}.
FT                                /FTId=PRO_0000246561.
FT   CHAIN       585   1144       Reverse transcriptase/ribonuclease H.
FT                                {ECO:0000250}.
FT                                /FTId=PRO_0000246562.
FT   CHAIN       585   1024       p51 RT. {ECO:0000250}.
FT                                /FTId=PRO_0000246563.
FT   CHAIN      1025   1144       p15. {ECO:0000250}.
FT                                /FTId=PRO_0000246564.
FT   CHAIN      1145   1432       Integrase. {ECO:0000250}.
FT                                /FTId=PRO_0000246565.
FT   DOMAIN      505    574       Peptidase A2. {ECO:0000255|PROSITE-
FT                                ProRule:PRU00275}.
FT   DOMAIN      628    818       Reverse transcriptase.
FT                                {ECO:0000255|PROSITE-ProRule:PRU00405}.
FT   DOMAIN     1018   1141       RNase H. {ECO:0000255|PROSITE-
FT                                ProRule:PRU00408}.
FT   DOMAIN     1198   1348       Integrase catalytic.
FT                                {ECO:0000255|PROSITE-ProRule:PRU00457}.
FT   ZN_FING     389    406       CCHC-type 1. {ECO:0000255|PROSITE-
FT                                ProRule:PRU00047}.
FT   ZN_FING     410    427       CCHC-type 2. {ECO:0000255|PROSITE-
FT                                ProRule:PRU00047}.
FT   ZN_FING    1147   1188       Integrase-type. {ECO:0000255|PROSITE-
FT                                ProRule:PRU00450}.
FT   DNA_BIND   1367   1414       Integrase-type. {ECO:0000255|PROSITE-
FT                                ProRule:PRU00506}.
```

```
FT   REGION        7     31       Interaction with Gp41.
FT                                {ECO:0000250|UniProtKB:P12497}.
FT   REGION        8     43       Interaction with host CALM1.
FT                                {ECO:0000250|UniProtKB:P04585}.
FT   REGION       12     19       Interaction with host AP3D1.
FT                                {ECO:0000250|UniProtKB:P12497}.
FT   REGION       14     33       Interaction with membrane
FT                                phosphatidylinositol 4,5-bisphosphate and
FT                                RNA. {ECO:0000250|UniProtKB:P12497}.
FT   REGION       73     77       Interaction with membrane
FT                                phosphatidylinositol 4,5-bisphosphate.
FT                                {ECO:0000250|UniProtKB:P12497}.
FT   REGION      189    227       Interaction with human PPIA/CYPA and
FT                                NUP153. {ECO:0000250|UniProtKB:P12497}.
FT   REGION      277    363       Dimerization/Multimerization of capsid
FT                                protein p24.
FT                                {ECO:0000250|UniProtKB:P04585}.
FT   REGION      486    490       Dimerization of protease.
FT                                {ECO:0000250|UniProtKB:P04585}.
FT   REGION      534    540       Dimerization of protease.
FT                                {ECO:0000250|UniProtKB:P04585}.
FT   REGION      573    585       Dimerization of protease.
FT                                {ECO:0000250|UniProtKB:P04585}.
FT   REGION      811    819       RT 'primer grip'. {ECO:0000250}.
FT   MOTIF        16     22       Nuclear export signal. {ECO:0000250}.
FT   MOTIF        26     32       Nuclear localization signal.
FT                                {ECO:0000250}.
FT   MOTIF       982    998       Tryptophan repeat motif. {ECO:0000250}.
FT   ACT_SITE    510    510       For protease activity; shared with
FT                                dimeric partner. {ECO:0000255|PROSITE-
FT                                ProRule:PRU10094}.
FT   METAL       694    694       Magnesium; catalytic; for reverse
FT                                transcriptase activity. {ECO:0000250}.
FT   METAL       769    769       Magnesium; catalytic; for reverse
FT                                transcriptase activity. {ECO:0000250}.
FT   METAL       770    770       Magnesium; catalytic; for reverse
FT                                transcriptase activity. {ECO:0000250}.
FT   METAL      1027   1027       Magnesium; catalytic; for RNase H
FT                                activity. {ECO:0000250}.
FT   METAL      1062   1062       Magnesium; catalytic; for RNase H
FT                                activity. {ECO:0000250}.
FT   METAL      1082   1082       Magnesium; catalytic; for RNase H
FT                                activity. {ECO:0000250}.
FT   METAL      1133   1133       Magnesium; catalytic; for RNase H
FT                                activity. {ECO:0000250}.
FT   METAL      1208   1208       Magnesium; catalytic; for integrase
FT                                activity. {ECO:0000250}.
FT   METAL      1260   1260       Magnesium; catalytic; for integrase
FT                                activity. {ECO:0000250}.
FT   METAL      1296   1296       Magnesium; catalytic; for integrase
FT                                activity. {ECO:0000250|UniProtKB:P04585}.
FT   SITE        132    133       Cleavage; by viral protease.
FT                                {ECO:0000250}.
FT   SITE        221    222       Cis/trans isomerization of proline
FT                                peptide bond; by human PPIA/CYPA.
FT                                {ECO:0000250}.
FT   SITE        363    364       Cleavage; by viral protease.
FT                                {ECO:0000250}.
FT   SITE        376    377       Cleavage; by viral protease.
FT                                {ECO:0000250}.
FT   SITE        431    432       Cleavage; by viral protease.
FT                                {ECO:0000255}.
FT   SITE        439    440       Cleavage; by viral protease.
FT                                {ECO:0000250}.
FT   SITE        485    486       Cleavage; by viral protease.
FT                                {ECO:0000250}.
FT   SITE        584    585       Cleavage; by viral protease.
FT                                {ECO:0000250}.
FT   SITE        985    985       Essential for RT p66/p51
FT                                heterodimerization. {ECO:0000250}.
FT   SITE        998    998       Essential for RT p66/p51
FT                                heterodimerization. {ECO:0000250}.
FT   SITE       1024   1025       Cleavage; by viral protease; partial.
FT                                {ECO:0000250}.
FT   SITE       1144   1145       Cleavage; by viral protease.
FT                                {ECO:0000250}.
FT   MOD_RES     132    132       Phosphotyrosine; by host. {ECO:0000250}.
FT   LIPID         2      2       N-myristoyl glycine; by host.
FT                                {ECO:0000250}.
SQ   SEQUENCE   1432 AA;  162130 MW;  B0A04035D51B38A3 CRC64;
     MGARASILSG GKLDDWEKIR LRPGGKKKYR IKHLVWASRE LDRFALNPGL LESAKGCQQI
     LVQLQPALQT GTQEIKSLYN TVATLYCVHQ RIEIKDTMEA LEKIEEIQNK NKQQAQKAET
     DKKDNSQVSQ NYPIVQNLQG QPVHQALSPR TLNAWVKVIE EKAFSPEVIP MFSALSEGAT
     PQDLNTMLNT IGGHQAAMQM LKDTINEEAA EWDRVHPVHA GPIAPGQVRE PRGSDIAGTT
     STLQEQIGWM TGNPPIPVGE IYKRWIILGL NKIVRMYSPV SILDIRQGPK EPFRDYVDRF
     FKALRAEQAT QDVKNWMTDT LLVQNANPDC KTILKALGSG ATLEEMMTAC QGVGGPGHKA
     RVLAEAMSQV TNTNIMMQRG NFRDHKRIVK CFNCGKQGHI AKNCRAPRKK GCWKCGKEGH
     QMKDCTERQA NFFREDLAFQ QREARELSPE QTRANSPTSR EPRARRGDPL PETGAEGQGT
     VSSNFPQITL WQRPLVTIRI GGQLREALLD TGADDTVLED IDLPRKWKPK MIGGIGGFIK
     VRQYNEVPIE IEGKKAIGTV LIGPTPVNII GRNMLTQLGC TLNFPISPIE TVPVKLKPGM
     DGPKIKQWPL TEEKIKALTQ ICAEMEEEGK ISRVGPENPY NTPVFAIKKK DSTKWRKLVD
     FRELNKRTQD FWEVQLGIPH PAGLKKKKSV TVLDVGDAYF SVPLYEDFRK YTAFTIPSIN
     NETPGIRYQY NVLPQGWKGS PAIFQCSMTK ILKPFRERNP EIVIYQYMDD LYVGSDLEIE
     QHRRKIKELR EHLLKWGFTT PDKKHQKEPP FLWMGYELHP DKWTVQPIQL PEKEDWTVND
     IQKLVGKLNW ASQIYPGIKV KQLCKLLKGA KALTDIVPLT REAELELAEN KEILKEPVHG
     VYYDSAKELI AEVQKQGLDQ WTYQIYQEPF KNLKTGKYAK RRSAHTNDVK QLAEVVQKIA
     LEAIVIWGKT PKFRLPIQRE TWETWWTDYW QATWIPEWEF VNTPPLVKLW YQLEKEPIMG
     AETFYVDGAS NRETKTGKAG YVTDKGRQKV VTLTDTTNQK TELHAIYLAL RDSGLEVNIV
     TDSQYALGII QAQPDKSESE LVNQIIEELI KKEKVYLSWV PAHKGIGGNE QVDKLVSSGI
     RKVLFLDGID KAQEDHEKYH SNWRAMASDF NLPPVVAKEI VASCDKCQLK GEAMHGQVDC
     SPGIWQLDCT HLEGKVILVA VHVASGYIEA EVIPAETGQE AAFFILKLAG RWPVKVIHTD
     NGSNFTSGAV KAACWWADIK QEFGIPYNPQ SQGVVESMNK ELKKIIGQVR EQAEHLKTAV
     QMAVFIHNFK RKGGIGGYSA GERIIDIIAT DIQTRELQKQ ITKIQNFRVY YRDSRDPIWK
     GPAKLPWKGE GAVVIQDNSE IKVVPRRKAK IIRDYGKQMA GDDCVAGRQD ED
```

Mascot: http://www.matrixscience.com/

MATRIX SCIENCE MASCOT Search Results

Protein View: TMP_BPPAJ

Probable tape measure protein OS=Pseudomonas phage PAJU2 OX=504346 GN=18 PE=1 SV=2

**Database:**	SwissProt
**Score:**	81
**Expect:**	0.00014
**Monoisotopic mass (M_r):**	99205
**Calculated pI:**	5.14
**Taxonomy:**	Pseudomonas phage PAJU2

Sequence similarity is available as an NCBI BLAST search of TMP_BPPAJ against nr.

Search parameters

**Enzyme:**	Trypsin: cuts C-term side of KR unless next residue is P.
**Fixed modifications:**	Carbamidomethyl (C)
**Variable modifications:**	Acetyl (N-term), Oxidation (M)
**Mass values searched:**	22
**Mass values matched:**	13

Protein sequence coverage: 17%

Matched peptides shown in ***bold red***.

```
  1 MATDSLGTLT VDLIANTGGF ERGMDAAERR IASTTRAFQR QEQAAERLVG
 51 RIDPVAGAIN RLVQEQTELE RHFRSGIIPA GEFERLNRIL NDQLDAVQRG
101 NREMASGAMS ARQYQAALRG VPAQFTDIAV SLASGQQPLT VLLQQGGQLK
151 DMFGGVVPAA RALGGYIAGL VNPITGLAAS VGVLGISFID AEREAAAFNK
201 AIFAGNNAAG VSGSGLSQIA EQASAVAGSL SSANKAAIAL ASSGKVAASQ
251 LQSLTEATIA IAQFTGKEVD DVAKSLSAMG DSATDAAAKI SEQYGLLTYE
301 QYQVIKSIDE QGNSQRALDV LGEELNRNAQ ERLKQYRESL SDIERDWIDI
351 KTAITNSYAA VRSEIFPNQN QQIEQIQRIL RTRQEGGVLG AVSSAFGFGE
401 NSTESLQQQL DSLVKQRDAA AKQAEEQAKI TKSNQDRVDA SREWEKENEK
451 YLSSRVKMEK EISAARELGR KAGLNEIEIE DRIAQIRKSY EEKPSSRSGS
501 LDAGQRMLDS LRQQYASMQA QLEATEKLGT QAQALVKWEQ QLADLKSRGS
551 LSADQKALLA NADLITAQLK RNAALEDELN TRKEIQKTLD DYKRLNESLR
601 TDAEKQLDLT RQRFEILDKA RQAGISDDDY RRTAERIVSS STTKAPTFSG
651 VDAVVAGPQG ELDKLDKAQE DLEAWYEQQL EILNENREKR AELNASWDEQ
701 ELKLKQEHED AMAAIEQSRQ QITLSANEQF FGNLSGLAKT FFGEQSGLYK
751 AAFVAEKSFA IAKTLINVPK TASDAYSAMA GIPVIGPALG IAAAAAAVTA
801 QLAQVAAVKN VNLSGMAHDG IDAVPETGTW LLQKGERVTT AETSAKLDKT
851 LDDVRSNQSG GGAPTINLIE DRSRAGQVNT RRQDDQYIID VVVADLFGDG
901 RTSKAIGSSF GMRRSGT
```

Unformatted sequence string: 917 residues (for pasting into other applications).

Sort by ◉ residue number ○ increasing mass ○ decreasing mass
Show ◉ matched peptides only ○ predicted peptides also

Start – End	Observed	Mr(expt)	Mr(calc)	Delta	M	Peptide
100 – 112	1353.7183	1352.7110	1352.5925	0.1185	1	R.GNREMASGAMSAR.Q + Oxidation (M)
151 – 161	1161.6304	1160.6231	1160.5648	0.0583	0	K.DMFGGVVPAAR.A + Acetyl (N-term)
151 – 161	1177.6143	1176.6070	1176.5598	0.0473	0	K.DMFGGVVPAAR.A + Acetyl (N-term); Oxidation (M)
275 – 306	3493.7793	3492.7720	3492.6970	0.0750	1	K.SLSAMGDSATDAAAKISEQYGLLTYEQYQVIK.S + Acetyl (N-term)
307 – 316	1133.5901	1132.5828	1132.5109	0.0720	0	K.SIDEQGNSQR.A
423 – 432	1145.6288	1144.6215	1144.6088	0.0128	1	K.QAEEQAKITK.S
433 – 442	1189.6552	1188.6479	1188.5483	0.0996	1	K.SNQDRVDASR.E + Acetyl (N-term)
461 – 470	1101.6030	1100.5957	1100.5938	0.0019	1	K.EISAARELGR.K
606 – 613	1029.5592	1028.5519	1028.5727	-0.0208	1	K.QLDLTRQR.F
622 – 632	1337.7312	1336.7239	1336.6007	0.1232	1	R.QAGISDDDYRR.T + Acetyl (N-term)
668 – 689	2748.2451	2747.2378	2747.3038	-0.0659	1	K.AQEDLEAWYEQQLEILNENREK.R
835 – 846	1249.6844	1248.6771	1248.6310	0.0461	1	K.GERVTTAETSAK.L
873 – 881	1030.5574	1029.5501	1029.5315	0.0186	1	R.SRAGQVNTR.R + Acetyl (N-term)

No match to: 853.4472, 1001.5121, 1117.6086, 1221.6427, 2220.9695, 2398.9690, 2663.2188, 2691.2078, 3323.6182

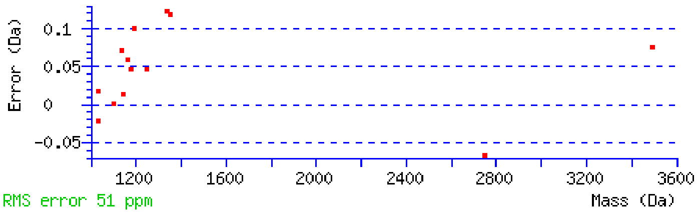

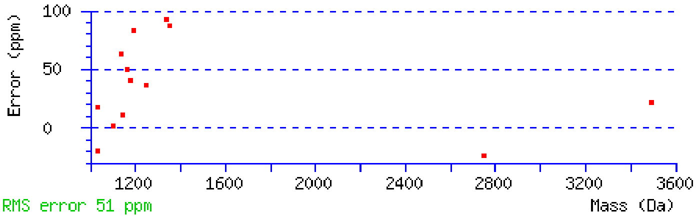


```
ID   TMP_BPPAJ               Reviewed;         917 AA.
AC   P85501; B5WZT4;
DT   08-APR-2008, integrated into UniProtKB/Swiss-Prot.
DT   24-MAR-2009, sequence version 2.
DT   22-NOV-2017, entry version 24.
DE   RecName: Full=Probable tape measure protein {ECO:0000305};
DE   AltName: Full=ORF10 protein {ECO:0000303|PubMed:19010363};
DE   AltName: Full=Structural protein 1 {ECO:0000303|PubMed:19010363};
GN   ORFNames=18 {ECO:0000312|EMBL:BAG75002.1};
OS   Pseudomonas phage PAJU2.
OC   Viruses; dsDNA viruses, no RNA stage; Caudovirales; Siphoviridae.
OX   NCBI_TaxID=504346;
OH   NCBI_TaxID=287; Pseudomonas aeruginosa.
RN   [1]
RP   NUCLEOTIDE SEQUENCE [GENOMIC DNA], PROTEIN SEQUENCE OF 2-21, AND
RP   SUBCELLULAR LOCATION.
RX   PubMed=19010363; DOI=10.1016/j.virusres.2008.10.005;
RA   Uchiyama J., Rashel M., Matsumoto T., Sumiyama Y., Wakiguchi H.,
RA   Matsuzaki S.;
RT   "Characteristics of a novel Pseudomonas aeruginosa bacteriophage,
RT   PAJU2, which is genetically related to bacteriophage D3.";
RL   Virus Res. 139:131-134(2009).
CC   -!- FUNCTION: Probable tape measure protein. Serves as a base for tail
CC       tube protein polymerization and acts as a template for tail length
CC       determination. {ECO:0000305}.
CC   -!- SUBCELLULAR LOCATION: Virion {ECO:0000269|PubMed:19010363}.
CC   -!- SIMILARITY: Belongs to the Lambdalikevirus tape measure protein
CC       family. {ECO:0000305}.
DR   EMBL; AP009624; BAG75002.1; -; Genomic_DNA.
DR   RefSeq; YP_002284352.1; NC_011373.1.
DR   SMR; P85501; -.
DR   GeneID; 6989657; -.
DR   KEGG; vg:6989657; -.
DR   OrthoDB; VOG0900002U; -.
DR   Proteomes; UP000001041; Genome.
DR   GO; GO:0019012; C:virion; IEA:UniProtKB-SubCell.
DR   GO; GO:0098003; P:viral tail assembly; IEA:UniProtKB-KW.
DR   InterPro; IPR009628; Phage_lambda_GpH_tape_meas_N.
DR   Pfam; PF06791; TMP_2; 1.
PE   1: Evidence at protein level;
KW   Coiled coil; Complete proteome; Direct protein sequencing;
KW   Reference proteome; Viral release from host cell; Viral tail assembly;
KW   Virion.
FT   INIT_MET      1      1       Removed. {ECO:0000269|PubMed:19010363}.
FT   CHAIN         2    917       Probable tape measure protein.
FT                                {ECO:0000269|PubMed:19010363}.
FT                                /FTId=PRO_0000326457.
FT   COILED      321    348       {ECO:0000255}.
FT   COILED      400    467       {ECO:0000255}.
FT   COILED      501    546       {ECO:0000255}.
FT   COILED      572    622       {ECO:0000255}.
FT   COILED      665    724       {ECO:0000255}.
SQ   SEQUENCE   917 AA;  99265 MW;  9257CC1FF8E1BC06 CRC64;
     MATDSLGTLT VDLIANTGGF ERGMDAAERR IASTTRAFQR QEQAAERLVG RIDPVAGAIN
     RLVQEQTELE RHFRSGIIPA GEFERLNRIL NDQLDAVQRG NREMASGAMS ARQYQAALRG
     VPAQFTDIAV SLASGQQPLT VLLQQGGQLK DMFGGVVPAA RALGGYIAGL VNPITGLAAS
     VGVLGISFID AEREAAAFNK AIFAGNNAAG VSGSGLSQIA EQASAVAGSL SSANKAAIAL
     ASSGKVAASQ LQSLTEATIA IAQFTGKEVD DVAKSLSAMG DSATDAAAKI SEQYGLLTYE
     QYQVIKSIDE QGNSQRALDV LGEELNRNAQ ERLKQYRESL SDIERDWIDI KTAITNSYAA
     VRSEIFPNQN QQIEQIQRIL RTRQEGGVLG AVSSAFGFGE NSTESLQQQL DSLVKQRDAA
     AKQAEEQAKI TKSNQDRVDA SREWEKENEK YLSSRVKMEK EISAARELGR KAGLNEIEIE
     DRIAQIRKSY EEKPSSRSGS LDAGQRMLDS LRQQYASMQA QLEATEKLGT QAQALVKWEQ
     QLADLKSRGS LSADQKALLA NADLITAQLK RNAALEDELN TRKEIQKTLD DYKRLNESLR
     TDAEKQLDLT RQRFEILDKA RQAGISDDDY RRTAERIVSS STTKAPTFSG VDAVVAGPQG
     ELDKLDKAQE DLEAWYEQQL EILNENREKR AELNASWDEQ ELKLKQEHED AMAAIEQSRQ
     QITLSANEQF FGNLSGLAKT FFGEQSGLYK AAFVAEKSFA IAKTLINVPK TASDAYSAMA
     GIPVIGPALG IAAAAAAVTA QLAQVAAVKN VNLSGMAHDG IDAVPETGTW LLQKGERVTT
     AETSAKLDKT LDDVRSNQSG GGAPTINLIE DRSRAGQVNT RRQDDQYIID VVVADLFGDG
     RTSKAIGSSF GMRRSGT
```

Mascot: http://www.matrixscience.com/

MATRIX SCIENCE MASCOT Search Results

Protein View: P100_HHV7J

Large structural phosphoprotein OS=Human herpesvirus 7 (strain JI) GN=U11 PE=3 SV=1

**Database:**	SwissProt
**Score:**	66
**Expect:**	0.0039
**Monoisotopic mass (M_r):**	86698
**Calculated pI:**	6.37
**Taxonomy:**	Human herpesvirus 7 strain JI

Sequence similarity is available as an NCBI BLAST search of P100_HHV7J against nr.

Search parameters

**Enzyme:**	Trypsin: cuts C-term side of KR unless next residue is P.
**Fixed modifications:**	Carbamidomethyl (C)
**Variable modifications:**	Acetyl (N-term), Oxidation (M)
**Mass values searched:**	19
**Mass values matched:**	9

Protein sequence coverage: 16%

Matched peptides shown in ***bold red***.

1 MKMSHLPFAW ISDEAKCFLS RFFENISSLP VVDIRENPWI LSQCIVKTGN
51 SINNVKTLYN NLILWIYFHQ TLCKKKPDYE EVWQEILKVQ K**ILKDYLEQR**
101 QMITDYSSLT SFNKVGFETE FKNVAK**DLLK** **LGSFLR**WGTV THAADYVNLT
151 TEER**AEIGEN** **LQK**AKNNMLS FTIYQIVDPW NENGYYVTNI NRLLYLGNLL
201 ITLHGSWMNM EKLALNTINE KKNAILKAIE NNKNFVSIYS YQILSLPLTS
251 HRVTSFFK**IL** **TEDFDVITK**S LELHALPVKS TWDDRVKFTP EPIQTFKVLT
301 DLSKSSLSNQ FESSSKKTSH GSSFNPEPFI KTEQRSNNTL SKDLFVGSED
351 GLLSSVKK**DS** **MILDEPR**NST SINNSKKMHR ILQTEILDLT DQTMHRPEDK
401 VNQFNEIAVA PDGINQVIDT LSKLDLHNSN KVIDIVSSPK VNVVQLPKNK
451 IDYHSTFFLP ENEVNRQNGV QSRDQLSKNS TNDLQKILEL RERIKTIKQN
501 NEDIFKLPSE KRRK**EIVHEN** **LQSFDDEHNE** **MSLPPQDQKS** **IK**QKNGNKAN
551 SSTK**TLNMIG** **TNDVNASMKE** **KESASSAKKN** **QLVKDVK**WTP SSSLLDLSRR
601 NDLLQKELFE SGLGEKVKKL LTDFTDTISL EERSLKDVLE PPKKTDVSNI
651 ATFNDNNLKN LLNSRKRDPL FQNFSFTEK**M** **QPVRSPFFLP** **NAEIQDFDSG**
701 **SLLTGK**ETQN TIFGASKAQE NGDKDLIDLE NSVQKDDDIV NKLVSHLTHS
751 EEDVV

Unformatted sequence string: 755 residues (for pasting into other applications).

Sort by ◉ residue number ○ increasing mass ○ decreasing mass
Show ◉ matched peptides only ○ predicted peptides also

Start – End	Observed	Mr(expt)	Mr(calc)	Delta	M	Peptide
92 – 100	1177.5803	1176.5730	1176.6502	-0.0772	1	K.ILKDYLEQR.Q
127 – 136	1161.5974	1160.5901	1160.6917	-0.1016	1	K.DLLKLGSFLR.W
155 – 163	1001.4895	1000.4822	1000.5189	-0.0367	0	R.AEIGENLQK.A
259 – 269	1293.6715	1292.6642	1292.6864	-0.0222	0	K.ILTEDFDVITK.S
359 – 367	1117.5732	1116.5659	1116.5121	0.0538	0	K.DSMILDEPR.N + Acetyl (N-term)
515 – 542	3323.5930	3322.5857	3322.5412	0.0446	1	K.EIVHENLQSFDDEHNEMSLPPQDQKSIK.Q + Oxidation (M)
555 – 571	1897.8770	1896.8697	1896.8921	-0.0224	1	K.TLNMIGTNDVNASMKEK.E + 2 Oxidation (M)
580 – 587	985.5007	984.4934	984.5604	-0.0670	1	K.NQLVKDVK.W + Acetyl (N-term)
680 – 706	3052.4475	3051.4402	3051.5012	-0.0610	1	K.MQPVRSPFFLPNAEIQDFDSGSLLTGK.E + Acetyl (N-term); Oxidation (M)

No match to: 853.4157, 897.4446, 913.4426, 941.4753, 1029.5267, 1221.6063, 2398.8608, 2691.1172, 2831.0339, 3635.8083

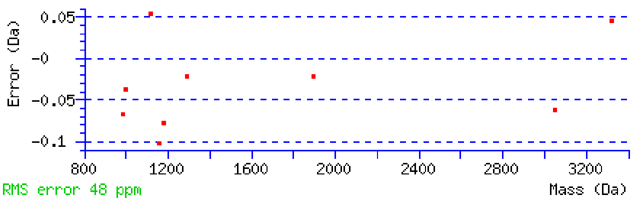

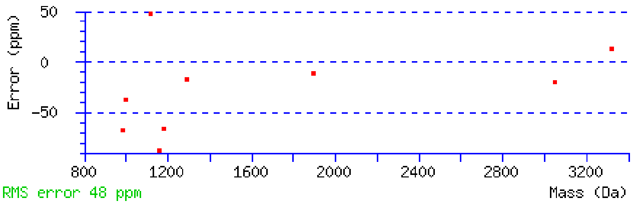


```
ID   P100_HHV7J              Reviewed;         755 AA.
AC   P52519;
DT   01-OCT-1996, integrated into UniProtKB/Swiss-Prot.
DT   01-OCT-1996, sequence version 1.
DT   15-FEB-2017, entry version 40.
DE   RecName: Full=Large structural phosphoprotein;
```

```
DE   AltName: Full=100 kDa phosphoprotein;
DE            Short=pp100;
GN   Name=U11;
OS   Human herpesvirus 7 (strain JI) (HHV-7) (Human T lymphotropic virus).
OC   Viruses; dsDNA viruses, no RNA stage; Herpesvirales; Herpesviridae;
OC   Betaherpesvirinae; Roseolovirus.
OX   NCBI_TaxID=57278;
OH   NCBI_TaxID=9606; Homo sapiens (Human).
RN   [1]
RP   NUCLEOTIDE SEQUENCE [LARGE SCALE GENOMIC DNA].
RX   PubMed=8709220;
RA   Nicholas J.;
RT   "Determination and analysis of the complete nucleotide sequence of
RT   human herpesvirus.";
RL   J. Virol. 70:5975-5989(1996).
CC   -!- SUBCELLULAR LOCATION: Virion tegument {ECO:0000305}. Note=Also
CC       found in dense bodies. {ECO:0000250}.
CC   -!- PTM: Phosphorylated at multiple sites. {ECO:0000250}.
CC   -!- SIMILARITY: Belongs to the herpesviridae large structural
CC       phosphoprotein family. {ECO:0000305}.
DR   EMBL; U43400; AAC54672.1; -; Genomic_DNA.
DR   PIR; T41912; T41912.
DR   OrthoDB; VOG09000030; -.
DR   Proteomes; UP000009246; Genome.
DR   GO; GO:0019033; C:viral tegument; IEA:UniProtKB-SubCell.
DR   GO; GO:0005198; F:structural molecule activity; IEA:InterPro.
DR   InterPro; IPR010340; Herpes_UL11/UL32.
DR   Pfam; PF06070; Herpes_UL32; 1.
PE   3: Inferred from homology;
KW   Complete proteome; Phosphoprotein; Reference proteome; Virion;
KW   Virion tegument.
FT   CHAIN         1    755       Large structural phosphoprotein.
FT                                /FTId=PRO_0000116301.
SQ   SEQUENCE   755 AA;  86580 MW;  4083744CCC3F91DA CRC64;
     MKMSHLPFAW ISDEAKCFLS RFFENISSLP VVDIRENPWI LSQCIVKTGN SINNVKTLYN
     NLILWIYFHQ TLCKKKPDYE EVWQEILKVQ KILKDYLEQR QMITDYSSLT SFNKVGFETE
     FKNVAKDLLK LGSFLRWGTV THAADYVNLT TEERAEIGEN LQKAKNNMLS FTIYQIVDPW
     NENGYYVTNI NRLLYLGNLL ITLHGSWMNM EKLALNTINE KKNAILKAIE NNKNFVSIYS
     YQILSLPLTS HRVTSFFKIL TEDFDVITKS LELHALPVKS TWDDRVKFTP EPIQTFKVLT
     DLSKSSLSNQ FESSSKKTSH GSSFNPEPFI KTEQRSNNTL SKDLFVGSED GLLSSVKKDS
     MILDEPRNST SINNSKKMHR ILQTEILDLT DQTMHRPEDK VNQFNEIAVA PDGINQVIDT
     LSKLDLHNSN KVIDIVSSPK VNVVQLPKNK IDYHSTFFLP ENEVNRQNGV QSRDQLSKNS
     TNDLQKILEL RERIKTIKQN NEDIFKLPSE KRRKEIVHEN LQSFDDEHNE MSLPPQDQKS
     IKQKNGNKAN SSTKTLNMIG TNDVNASMKE KESASSAKKN QLVKDVKWTP SSSLLDLSRR
     NDLLQKELFE SGLGEKVKKL LTDFTDTISL EERSLKDVLE PPKKTDVSNI ATFNDNNLKN
     LLNSRKRDPL FQNFSFTEKM QPVRSPFFLP NAEIQDFDSG SLLTGKETQN TIFGASKAQE
     NGDKDLIDLE NSVQKDDDIV NKLVSHLTHS EEDVV
```

Mascot: http://www.matrixscience.com/

MATRIX SCIENCE MASCOT Search Results

Protein View: P1_BPPH6

Major inner protein P1 OS=Pseudomonas phage phi6 GN=P1 PE=1 SV=1

**Database:**	SwissProt
**Score:**	66
**Expect:**	0.0045
**Monoisotopic mass (M_r):**	85105
**Calculated pI:**	6.34
**Taxonomy:**	Pseudomonas virus phi6

Sequence similarity is available as an NCBI BLAST search of P1_BPPH6 against nr.

Search parameters

**Enzyme:**	Trypsin: cuts C-term side of KR unless next residue is P.
**Fixed modifications:**	Carbamidomethyl (C)
**Variable modifications:**	Acetyl (N-term), Oxidation (M)
**Mass values searched:**	16
**Mass values matched:**	9

Protein sequence coverage: 13%

Matched peptides shown in ***bold red***.

```
  1 MFNLKVKDLN GSARGLTQAF AIGELKNQLS VGALQLPLQF TRTFSASMTS
 51 ELLWEVGKGN IDPVMYARLF FQYAQAGGAL SVDELVNQFT EYHQSTACNP
101 EIWRKLTAYI TGSSNRAIKA DAVGKVPPTA ILEQLRTLAP SEHELFHHIT
151 TDFVCHVLSP LGFILPDAAY VYRVGRTATY PNFYALVDCV RASDLRRMLT
201 ALSSVDSKML QATFKAKGAL APALISQHLA NAATTAFERS RGNFDANAVV
251 SSVLTILGRL WSPSTPKELD PSARLRNTNG IDQLRSNLAL FIAYQDMVKQ
301 RGRAEVIFSD EELSSTIIPW FIEAMSEVSP FKLRPINETT SYIGQTSAID
351 HMGQPSHVVV YEDWQFAKEI TAFTPVKLAN NSNQRFLDVE PGISDRMSAT
401 LAPIGNTFAV SAFVKNRTAV YEAVSQRGTV NSNGAEMTLG FPSVVERDYA
451 LDRDPMVAIA ALRTGIVDES LEARASNDLK RSMFNYYAAV MHYAVAHNPE
501 VVVSEHQGVA AEQGSLYLVW NVRTELRIPV GYNAIEGGSI RTPEPLEAIA
551 YNKPIQPSEV LQAKVLDLAN HTTSIHIWPW HEASTEFAYE DAYSVTIRNK
601 RYTAEVKEFE LLGLGQRRER VRILKPTVAH AIIQMWYSWF VEDDRTLAAA
651 RRTSRDDAEK LAIDGRRMQN AVTLLRKIEM IGTTGIGASA VHLAQSRIVD
701 QMAGRGLIDD SSDLHVGINR HRIRIWAGLA VLQMMGLLSR SEAEALTKVL
751 GDSNALGMVV ATTDIDPSL
```

Unformatted sequence string: 769 residues (for pasting into other applications).

Sort by ◉ residue number ○ increasing mass ○ decreasing mass
Show ◉ matched peptides only ○ predicted peptides also

Start - End	Observed	Mr(expt)	Mr(calc)	Delta	M	Peptide
6 - 14	1001.4946	1000.4873	1000.5301	-0.0428	1	K.VKDLNGSAR.G + Acetyl (N-term)
43 - 68	2902.2605	2901.2532	2901.4041	-0.1509	1	R.TFSASMTSELLWEVGKGNIDPVMYAR.L
59 - 68	1177.5973	1176.5900	1176.5597	0.0303	0	K.GNIDPVMYAR.L + Acetyl (N-term)
260 - 267	957.4589	956.4516	956.4967	-0.0451	0	R.LWSPSTPK.E + Acetyl (N-term)
369 - 377	1047.5011	1046.4938	1046.5648	-0.0710	0	K.EITAFTPVK.L + Acetyl (N-term)
397 - 415	1940.8171	1939.8098	1940.0077	-0.1979	0	R.MSATLAPIGNTFAVSAFVK.N + Oxidation (M)
397 - 417	2210.9851	2209.9778	2210.1518	-0.1739	1	R.MSATLAPIGNTFAVSAFVKNR.T + Oxidation (M)
454 - 474	2227.0227	2226.0154	2226.1678	-0.1524	1	R.DPMVAIAALRTGIVDESLEAR.A
608 - 617	1161.6117	1160.6044	1160.6190	-0.0145	0	K.EFELLGLGQR.R

No match to: 1073.5608, 1221.6163, 2262.9509, 2342.8828, 2368.0435, 2398.9189, 2831.0623

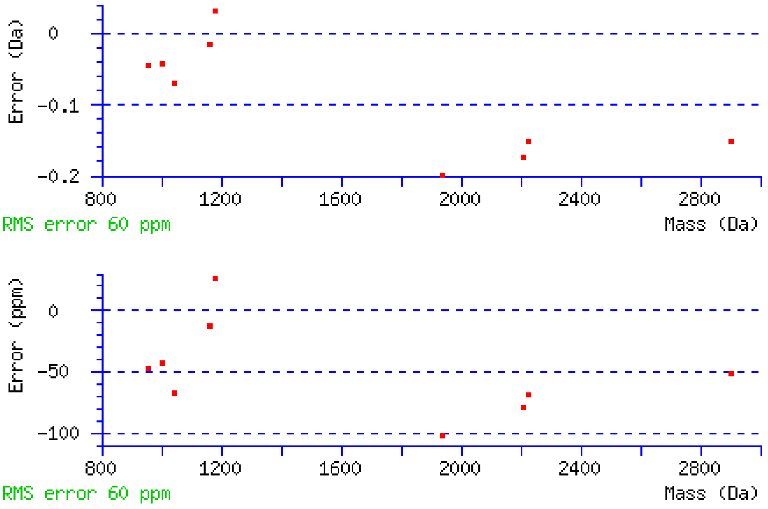


```
ID   P1_BPPH6                Reviewed;         769 AA.
AC   P11126;
DT   01-JUL-1989, integrated into UniProtKB/Swiss-Prot.
DT   01-JUL-1989, sequence version 1.
DT   25-OCT-2017, entry version 65.
DE   RecName: Full=Major inner protein P1;
GN   Name=P1;
OS   Pseudomonas phage phi6 (Bacteriophage phi-6).
OC   Viruses; dsRNA viruses; Cystoviridae; Cystovirus.
OX   NCBI_TaxID=10879;
OH   NCBI_TaxID=319; Pseudomonas savastanoi pv. phaseolicola (Pseudomonas syringae pv. phaseolicola).
RN   [1]
RP   NUCLEOTIDE SEQUENCE [GENOMIC RNA], AND PARTIAL PROTEIN SEQUENCE.
RX   PubMed=3346944;
RA   Mindich L., Nemhauser I., Gottlieb P., Romantschuk M., Carton J.,
RA   Frucht S., Strassman J., Bamford D.H., Kalkkinen N.;
RT   "Nucleotide sequence of the large double-stranded RNA segment of
RT   bacteriophage phi 6: genes specifying the viral replicase and
RT   transcriptase.";
RL   J. Virol. 62:1180-1185(1988).
RN   [2]
RP   FUNCTION, AND RNA-BINDING.
RX   PubMed=14563876; DOI=10.1128/JB.185.21.6409-6414.2003;
RA   Qiao X., Qiao J., Mindich L.;
RT   "Analysis of specific binding involved in genomic packaging of the
RT   double-stranded-RNA bacteriophage phi6.";
RL   J. Bacteriol. 185:6409-6414(2003).
RN   [3]
RP   STRUCTURE BY ELECTRON MICROSCOPY (14.0 ANGSTROMS).
RX   PubMed=17292834; DOI=10.1016/j.str.2006.12.004;
RA   Jaalinoja H.T., Huiskonen J.T., Butcher S.J.;
RT   "Electron cryomicroscopy comparison of the architectures of the
RT   enveloped bacteriophages phi6 and phi8.";
RL   Structure 15:157-167(2007).
CC   -!- FUNCTION: P1 is the major inner capsid (core) protein of the
CC       polyhedral procapsid, which is responsible for genomic replication
CC       and transcription. Forms a dodecahedral shell from 60 asymmetric
CC       dimers. Binds to RNA and may be involved in genomic packaging.
CC       {ECO:0000269|PubMed:14563876}.
CC   -!- SUBUNIT: Homodimer. Associates with the polymerase complex.
CC   -!- INTERACTION:
CC       Self; NbExp=4; IntAct=EBI-15586148, EBI-15586148;
CC   -!- SUBCELLULAR LOCATION: Virion. Note=Inner capsid protein (120
CC       copies).
DR   EMBL; M17461; AAA32357.1; -; Genomic_RNA.
DR   PIR; D29885; P1BPF6.
DR   RefSeq; NP_620348.1; NC_003715.1.
DR   PDB; 4BTG; EM; 4.40 A; A/B=2-761.
DR   PDB; 4BTQ; EM; 7.50 A; A/B=2-761.
DR   PDB; 4K7H; X-ray; 3.60 A; A/B/C/D/E=2-769.
DR   PDB; 5FJ5; EM; 4.80 A; A/B=2-761.
DR   PDB; 5FJ7; EM; 7.90 A; A/B=2-761.
DR   PDB; 5MUU; EM; 4.00 A; A/B=1-769.
DR   PDBsum; 4BTG; -.
DR   PDBsum; 4BTQ; -.
DR   PDBsum; 4K7H; -.
DR   PDBsum; 5FJ5; -.
```

```
DR   PDBsum; 5FJ7; -.
DR   PDBsum; 5MUU; -.
DR   SMR; P11126; -.
DR   DIP; DIP-29115N; -.
DR   IntAct; P11126; 2.
DR   GeneID; 956438; -.
DR   KEGG; vg:956438; -.
DR   Proteomes; UP000002610; Genome.
DR   GO; GO:0039616; C:T=2 icosahedral viral capsid; IEA:UniProtKB-KW.
DR   GO; GO:0039625; C:viral inner capsid; IEA:UniProtKB-KW.
DR   GO; GO:0019013; C:viral nucleocapsid; IEA:UniProtKB-KW.
DR   GO; GO:0019012; C:virion; IDA:CACAO.
DR   GO; GO:0042802; F:identical protein binding; IPI:IntAct.
DR   GO; GO:0003723; F:RNA binding; IEA:UniProtKB-KW.
DR   GO; GO:0006351; P:transcription, DNA-templated; IEA:UniProtKB-KW.
PE   1: Evidence at protein level;
KW   3D-structure; Capsid protein; Complete proteome;
KW   Direct protein sequencing; Inner capsid protein; Reference proteome;
KW   RNA-binding; T=2 icosahedral capsid protein; Transcription;
KW   Viral nucleoprotein; Virion.
FT   CHAIN         1    769       Major inner protein P1.
FT                                /FTId=PRO_0000164637.
SQ   SEQUENCE   769 AA;  84987 MW;  30B7C56BB6BEE326 CRC64;
     MFNLKVKDLN GSARGLTQAF AIGELKNQLS VGALQLPLQF TRTFSASMTS ELLWEVGKGN
     IDPVMYARLF FQYAQAGGAL SVDELVNQFT EYHQSTACNP EIWRKLTAYI TGSSNRAIKA
     DAVGKVPPTA ILEQLRTLAP SEHELFHHIT TDFVCHVLSP LGFILPDAAY VYRVGRTATY
     PNFYALVDCV RASDLRRMLT ALSSVDSKML QATFKAKGAL APALISQHLA NAATTAFERS
     RGNFDANAVV SSVLTILGRL WSPSTPKELD PSARLRNTNG IDQLRSNLAL FIAYQDMVKQ
     RGRAEVIFSD EELSSTIIPW FIEAMSEVSP FKLRPINETT SYIGQTSAID HMGQPSHVVV
     YEDWQFAKEI TAFTPVKLAN NSNQRFLDVE PGISDRMSAT LAPIGNTFAV SAFVKNRTAV
     YEAVSQRGTV NSNGAEMTLG FPSVVERDYA LDRDPMVAIA ALRTGIVDES LEARASNDLK
     RSMFNYYAAV MHYAVAHNPE VVVSEHQGVA AEQGSLYLVW NVRTELRIPV GYNAIEGGSI
     RTPEPLEAIA YNKPIQPSEV LQAKVLDLAN HTTSIHIWPW HEASTEFAYE DAYSVTIRNK
     RYTAEVKEFE LLGLGQRRER VRILKPTVAH AIIQMWYSWF VEDDRTLAAA RRTSRDDAEK
     LAIDGRRMQN AVTLLRKIEM IGTTGIGASA VHLAQSRIVD QMAGRGLIDD SSDLHVGINR
     HRIRIWAGLA VLQMMGLLSR SEAEALTKVL GDSNALGMVV ATTDIDPSL
```

Mascot: http://www.matrixscience.com/

MATRIX SCIENCE MASCOT Search Results

Protein View: VG4_BPB03

Late genes activator OS=Bacillus phage B103 GN=4 PE=3 SV=1

**Database:**	SwissProt
**Score:**	72
**Expect:**	0.0011
**Monoisotopic mass (M_r):**	15020
**Calculated pI:**	9.66
**Taxonomy:**	Bacillus virus B103

Sequence similarity is available as an NCBI BLAST search of VG4_BPB03 against nr.

Search parameters

**Enzyme:**	Trypsin: cuts C-term side of KR unless next residue is P.
**Fixed modifications:**	Carbamidomethyl (C)
**Variable modifications:**	Acetyl (N-term), Oxidation (M)
**Mass values searched:**	19
**Mass values matched:**	6

Protein sequence coverage: 72%

Matched peptides shown in ***bold red***.

```
  1 MPRTARGIYH NLKESEYVVS NGDATFFFSS ELYLNKFLDG YQKHREEFNK
 51 KINRITDTPL NMDMLADITF YSNVEKRGFH TWLKGCNASW QEIHVYALRT
101 MTKPCTQNWS RIRKPKLAER RKNMV
```

Unformatted sequence string: 125 residues (for pasting into other applications).

Sort by ◉ residue number ○ increasing mass ○ decreasing mass
Show ◉ matched peptides only ○ predicted peptides also

Start - End	Observed	Mr(expt)	Mr(calc)	Delta	M	Peptide
14 - 36	2646.1482	2645.1409	2645.2173	-0.0764	0	K.ESEYVVSNGDATFFFSSELYLNK.F
37 - 45	1205.6097	1204.6024	1204.5989	0.0035	1	K.FLDGYQKHR.E + Acetyl (N-term)
44 - 50	1001.4789	1000.4716	1000.4726	-0.0010	1	K.HREEFNK.K + Acetyl (N-term)
52 - 76	2914.2676	2913.2603	2913.4252	-0.1649	1	K.INRITDTPLNMDMLADITFYSNVEK.R
85 - 111	3352.3899	3351.3826	3351.5336	-0.1509	1	K.GCNASWQEIHVYALRTMTKPCTQNWSR.I + Acetyl (N-term); Oxidation (M)
100 - 113	1794.7440	1793.7367	1793.8665	-0.1298	1	R.TMTKPCTQNWSRIR.K + Oxidation (M)

No match to: 793.3773, 869.4139, 941.4612, 941.4612, 1073.5426, 1337.6698, 1353.6659, 2210.9863, 2225.0012, 2465.0530, 2705.0125, 2872.2009, 3052.3933

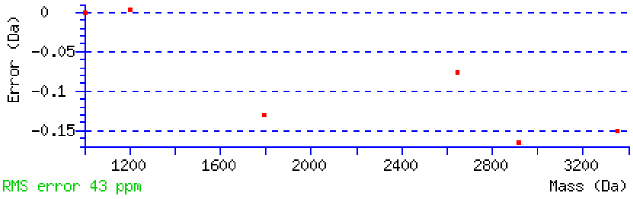

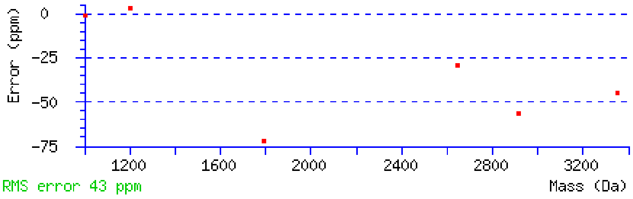


```
ID   VG4_BPB03               Reviewed;         125 AA.
AC   Q37884;
DT   15-DEC-1998, integrated into UniProtKB/Swiss-Prot.
DT   01-NOV-1996, sequence version 1.
DT   25-APR-2018, entry version 74.
DE   RecName: Full=Late genes activator;
DE   AltName: Full=Early protein GP4;
GN   Name=4;
OS   Bacillus phage B103 (Bacteriophage B103).
OC   Viruses; dsDNA viruses, no RNA stage; Caudovirales; Podoviridae;
OC   Picovirinae; Phi29virus.
OX   NCBI_TaxID=10778;
OH   NCBI_TaxID=1423; Bacillus subtilis.
RN   [1]
RP   NUCLEOTIDE SEQUENCE [LARGE SCALE GENOMIC DNA].
RX   PubMed=9358052; DOI=10.1016/S0378-1119(97)00363-6;
RA   Pecenkova T., Benes V., Paces J., Vlcek C., Paces V.;
RT   "Bacteriophage B103: complete DNA sequence of its genome and
RT   relationship to other Bacillus phages.";
RL   Gene 199:157-163(1997).
CC   -!- FUNCTION: This protein is believed to be a positive regulator of
CC       late transcription. It may function as a sigma-like component of
CC       the host RNA polymerase. Binds to a region of the A3 promoter
CC       located between nucleotides -50 and -100 relative to the
CC       transcription start site, that presents a sequence-directed
CC       curvature. Full induction of this curvature is needed for the
```

```
CC       transcription activation process (By similarity). {ECO:0000250}.
CC   -!- SIMILARITY: Belongs to the podoviruses GP4 family. {ECO:0000305}.
DR   EMBL; X99260; CAA67651.1; -; Genomic_DNA.
DR   RefSeq; NP_690637.1; NC_004165.1.
DR   ProteinModelPortal; Q37884; -.
DR   SMR; Q37884; -.
DR   GeneID; 955360; -.
DR   KEGG; vg:955360; -.
DR   OrthoDB; VOG0900014S; -.
DR   Proteomes; UP000000971; Genome.
DR   GO; GO:0003899; F:DNA-directed 5'-3' RNA polymerase activity; IEA:UniProtKB-KW.
DR   GO; GO:2000142; P:regulation of DNA-templated transcription, initiation; IEA:GOC.
DR   Gene3D; 1.10.274.40; -; 1.
DR   InterPro; IPR038246; Phi-29-like_sf.
DR   InterPro; IPR008771; Phi-29_GP4.
DR   Pfam; PF05464; Phi-29_GP4; 1.
DR   ProDom; PD015892; Phi-29_GP4; 1.
PE   3: Inferred from homology;
KW   Activator; Complete proteome; DNA-directed RNA polymerase;
KW   Early protein; Nucleotidyltransferase; Sigma factor; Transcription;
KW   Transcription regulation; Transferase.
FT   CHAIN         1    125       Late genes activator.
FT                                /FTId=PRO_0000106558.
FT   DNA_BIND     77     96       H-T-H motif. {ECO:0000255}.
SQ   SEQUENCE   125 AA;  14915 MW;  678781CE7726FC59 CRC64;
     MPRTARGIYH NLKESEYVVS NGDATFFFSS ELYLNKFLDG YQKHREEFNK KINRITDTPL
     NMDMLADITF YSNVEKRGFH TWLKGCNASW QEIHVYALRT MTKPCTQNWS RIRKPKLAER
     RKNMV
```

Mascot: http://www.matrixscience.com/

MATRIX SCIENCE MASCOT Search Results

Protein View: VG4_BPNF

Late genes activator OS=Bacillus phage Nf GN=4 PE=3 SV=1

**Database:**	SwissProt
**Score:**	59
**Expect:**	0.02
**Monoisotopic mass (M_r):**	15067
**Calculated pI:**	9.75
**Taxonomy:**	Bacillus phage Nf

Sequence similarity is available as an NCBI BLAST search of VG4_BPNF against nr.

Search parameters

**Enzyme:**	Trypsin: cuts C-term side of KR unless next residue is P.
**Fixed modifications:**	Carbamidomethyl (C)
**Variable modifications:**	Acetyl (N-term), Oxidation (M)
**Mass values searched:**	18
**Mass values matched:**	5

Protein sequence coverage: 75%

Matched peptides shown in ***bold red***.

```
  1 MPRTARGIYH NLKESEYVVS NGDATFFFFS EMYQNKFLDG YQKHREEFNK
 51 KINRITDTPL NMDMLADITF YSNVEKRGFH AWLKGVNTTW QEIHVYALRT
101 MTKPCTQNWS RIRKPKLVER RKSMV
```

Unformatted sequence string: 125 residues (for pasting into other applications).

Sort by ◉ residue number ○ increasing mass ○ decreasing mass
Show ◉ matched peptides only ○ predicted peptides also

Start – End	Observed	Mr(expt)	Mr(calc)	Delta	M	Peptide
7 – 36	3564.6838	3563.6765	3563.6344	0.0422	1	R.GIYHNLKESEYVVSNGDATFFFFSEMYQNK.F
37 – 45	1205.5623	1204.5550	1204.5989	-0.0439	1	K.FLDGYQKHR.E + Acetyl (N-term)
44 – 50	1001.5007	1000.4934	1000.4726	0.0208	1	K.HREEFNK.K + Acetyl (N-term)
55 – 77	2745.1177	2744.1104	2744.3037	-0.1933	1	R.ITDTPLNMDMLADITFYSNVEKR.G + Acetyl (N-term); Oxidation (M)
85 – 111	3319.5364	3318.5291	3318.6026	-0.0735	1	K.GVNTTWQEIHVYALRTMTKPCTQNWSR.I + Acetyl (N-term)

No match to: 1045.5043, 1133.5336, 1161.5576, 1221.5609, 1293.6174, 1309.5834, 2083.7600, 2691.0332, 2704.9336, 2748.0718, 3052.3984, 3337.5393, 3340.5208

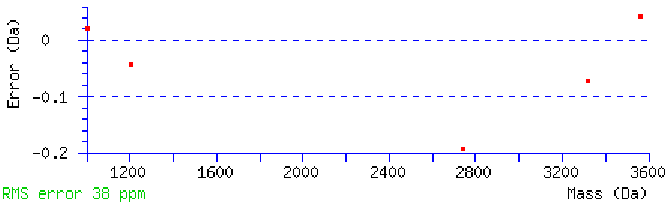

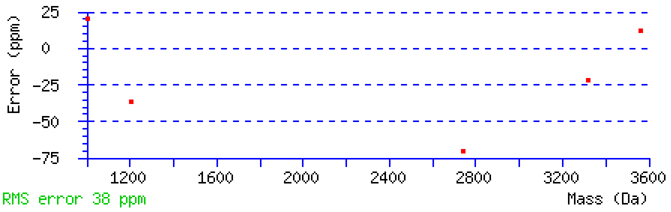


```
ID   VG4_BPNF                Reviewed;         125 AA.
AC   P09877;
DT   01-JUL-1989, integrated into UniProtKB/Swiss-Prot.
DT   01-JUL-1989, sequence version 1.
DT   25-APR-2018, entry version 67.
DE   RecName: Full=Late genes activator;
DE   AltName: Full=Early protein GP4;
DE   AltName: Full=GPF;
GN   Name=4; Synonyms=F;
OS   Bacillus phage Nf (Bacteriophage Nf).
OC   Viruses; dsDNA viruses, no RNA stage; Caudovirales; Podoviridae;
OC   Picovirinae; Phi29virus.
OX   NCBI_TaxID=10753;
OH   NCBI_TaxID=1423; Bacillus subtilis.
RN   [1]
RP   NUCLEOTIDE SEQUENCE [GENOMIC DNA].
RX   PubMed=3015737; DOI=10.1016/0378-1119(86)90302-1;
RA   Mizukami Y., Sekiya T., Hirokawa H.;
RT   "Nucleotide sequence of gene F of Bacillus phage Nf.";
RL   Gene 42:231-235(1986).
CC   -!- FUNCTION: This protein is believed to be a positive regulator of
CC       late transcription. It may function as a sigma-like component of
CC       the host RNA polymerase. Binds to a region of the A3 promoter
CC       located between nucleotides -50 and -100 relative to the
```

```
CC       transcription start site, that presents a sequence-directed
CC       curvature. Full induction of this curvature is needed for the
CC       transcription activation process (By similarity). {ECO:0000250}.
CC   -!- SIMILARITY: Belongs to the podoviruses GP4 family. {ECO:0000305}.
DR   EMBL; M13664; AAA32195.1; -; Genomic_DNA.
DR   PIR; A25643; ERBPNF.
DR   ProteinModelPortal; P09877; -.
DR   SMR; P09877; -.
DR   GO; GO:0003899; F:DNA-directed 5'-3' RNA polymerase activity; IEA:UniProtKB-KW.
DR   GO; GO:2000142; P:regulation of DNA-templated transcription, initiation; IEA:GOC.
DR   Gene3D; 1.10.274.40; -; 1.
DR   InterPro; IPR038246; Phi-29-like_sf.
DR   InterPro; IPR008771; Phi-29_GP4.
DR   Pfam; PF05464; Phi-29_GP4; 1.
DR   ProDom; PD015892; Phi-29_GP4; 1.
PE   3: Inferred from homology;
KW   Activator; DNA-directed RNA polymerase; Early protein;
KW   Nucleotidyltransferase; Sigma factor; Transcription;
KW   Transcription regulation; Transferase.
FT   CHAIN         1    125       Late genes activator.
FT                                /FTId=PRO_0000106559.
FT   DNA_BIND     77     96       H-T-H motif. {ECO:0000255}.
SQ   SEQUENCE   125 AA;  15019 MW;  DBC8AD3EC41A643D CRC64;
     MPRTARGIYH NLKESEYVVS NGDATFFFFS EMYQNKFLDG YQKHREEFNK KINRITDTPL
     NMDMLADITF YSNVEKRGFH AWLKGVNTTW QEIHVYALRT MTKPCTQNWS RIRKPKLVER
     RKSMV
```

Mascot: http://www.matrixscience.com/

MATRIX SCIENCE MASCOT Search Results

Protein View: POL_HV1S2

Gag-Pol polyprotein OS=Human immunodeficiency virus type 1 group M subtype J (isolate SE9280) GN=gag-pol PE=3 SV=3

**Database:**	SwissProt
**Score:**	114
**Expect:**	6.6e-08
**Monoisotopic mass (M_r):**	163169
**Calculated pI:**	8.90
**Taxonomy:**	HIV-1 M:J_SE9280

Sequence similarity is available as an NCBI BLAST search of POL_HV1S2 against nr.

Search parameters

**Enzyme:**	Trypsin: cuts C-term side of KR unless next residue is P.
**Fixed modifications:**	Carbamidomethyl (C)
**Variable modifications:**	Acetyl (N-term), Oxidation (M)
**Mass values searched:**	25
**Mass values matched:**	20

Protein sequence coverage: 19%

Matched peptides shown in ***bold red***.

1 MGARASILSG GKLDDWEKIR LRPGGKKKYR IKHLVWASRE LDRFALNPGL
51 LESAKGCQQI LVQLQPALQT GTQEIKSLYN TVATLYCVHQ RIEIKDTMEA
101 LEKIEEIQNK NKQQAQKAET DKKDNSQVSQ NYPIVQNLQG QPVHQALSPR
151 TLNAWVKVIE EKAFSPEVIP MFSALSEGAT PQDLNTMLNT IGGHQAAMQM
201 LKDTINEEAA EWDRVHPVHA GPIAPGQVRE PRGSDIAGTT STLQEQIGWM
251 TGNPPIPVGE IYKRWIILGL NKIVR**MYSPV** **SILDIR**QGPK EPFR**DYVDRF**
301 **FK**ALRAEQAT QDVKNWMTDT LLVQNANPDC K**TILKALGSG** **ATLEEMMTAC**
351 **QGVGGPGHK**A RVLAEAMSQV TNTNIMMQRG NFRDHKRIVK CFNCGKQGHI
401 AKNCRAPRKK GCWKCGKEGH QMKDCTERQA NFFREDLAFQ QREAR**ELSPE**
451 **QTR**ANSPTSR EPRARRGDPL PETGAEGQGT VSSNFPQITL WQRPLVTIRI
501 GGQLR**EALLD** **TGADDTVLED** **IDLPRK**WKPK **MIGGIGGFIK** **VR**QYNEVPIE
551 IEGK**KAIGTV** **LIGPTPVNII** **GR**NMLTQLGC TLNFPISPIE TVPVKLKPGM
601 DGPKIKQWPL TEEKIKALTQ ICAEMEEEGK ISRVGPENPY NTPVFAIKKK
651 DSTKWRK**LVD** **FRELNK**RTQD FWEVQLGIPH PAGLKKKKSV TVLDVGDAYF
701 SVPLYEDFRK **YTAFTIPSIN** **NETPGIRYQY** **NVLPQGWK**GS PAIFQCSMTK
751 ILKPFR**ERNP** **EIVIYQYMDD** **LYVGSDLEIE** **QHR**RKIKELR EHLLKWGFTT
801 PDKKHQKEPP FLWMGYELHP DKWTVQPIQL PEKEDWTVND IQKLVGKLNW
851 ASQIYPGIKV KQLCKLLKGA KALTDIVPLT REAELELAEN KEILKEPVHG
901 VYYDSAKELI AEVQK**QGLDQ** **WTYQIYQEPF** **KNLK**TGKYAK RRSAHTNDVK
951 QLAEVVQKIA LEAIVIWGKT PKFRLPIQRE TWETWWTDYW QATWIPEWEF
1001 VNTPPLVK**LW** **YQLEKEPIMG** **AETFYVDGAS** **NR**ETKTGKAG YVTDKGRQKV
1051 VTLTDTTNQK TELHAIYLAL RDSGLEVNIV TDSQYALGII QAQPDKSESE
1101 LVNQIIEELI KKEKVYLSWV PAHKGIGGNE QVDKLVSSGI RK**VLFLDGID**
1151 **K**AQEDHEKYH SNWRAMASDF NLPPVVAKEI VASCDKCQLK GEAMHGQVDC
1201 SPGIWQLDCT HLEGKVILVA VHVASGYIEA EVIPAETGQE AAFFILKLAG
1251 RWPVKVIHTD NGSNFTSGAV KAACWWADIK **QEFGIPYNPQ** **SQGVVESMNK**
1301 **ELK**KIIGQVR EQAEHLKTAV QMAVFIHNFK R**KGGIGGYSA** **GER**IIDIIAT
1351 DIQTR**ELQKQ** **ITK**IQNFRVY YRDSRDPIWK GPAKLPWKGE GAVVIQDNSE
1401 IKVVPRRKAK IIRDYGK**QMA** **GDDCVAGR**QD ED

Unformatted sequence string: 1432 residues (for pasting into other applications).

Sort by ◉ residue number ○ increasing mass ○ decreasing mass
Show ◉ matched peptides only ○ predicted peptides also

Start – End	Observed	Mr(expt)	Mr(calc)	Delta	M	Peptide
276 – 286	1309.7163	1308.7090	1308.6748	0.0343	0	R.MYSPVSILDIR.Q + Oxidation (M)
295 – 302	1089.6641	1088.6568	1088.5291	0.1277	1	R.DYVDRFFK.A
332 – 359	2888.4236	2887.4163	2887.3878	0.0285	1	K.TILKALGSGATLEEMMTACQGVGGPGHK.A + Acetyl (N-term); 2 Oxidation (M)
446 – 453	1001.6478	1000.6405	1000.4825	0.1580	0	R.ELSPEQTR.A + Acetyl (N-term)
506 – 525	2213.0686	2212.0613	2212.0747	-0.0134	0	R.EALLDTGADDTVLEDIDLPR.K + Acetyl (N-term)
506 – 526	2299.1052	2298.0979	2298.1591	-0.0611	1	R.EALLDTGADDTVLEDIDLPRK.W
531 – 542	1263.7682	1262.7609	1262.7169	0.0440	1	K.MIGGIGGFIKVR.Q + Oxidation (M)
555 – 572	1860.9182	1859.9109	1860.1197	-0.2087	1	K.KAIGTVLIGPTPVNIIGR.N + Acetyl (N-term)
658 – 666	1133.6858	1132.6785	1132.6240	0.0545	1	K.LVDFRELNK.R
711 – 738	3312.4807	3311.4734	3311.6615	-0.1881	1	K.YTAFTIPSINNETPGIRYQYNVLPQGWK.G + Acetyl (N-term)
757 – 783	3340.7678	3339.7605	3339.5717	0.1888	1	R.ERNPEIVIYQYMDDLYVGSDLEIEQHR.R + Oxidation (M)
916 – 934	2399.0544	2398.0471	2398.1957	-0.1486	1	K.QGLDQWTYQIYQEPFKNLK.T
1009 – 1032	2875.4270	2874.4197	2874.3534	0.0663	1	K.LWYQLEKEPIMGAETFYVDGASNR.E + Acetyl (N-term); Oxidation (M)
1143 – 1151	1061.6228	1060.6155	1060.5805	0.0351	0	K.VLFLDGIDK.A + Acetyl (N-term)
1281 – 1303	2680.4019	2679.3946	2679.2850	0.1096	1	K.QEFGIPYNPQSQGVVESMNKELK.K + Acetyl (N-term); Oxidation (M)
1332 – 1343	1193.6840	1192.6767	1192.5836	0.0931	1	R.KGGIGGYSAGER.I + Acetyl (N-term)
1356 – 1363	1029.6829	1028.6756	1028.5866	0.0890	1	R.ELQKQITK.I + Acetyl (N-term)
1418 – 1428	1179.6957	1178.6884	1178.4808	0.2076	0	K.QMAGDDCVAGR.Q

Start – End	Observed	Mr(expt)	Mr(calc)	Delta	M	Peptide
1418 – 1428	1221.7084	1220.7011	1220.4914	0.2097	0	K.QMAGDDCVAGR.Q + Acetyl (N-term)
1418 – 1428	1237.6913	1236.6840	1236.4863	0.1977	0	K.QMAGDDCVAGR.Q + Acetyl (N-term); Oxidation (M)

No match to: 1073.7023, 2384.1018, 2924.5049, 3052.7234, 3056.6763

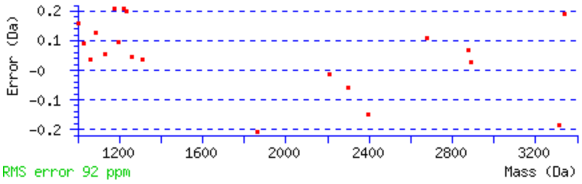

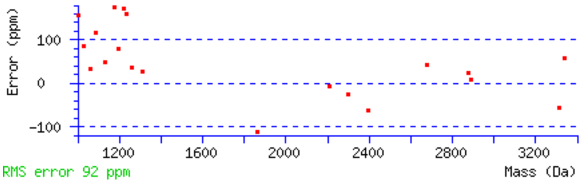


```
ID   POL_HV1S2               Reviewed;        1432 AA.
AC   Q9WC54;
DT   25-JUL-2006, integrated into UniProtKB/Swiss-Prot.
DT   23-JAN-2007, sequence version 3.
DT   28-FEB-2018, entry version 137.
DE   RecName: Full=Gag-Pol polyprotein;
DE   AltName: Full=Pr160Gag-Pol;
DE   Contains:
DE     RecName: Full=Matrix protein p17;
DE              Short=MA;
DE   Contains:
DE     RecName: Full=Capsid protein p24;
DE              Short=CA;
DE   Contains:
DE     RecName: Full=Spacer peptide 1 {ECO:0000250|UniProtKB:P12497};
DE              Short=SP1;
DE     AltName: Full=p2;
DE   Contains:
DE     RecName: Full=Nucleocapsid protein p7;
DE              Short=NC;
DE   Contains:
DE     RecName: Full=Transframe peptide;
DE              Short=TF;
DE   Contains:
DE     RecName: Full=p6-pol;
DE              Short=p6*;
DE   Contains:
DE     RecName: Full=Protease;
DE              EC=3.4.23.16;
DE     AltName: Full=PR;
DE     AltName: Full=Retropepsin;
DE   Contains:
DE     RecName: Full=Reverse transcriptase/ribonuclease H;
DE              EC=2.7.7.49;
DE              EC=2.7.7.7;
DE              EC=3.1.26.13;
DE     AltName: Full=Exoribonuclease H;
DE              EC=3.1.13.2;
DE     AltName: Full=p66 RT;
DE   Contains:
DE     RecName: Full=p51 RT;
DE   Contains:
DE     RecName: Full=p15;
DE   Contains:
DE     RecName: Full=Integrase;
DE              Short=IN;
DE              EC=2.7.7.- {ECO:0000250|UniProtKB:P04585};
DE              EC=3.1.-.- {ECO:0000250|UniProtKB:P04585};
GN   Name=gag-pol;
OS   Human immunodeficiency virus type 1 group M subtype J (isolate SE9280)
OS   (HIV-1).
OC   Viruses; Retro-transcribing viruses; Retroviridae; Orthoretrovirinae;
OC   Lentivirus; Primate lentivirus group.
OX   NCBI_TaxID=388905;
OH   NCBI_TaxID=9606; Homo sapiens (Human).
RN   [1]
RP   NUCLEOTIDE SEQUENCE [GENOMIC DNA].
RX   PubMed=10052760; DOI=10.1089/088922299311475;
RA   Laukkanen T., Albert J., Liitsola K., Green S.D., Carr J.K.,
RA   Leitner T., McCutchan F.E., Salminen M.O.;
RT   "Virtually full-length sequences of HIV type 1 subtype J reference
RT   strains.";
RL   AIDS Res. Hum. Retroviruses 15:293-297(1999).
CC   -!- FUNCTION: Gag-Pol polyprotein: Mediates, with Gag polyprotein, the
CC       essential events in virion assembly, including binding the plasma
CC       membrane, making the protein-protein interactions necessary to
CC       create spherical particles, recruiting the viral Env proteins, and
CC       packaging the genomic RNA via direct interactions with the RNA
CC       packaging sequence (Psi). Gag-Pol polyprotein may regulate its own
CC       translation, by the binding genomic RNA in the 5'-UTR. At low
CC       concentration, the polyprotein would promote translation, whereas
CC       at high concentration, the polyprotein would encapsidate genomic
CC       RNA and then shut off translation. {ECO:0000250}.
CC   -!- FUNCTION: Matrix protein p17: Targets the polyprotein to the
CC       plasma membrane via a multipartite membrane-binding signal, that
CC       includes its myristoylated N-terminus. Matrix protein is part of
CC       the pre-integration complex. Implicated in the release from host
CC       cell mediated by Vpu. Binds to RNA.
CC       {ECO:0000250|UniProtKB:P12497}.
CC   -!- FUNCTION: Capsid protein p24: Forms the conical core that
CC       encapsulates the genomic RNA-nucleocapsid complex in the virion.
CC       Most core are conical, with only 7% tubular. The core is
CC       constituted by capsid protein hexamer subunits. The core is
CC       disassembled soon after virion entry (By similarity). Host
CC       restriction factors such as TRIM5-alpha or TRIMCyp bind retroviral
CC       capsids and cause premature capsid disassembly, leading to blocks
```

```
CC       in reverse transcription. Capsid restriction by TRIM5 is one of
CC       the factors which restricts HIV-1 to the human species. Host PIN1
CC       apparently facilitates the virion uncoating. On the other hand,
CC       interactions with PDZD8 or CYPA stabilize the capsid.
CC       {ECO:0000250|UniProtKB:P04585, ECO:0000250|UniProtKB:P12497}.
CC   -!- FUNCTION: Nucleocapsid protein p7: Encapsulates and protects viral
CC       dimeric unspliced genomic RNA (gRNA). Binds these RNAs through its
CC       zinc fingers. Acts as a nucleic acid chaperone which is involved
CC       in rearangement of nucleic acid secondary structure during gRNA
CC       retrotranscription. Also facilitates template switch leading to
CC       recombination. As part of the polyprotein, participates in gRNA
CC       dimerization, packaging, tRNA incorporation and virion assembly.
CC       {ECO:0000250|UniProtKB:P04585}.
CC   -!- FUNCTION: Protease: Aspartyl protease that mediates proteolytic
CC       cleavages of Gag and Gag-Pol polyproteins during or shortly after
CC       the release of the virion from the plasma membrane. Cleavages take
CC       place as an ordered, step-wise cascade to yield mature proteins.
CC       This process is called maturation. Displays maximal activity
CC       during the budding process just prior to particle release from the
CC       cell. Also cleaves Nef and Vif, probably concomitantly with viral
CC       structural proteins on maturation of virus particles. Hydrolyzes
CC       host EIF4GI and PABP1 in order to shut off the capped cellular
CC       mRNA translation. The resulting inhibition of cellular protein
CC       synthesis serves to ensure maximal viral gene expression and to
CC       evade host immune response (By similarity).
CC       {ECO:0000250|UniProtKB:P04585, ECO:0000255|PROSITE-
CC       ProRule:PRU00275}.
CC   -!- FUNCTION: Reverse transcriptase/ribonuclease H: Multifunctional
CC       enzyme that converts the viral RNA genome into dsDNA in the
CC       cytoplasm, shortly after virus entry into the cell. This enzyme
CC       displays a DNA polymerase activity that can copy either DNA or RNA
CC       templates, and a ribonuclease H (RNase H) activity that cleaves
CC       the RNA strand of RNA-DNA heteroduplexes in a partially processive
CC       3' to 5' endonucleasic mode. Conversion of viral genomic RNA into
CC       dsDNA requires many steps. A tRNA(3)-Lys binds to the primer-
CC       binding site (PBS) situated at the 5'-end of the viral RNA. RT
CC       uses the 3' end of the tRNA primer to perform a short round of
CC       RNA-dependent minus-strand DNA synthesis. The reading proceeds
CC       through the U5 region and ends after the repeated (R) region which
CC       is present at both ends of viral RNA. The portion of the RNA-DNA
CC       heteroduplex is digested by the RNase H, resulting in a ssDNA
CC       product attached to the tRNA primer. This ssDNA/tRNA hybridizes
CC       with the identical R region situated at the 3' end of viral RNA.
CC       This template exchange, known as minus-strand DNA strong stop
CC       transfer, can be either intra- or intermolecular. RT uses the 3'
CC       end of this newly synthesized short ssDNA to perform the RNA-
CC       dependent minus-strand DNA synthesis of the whole template. RNase
CC       H digests the RNA template except for two polypurine tracts (PPTs)
CC       situated at the 5'-end and near the center of the genome. It is
CC       not clear if both polymerase and RNase H activities are
CC       simultaneous. RNase H probably can proceed both in a polymerase-
CC       dependent (RNA cut into small fragments by the same RT performing
CC       DNA synthesis) and a polymerase-independent mode (cleavage of
CC       remaining RNA fragments by free RTs). Secondly, RT performs DNA-
CC       directed plus-strand DNA synthesis using the PPTs that have not
CC       been removed by RNase H as primers. PPTs and tRNA primers are then
CC       removed by RNase H. The 3' and 5' ssDNA PBS regions hybridize to
CC       form a circular dsDNA intermediate. Strand displacement synthesis
CC       by RT to the PBS and PPT ends produces a blunt ended, linear dsDNA
CC       copy of the viral genome that includes long terminal repeats
CC       (LTRs) at both ends. {ECO:0000250|UniProtKB:P04585}.
CC   -!- FUNCTION: Integrase: Catalyzes viral DNA integration into the host
CC       chromosome, by performing a series of DNA cutting and joining
CC       reactions. This enzyme activity takes place after virion entry
CC       into a cell and reverse transcription of the RNA genome in dsDNA.
CC       The first step in the integration process is 3' processing. This
CC       step requires a complex comprising the viral genome, matrix
CC       protein, Vpr and integrase. This complex is called the pre-
CC       integration complex (PIC). The integrase protein removes 2
CC       nucleotides from each 3' end of the viral DNA, leaving recessed CA
CC       OH's at the 3' ends. In the second step, the PIC enters cell
CC       nucleus. This process is mediated through integrase and Vpr
CC       proteins, and allows the virus to infect a non dividing cell. This
CC       ability to enter the nucleus is specific of lentiviruses, other
CC       retroviruses cannot and rely on cell division to access cell
CC       chromosomes. In the third step, termed strand transfer, the
CC       integrase protein joins the previously processed 3' ends to the 5'
CC       ends of strands of target cellular DNA at the site of integration.
CC       The 5'-ends are produced by integrase-catalyzed staggered cuts, 5
CC       bp apart. A Y-shaped, gapped, recombination intermediate results,
CC       with the 5'-ends of the viral DNA strands and the 3' ends of
CC       target DNA strands remaining unjoined, flanking a gap of 5 bp. The
CC       last step is viral DNA integration into host chromosome. This
CC       involves host DNA repair synthesis in which the 5 bp gaps between
CC       the unjoined strands are filled in and then ligated. Since this
CC       process occurs at both cuts flanking the HIV genome, a 5 bp
CC       duplication of host DNA is produced at the ends of HIV-1
CC       integration. Alternatively, Integrase may catalyze the excision of
CC       viral DNA just after strand transfer, this is termed
CC       disintegration. {ECO:0000250|UniProtKB:P04585}.
CC   -!- CATALYTIC ACTIVITY: Specific for a P1 residue that is hydrophobic,
CC       and P1' variable, but often Pro. {ECO:0000255|PROSITE-
CC       ProRule:PRU00275}.
CC   -!- CATALYTIC ACTIVITY: Endohydrolysis of RNA in RNA/DNA hybrids.
CC       Three different cleavage modes: 1. sequence-specific internal
CC       cleavage of RNA. Human immunodeficiency virus type 1 and Moloney
CC       murine leukemia virus enzymes prefer to cleave the RNA strand one
CC       nucleotide away from the RNA-DNA junction. 2. RNA 5'-end directed
CC       cleavage 13-19 nucleotides from the RNA end. 3. DNA 3'-end
CC       directed cleavage 15-20 nucleotides away from the primer terminus.
CC       {ECO:0000250}.
CC   -!- CATALYTIC ACTIVITY: 3'-end directed exonucleolytic cleavage of
CC       viral RNA-DNA hybrid. {ECO:0000250}.
CC   -!- CATALYTIC ACTIVITY: Deoxynucleoside triphosphate + DNA(n) =
```

```
CC       diphosphate + DNA(n+1). {ECO:0000255|PROSITE-ProRule:PRU00405}.
CC   -!- COFACTOR:
CC       Name=Mg(2+); Xref=ChEBI:CHEBI:18420; Evidence={ECO:0000250};
CC       Note=Binds 2 magnesium ions for reverse transcriptase polymerase
CC       activity. {ECO:0000250};
CC   -!- COFACTOR:
CC       Name=Mg(2+); Xref=ChEBI:CHEBI:18420; Evidence={ECO:0000250};
CC       Note=Binds 2 magnesium ions for ribonuclease H (RNase H) activity.
CC       Substrate-binding is a precondition for magnesium binding.
CC       {ECO:0000250};
CC   -!- COFACTOR:
CC       Name=Mg(2+); Xref=ChEBI:CHEBI:18420; Evidence={ECO:0000250};
CC       Note=Magnesium ions are required for integrase activity. Binds at
CC       least 1, maybe 2 magnesium ions. {ECO:0000250};
CC   -!- ENZYME REGULATION: Protease: The viral protease is inhibited by
CC       many synthetic protease inhibitors (PIs), such as amprenavir,
CC       atazanavir, indinavir, loprinavir, nelfinavir, ritonavir and
CC       saquinavir. Use of protease inhibitors in tritherapy regimens
CC       permit more ambitious therapeutic strategies. Reverse
CC       transcriptase/ribonuclease H: RT can be inhibited either by
CC       nucleoside RT inhibitors (NRTIs) or by non nucleoside RT
CC       inhibitors (NNRTIs). NRTIs act as chain terminators, whereas
CC       NNRTIs inhibit DNA polymerization by binding a small hydrophobic
CC       pocket near the RT active site and inducing an allosteric change
CC       in this region. Classical NRTIs are abacavir, adefovir (PMEA),
CC       didanosine (ddI), lamivudine (3TC), stavudine (d4T), tenofovir
CC       (PMPA), zalcitabine (ddC), and zidovudine (AZT). Classical NNRTIs
CC       are atevirdine (BHAP U-87201E), delavirdine, efavirenz (DMP-266),
CC       emivirine (I-EBU), and nevirapine (BI-RG-587). The tritherapies
CC       used as a basic effective treatment of AIDS associate two NRTIs
CC       and one NNRTI. {ECO:0000250}.
CC   -!- SUBUNIT: Matrix protein p17: Homotrimer; further assembles as
CC       hexamers of trimers (By similarity). Matrix protein p17: Interacts
CC       with gp41 (via C-terminus) (By similarity). Matrix protein p17:
CC       interacts with host CALM1; this interaction induces a
CC       conformational change in the Matrix protein, triggering exposure
CC       of the myristate group (By similarity). Matrix protein p17:
CC       interacts with host AP3D1; this interaction allows the polyprotein
CC       trafficking to multivesicular bodies during virus assembly (By
CC       similarity). Matrix protein p17: Part of the pre-integration
CC       complex (PIC) which is composed of viral genome, matrix protein,
CC       Vpr and integrase (By similarity). Capsid protein p24: Homodimer;
CC       the homodimer further multimerizes as homohexamers or
CC       homopentamers. Capsid protein p24: Interacts with human PPIA/CYPA
CC       (By similarity); This interaction stabilizes the capsid. Capsid
CC       protein p24: Interacts with human NUP153 (By similarity). Capsid
CC       protein p24: Interacts with host PDZD8; this interaction
CC       stabilizes the capsid (By similarity). Capsid protein p24:
CC       Interacts with monkey TRIM5; this interaction destabilizes the
CC       capsid (By similarity).Protease: Homodimer, whose active site
CC       consists of two apposed aspartic acid residues. Reverse
CC       transcriptase/ribonuclease H: Heterodimer of p66 RT and p51 RT (RT
CC       p66/p51). Heterodimerization of RT is essential for DNA polymerase
CC       activity. Despite the sequence identities, p66 RT and p51 RT have
CC       distinct folding. Integrase: Homodimer; possibly can form
CC       homotetramer. Integrase: Part of the pre-integration complex (PIC)
CC       which is composed of viral genome, matrix protein, Vpr and
CC       integrase. Integrase: Interacts with human SMARCB1/INI1 and human
CC       PSIP1/LEDGF isoform 1. Integrase: Interacts with human KPNA3; this
CC       interaction might play a role in nuclear import of the pre-
CC       integration complex (By similarity). Integrase: Interacts with
CC       human NUP153; this interaction might play a role in nuclear import
CC       of the pre-integration complex (By similarity).
CC       {ECO:0000250|UniProtKB:P04585, ECO:0000250|UniProtKB:P12497}.
CC   -!- SUBCELLULAR LOCATION: Gag-Pol polyprotein: Host cell membrane;
CC       Lipid-anchor. Host endosome, host multivesicular body. Note=These
CC       locations are linked to virus assembly sites. The main location is
CC       the cell membrane, but under some circumstances, late endosomal
CC       compartments can serve as productive sites for virion assembly.
CC       {ECO:0000250|UniProtKB:P12497}.
CC   -!- SUBCELLULAR LOCATION: Matrix protein p17: Virion membrane; Lipid-
CC       anchor {ECO:0000305}. Host nucleus {ECO:0000250}. Host cytoplasm
CC       {ECO:0000250}.
CC   -!- SUBCELLULAR LOCATION: Capsid protein p24: Virion {ECO:0000305}.
CC   -!- SUBCELLULAR LOCATION: Nucleocapsid protein p7: Virion
CC       {ECO:0000305}.
CC   -!- SUBCELLULAR LOCATION: Reverse transcriptase/ribonuclease H: Virion
CC       {ECO:0000305}.
CC   -!- SUBCELLULAR LOCATION: Integrase: Virion {ECO:0000305}. Host
CC       nucleus {ECO:0000305}. Host cytoplasm {ECO:0000305}. Note=Nuclear
CC       at initial phase, cytoplasmic at assembly. {ECO:0000305}.
CC   -!- ALTERNATIVE PRODUCTS:
CC       Event=Ribosomal frameshifting; Named isoforms=2;
CC         Comment=Translation results in the formation of the Gag
CC         polyprotein most of the time. Ribosomal frameshifting at the
CC         gag-pol genes boundary occurs at low frequency and produces the
CC         Gag-Pol polyprotein. This strategy of translation probably
CC         allows the virus to modulate the quantity of each viral protein.
CC         Maintenance of a correct Gag to Gag-Pol ratio is essential for
CC         RNA dimerization and viral infectivity.;
CC       Name=Gag-Pol polyprotein;
CC         IsoId=Q9WC54-1; Sequence=Displayed;
CC         Note=Produced by -1 ribosomal frameshifting.;
CC       Name=Gag polyprotein;
CC         IsoId=Q9WC53-1; Sequence=External;
CC         Note=Produced by conventional translation.;
CC   -!- DOMAIN: Reverse transcriptase/ribonuclease H: RT is structured in
CC       five subdomains: finger, palm, thumb, connection and RNase H.
CC       Within the palm subdomain, the 'primer grip' region is thought to
CC       be involved in the positioning of the primer terminus for
CC       accommodating the incoming nucleotide. The RNase H domain
CC       stabilizes the association of RT with primer-template.
CC       {ECO:0000250}.
CC   -!- DOMAIN: Reverse transcriptase/ribonuclease H: The tryptophan
```

```
CC       repeat motif is involved in RT p66/p51 dimerization (By
CC       similarity). {ECO:0000250}.
CC   -!- DOMAIN: Integrase: The core domain contains the D-x(n)-D-x(35)-E
CC       motif, named for the phylogenetically conserved glutamic acid and
CC       aspartic acid residues and the invariant 35 amino acid spacing
CC       between the second and third acidic residues. Each acidic residue
CC       of the D,D(35)E motif is independently essential for the 3'-
CC       processing and strand transfer activities of purified integrase
CC       protein. {ECO:0000250}.
CC   -!- PTM: Gag-Pol polyprotein: Specific enzymatic cleavages by the
CC       viral protease yield mature proteins. The protease is released by
CC       autocatalytic cleavage. The polyprotein is cleaved during and
CC       after budding, this process is termed maturation. Proteolytic
CC       cleavage of p66 RT removes the RNase H domain to yield the p51 RT
CC       subunit. Nucleocapsid protein p7 might be further cleaved after
CC       virus entry. {ECO:0000250|UniProtKB:P04585, ECO:0000255|PROSITE-
CC       ProRule:PRU00405}.
CC   -!- PTM: Matrix protein p17: Tyrosine phosphorylated presumably in the
CC       virion by a host kinase. Phosphorylation is apparently not a major
CC       regulator of membrane association. {ECO:0000250|UniProtKB:P04585}.
CC   -!- PTM: Capsid protein p24: Phosphorylated possibly by host MAPK1;
CC       this phosphorylation is necessary for Pin1-mediated virion
CC       uncoating. {ECO:0000250|UniProtKB:P12493}.
CC   -!- PTM: Nucleocapsid protein p7: Methylated by host PRMT6, impairing
CC       its function by reducing RNA annealing and the initiation of
CC       reverse transcription. {ECO:0000250|UniProtKB:P03347}.
CC   -!- MISCELLANEOUS: Reverse transcriptase/ribonuclease H: Error-prone
CC       enzyme that lacks a proof-reading function. High mutations rate is
CC       a direct consequence of this characteristic. RT also displays
CC       frequent template switching leading to high recombination rate.
CC       Recombination mostly occurs between homologous regions of the two
CC       copackaged RNA genomes. If these two RNA molecules derive from
CC       different viral strains, reverse transcription will give rise to
CC       highly recombinated proviral DNAs. {ECO:0000250}.
CC   -!- MISCELLANEOUS: HIV-1 lineages are divided in three main groups, M
CC       (for Major), O (for Outlier), and N (for New, or Non-M, Non-O).
CC       The vast majority of strains found worldwide belong to the group
CC       M. Group O seems to be endemic to and largely confined to Cameroon
CC       and neighboring countries in West Central Africa, where these
CC       viruses represent a small minority of HIV-1 strains. The group N
CC       is represented by a limited number of isolates from Cameroonian
CC       persons. The group M is further subdivided in 9 clades or subtypes
CC       (A to D, F to H, J and K).
CC   -!- MISCELLANEOUS: Resistance to inhibitors associated with mutations
CC       are observed both in viral protease and in reverse transcriptase.
CC       Most of the time, single mutations confer only a modest reduction
CC       in drug susceptibility. Combination of several mutations is
CC       usually required to develop a high-level drug resistance. These
CC       mutations are predominantly found in clade B viruses and not in
CC       other genotypes. They are listed in the clade B representative
CC       isolate HXB2 (AC P04585).
CC   -!- WEB RESOURCE: Name=HIV drug resistance mutations;
CC       URL="https://www.iasusa.org/content/hiv-drug-resistance-mutations";
CC   -!- WEB RESOURCE: Name=hivdb; Note=HIV drug resistance database;
CC       URL="http://hivdb.stanford.edu";
CC   -!- WEB RESOURCE: Name=BioAfrica: HIV bioinformatics in Africa;
CC       URL="http://www.bioafrica.net/index.html";
DR   EMBL; AF082394; AAD17757.1; ALT_SEQ; Genomic_DNA.
DR   ProteinModelPortal; Q9WC54; -.
DR   SMR; Q9WC54; -.
DR   PRO; PR:Q9WC54; -.
DR   GO; GO:0042025; C:host cell nucleus; IEA:UniProtKB-SubCell.
DR   GO; GO:0020002; C:host cell plasma membrane; IEA:UniProtKB-SubCell.
DR   GO; GO:0072494; C:host multivesicular body; IEA:UniProtKB-SubCell.
DR   GO; GO:0019013; C:viral nucleocapsid; IEA:UniProtKB-KW.
DR   GO; GO:0055036; C:virion membrane; IEA:UniProtKB-SubCell.
DR   GO; GO:0004190; F:aspartic-type endopeptidase activity; IEA:UniProtKB-KW.
DR   GO; GO:0003677; F:DNA binding; IEA:UniProtKB-KW.
DR   GO; GO:0003887; F:DNA-directed DNA polymerase activity; IEA:UniProtKB-KW.
DR   GO; GO:0004533; F:exoribonuclease H activity; IEA:UniProtKB-EC.
DR   GO; GO:0008289; F:lipid binding; IEA:UniProtKB-KW.
DR   GO; GO:0003723; F:RNA binding; IEA:UniProtKB-KW.
DR   GO; GO:0003964; F:RNA-directed DNA polymerase activity; IEA:UniProtKB-KW.
DR   GO; GO:0004523; F:RNA-DNA hybrid ribonuclease activity; IEA:InterPro.
DR   GO; GO:0005198; F:structural molecule activity; IEA:InterPro.
DR   GO; GO:0008270; F:zinc ion binding; IEA:InterPro.
DR   GO; GO:0015074; P:DNA integration; IEA:UniProtKB-KW.
DR   GO; GO:0006310; P:DNA recombination; IEA:UniProtKB-KW.
DR   GO; GO:0075713; P:establishment of integrated proviral latency; IEA:UniProtKB-KW.
DR   GO; GO:0039651; P:induction by virus of host cysteine-type endopeptidase activity involved in apoptotic process; IEA:UniProtKB-KW
DR   GO; GO:0039657; P:suppression by virus of host gene expression; IEA:UniProtKB-KW.
DR   GO; GO:0046718; P:viral entry into host cell; IEA:UniProtKB-KW.
DR   GO; GO:0044826; P:viral genome integration into host DNA; IEA:UniProtKB-KW.
DR   GO; GO:0075732; P:viral penetration into host nucleus; IEA:UniProtKB-KW.
DR   CDD; cd05482; HIV_retropepsin_like; 1.
DR   Gene3D; 1.10.10.200; -; 1.
DR   Gene3D; 1.10.1200.30; -; 1.
DR   Gene3D; 1.10.150.90; -; 1.
DR   Gene3D; 1.10.375.10; -; 1.
DR   Gene3D; 2.30.30.10; -; 1.
DR   Gene3D; 2.40.70.10; -; 1.
DR   Gene3D; 3.30.420.10; -; 2.
DR   InterPro; IPR001969; Aspartic_peptidase_AS.
DR   InterPro; IPR000721; Gag_p24.
DR   InterPro; IPR017856; Integrase-like_N.
DR   InterPro; IPR036862; Integrase_C_dom_sf_retrovir.
DR   InterPro; IPR001037; Integrase_C_retrovir.
DR   InterPro; IPR001584; Integrase_cat-core.
DR   InterPro; IPR003308; Integrase_Zn-bd_dom_N.
DR   InterPro; IPR000071; Lentvrl_matrix_N.
DR   InterPro; IPR012344; Matrix_HIV/RSV_N.
DR   InterPro; IPR001995; Peptidase_A2_cat.
DR   InterPro; IPR021109; Peptidase_aspartic_dom_sf.
DR   InterPro; IPR034170; Retropepsin-like_cat_dom.
```

```
DR   InterPro; IPR018061; Retropepsins.
DR   InterPro; IPR008916; Retrov_capsid_C.
DR   InterPro; IPR008919; Retrov_capsid_N.
DR   InterPro; IPR010999; Retrovr_matrix.
DR   InterPro; IPR012337; RNaseH-like_sf.
DR   InterPro; IPR002156; RNaseH_domain.
DR   InterPro; IPR036397; RNaseH_sf.
DR   InterPro; IPR000477; RT_dom.
DR   InterPro; IPR010659; RVT_connect.
DR   InterPro; IPR010661; RVT_thumb.
DR   InterPro; IPR001878; Znf_CCHC.
DR   InterPro; IPR036875; Znf_CCHC_sf.
DR   Pfam; PF00540; Gag_p17; 1.
DR   Pfam; PF00607; Gag_p24; 1.
DR   Pfam; PF00552; IN_DBD_C; 1.
DR   Pfam; PF02022; Integrase_Zn; 1.
DR   Pfam; PF00075; RNase_H; 1.
DR   Pfam; PF00665; rve; 1.
DR   Pfam; PF00077; RVP; 1.
DR   Pfam; PF00078; RVT_1; 1.
DR   Pfam; PF06815; RVT_connect; 1.
DR   Pfam; PF06817; RVT_thumb; 1.
DR   Pfam; PF00098; zf-CCHC; 2.
DR   PRINTS; PR00234; HIV1MATRIX.
DR   SMART; SM00343; ZnF_C2HC; 2.
DR   SUPFAM; SSF46919; SSF46919; 1.
DR   SUPFAM; SSF47836; SSF47836; 1.
DR   SUPFAM; SSF47943; SSF47943; 1.
DR   SUPFAM; SSF50122; SSF50122; 1.
DR   SUPFAM; SSF50630; SSF50630; 1.
DR   SUPFAM; SSF53098; SSF53098; 2.
DR   SUPFAM; SSF57756; SSF57756; 1.
DR   PROSITE; PS50175; ASP_PROT_RETROV; 1.
DR   PROSITE; PS00141; ASP_PROTEASE; 1.
DR   PROSITE; PS50994; INTEGRASE; 1.
DR   PROSITE; PS51027; INTEGRASE_DBD; 1.
DR   PROSITE; PS50879; RNASE_H; 1.
DR   PROSITE; PS50878; RT_POL; 1.
DR   PROSITE; PS50158; ZF_CCHC; 2.
DR   PROSITE; PS50876; ZF_INTEGRASE; 1.
PE   3: Inferred from homology;
KW   Activation of host caspases by virus; AIDS; Aspartyl protease;
KW   Capsid protein; DNA integration; DNA recombination; DNA-binding;
KW   DNA-directed DNA polymerase; Endonuclease;
KW   Eukaryotic host gene expression shutoff by virus;
KW   Eukaryotic host translation shutoff by virus; Host cell membrane;
KW   Host cytoplasm; Host endosome; Host gene expression shutoff by virus;
KW   Host membrane; Host nucleus; Host-virus interaction; Hydrolase;
KW   Lipid-binding; Lipoprotein; Magnesium; Membrane; Metal-binding;
KW   Modulation of host cell apoptosis by virus; Multifunctional enzyme;
KW   Myristate; Nuclease; Nucleotidyltransferase; Phosphoprotein; Protease;
KW   Repeat; Ribosomal frameshifting; RNA-binding;
KW   RNA-directed DNA polymerase; Transferase; Viral genome integration;
KW   Viral nucleoprotein; Viral penetration into host nucleus;
KW   Viral release from host cell; Virion; Virion maturation;
KW   Virus entry into host cell; Zinc; Zinc-finger.
FT   INIT_MET      1      1       Removed; by host. {ECO:0000250}.
FT   CHAIN         2   1432       Gag-Pol polyprotein.
FT                                /FTId=PRO_0000261281.
FT   CHAIN         2    132       Matrix protein p17. {ECO:0000250}.
FT                                /FTId=PRO_0000246556.
FT   CHAIN       133    363       Capsid protein p24. {ECO:0000250}.
FT                                /FTId=PRO_0000246557.
FT   PEPTIDE     364    376       Spacer peptide 1. {ECO:0000250}.
FT                                /FTId=PRO_0000246558.
FT   CHAIN       377    431       Nucleocapsid protein p7. {ECO:0000250}.
FT                                /FTId=PRO_0000246559.
FT   PEPTIDE     432    439       Transframe peptide. {ECO:0000255}.
FT                                /FTId=PRO_0000246731.
FT   CHAIN       440    485       p6-pol. {ECO:0000255}.
FT                                /FTId=PRO_0000246560.
FT   CHAIN       486    584       Protease. {ECO:0000250}.
FT                                /FTId=PRO_0000246561.
FT   CHAIN       585   1144       Reverse transcriptase/ribonuclease H.
FT                                {ECO:0000250}.
FT                                /FTId=PRO_0000246562.
FT   CHAIN       585   1024       p51 RT. {ECO:0000250}.
FT                                /FTId=PRO_0000246563.
FT   CHAIN      1025   1144       p15. {ECO:0000250}.
FT                                /FTId=PRO_0000246564.
FT   CHAIN      1145   1432       Integrase. {ECO:0000250}.
FT                                /FTId=PRO_0000246565.
FT   DOMAIN      505    574       Peptidase A2. {ECO:0000255|PROSITE-
FT                                ProRule:PRU00275}.
FT   DOMAIN      628    818       Reverse transcriptase.
FT                                {ECO:0000255|PROSITE-ProRule:PRU00405}.
FT   DOMAIN     1018   1141       RNase H. {ECO:0000255|PROSITE-
FT                                ProRule:PRU00408}.
FT   DOMAIN     1198   1348       Integrase catalytic.
FT                                {ECO:0000255|PROSITE-ProRule:PRU00457}.
FT   ZN_FING     389    406       CCHC-type 1. {ECO:0000255|PROSITE-
FT                                ProRule:PRU00047}.
FT   ZN_FING     410    427       CCHC-type 2. {ECO:0000255|PROSITE-
FT                                ProRule:PRU00047}.
FT   ZN_FING    1147   1188       Integrase-type. {ECO:0000255|PROSITE-
FT                                ProRule:PRU00450}.
FT   DNA_BIND   1367   1414       Integrase-type. {ECO:0000255|PROSITE-
FT                                ProRule:PRU00506}.
FT   REGION        7     31       Interaction with Gp41.
FT                                {ECO:0000250|UniProtKB:P12497}.
FT   REGION        8     43       Interaction with host CALM1.
FT                                {ECO:0000250|UniProtKB:P04585}.
FT   REGION       12     19       Interaction with host AP3D1.
FT                                {ECO:0000250|UniProtKB:P12497}.
```

```
FT   REGION       14     33       Interaction with membrane
FT                                phosphatidylinositol 4,5-bisphosphate and
FT                                RNA. {ECO:0000250|UniProtKB:P12497}.
FT   REGION       73     77       Interaction with membrane
FT                                phosphatidylinositol 4,5-bisphosphate.
FT                                {ECO:0000250|UniProtKB:P12497}.
FT   REGION      189    227       Interaction with human PPIA/CYPA and
FT                                NUP153. {ECO:0000250|UniProtKB:P12497}.
FT   REGION      277    363       Dimerization/Multimerization of capsid
FT                                protein p24.
FT                                {ECO:0000250|UniProtKB:P04585}.
FT   REGION      486    490       Dimerization of protease.
FT                                {ECO:0000250|UniProtKB:P04585}.
FT   REGION      534    540       Dimerization of protease.
FT                                {ECO:0000250|UniProtKB:P04585}.
FT   REGION      573    585       Dimerization of protease.
FT                                {ECO:0000250|UniProtKB:P04585}.
FT   REGION      811    819       RT 'primer grip'. {ECO:0000250}.
FT   MOTIF        16     22       Nuclear export signal. {ECO:0000250}.
FT   MOTIF        26     32       Nuclear localization signal.
FT                                {ECO:0000250}.
FT   MOTIF       982    998       Tryptophan repeat motif. {ECO:0000250}.
FT   ACT_SITE    510    510       For protease activity; shared with
FT                                dimeric partner. {ECO:0000255|PROSITE-
FT                                ProRule:PRU10094}.
FT   METAL       694    694       Magnesium; catalytic; for reverse
FT                                transcriptase activity. {ECO:0000250}.
FT   METAL       769    769       Magnesium; catalytic; for reverse
FT                                transcriptase activity. {ECO:0000250}.
FT   METAL       770    770       Magnesium; catalytic; for reverse
FT                                transcriptase activity. {ECO:0000250}.
FT   METAL      1027   1027       Magnesium; catalytic; for RNase H
FT                                activity. {ECO:0000250}.
FT   METAL      1062   1062       Magnesium; catalytic; for RNase H
FT                                activity. {ECO:0000250}.
FT   METAL      1082   1082       Magnesium; catalytic; for RNase H
FT                                activity. {ECO:0000250}.
FT   METAL      1133   1133       Magnesium; catalytic; for RNase H
FT                                activity. {ECO:0000250}.
FT   METAL      1208   1208       Magnesium; catalytic; for integrase
FT                                activity. {ECO:0000250}.
FT   METAL      1260   1260       Magnesium; catalytic; for integrase
FT                                activity. {ECO:0000250}.
FT   METAL      1296   1296       Magnesium; catalytic; for integrase
FT                                activity. {ECO:0000250|UniProtKB:P04585}.
FT   SITE        132    133       Cleavage; by viral protease.
FT                                {ECO:0000250}.
FT   SITE        221    222       Cis/trans isomerization of proline
FT                                peptide bond; by human PPIA/CYPA.
FT                                {ECO:0000250}.
FT   SITE        363    364       Cleavage; by viral protease.
FT                                {ECO:0000250}.
FT   SITE        376    377       Cleavage; by viral protease.
FT                                {ECO:0000250}.
FT   SITE        431    432       Cleavage; by viral protease.
FT                                {ECO:0000255}.
FT   SITE        439    440       Cleavage; by viral protease.
FT                                {ECO:0000250}.
FT   SITE        485    486       Cleavage; by viral protease.
FT                                {ECO:0000250}.
FT   SITE        584    585       Cleavage; by viral protease.
FT                                {ECO:0000250}.
FT   SITE        985    985       Essential for RT p66/p51
FT                                heterodimerization. {ECO:0000250}.
FT   SITE        998    998       Essential for RT p66/p51
FT                                heterodimerization. {ECO:0000250}.
FT   SITE       1024   1025       Cleavage; by viral protease; partial.
FT                                {ECO:0000250}.
FT   SITE       1144   1145       Cleavage; by viral protease.
FT                                {ECO:0000250}.
FT   MOD_RES     132    132       Phosphotyrosine; by host. {ECO:0000250}.
FT   LIPID         2      2       N-myristoyl glycine; by host.
FT                                {ECO:0000250}.
SQ   SEQUENCE   1432 AA;  162130 MW;  B0A04035D51B38A3 CRC64;
     MGARASILSG GKLDDWEKIR LRPGGKKKYR IKHLVWASRE LDRFALNPGL LESAKGCQQI
     LVQLQPALQT GTQEIKSLYN TVATLYCVHQ RIEIKDTMEA LEKIEEIQNK NKQQAQKAET
     DKKDNSQVSQ NYPIVQNLQG QPVHQALSPR TLNAWVKVIE EKAFSPEVIP MFSALSEGAT
     PQDLNTMLNT IGGHQAAMQM LKDTINEEAA EWDRVHPVHA GPIAPGQVRE PRGSDIAGTT
     STLQEQIGWM TGNPPIPVGE IYKRWIILGL NKIVRMYSPV SILDIRQGPK EPFRDYVDRF
     FKALRAEQAT QDVKNWMTDT LLVQNANPDC KTILKALGSG ATLEEMMTAC QGVGGPGHKA
     RVLAEAMSQV TNTNIMMQRG NFRDHKRIVK CFNCGKQGHI AKNCRAPRKK GCWKCGKEGH
     QMKDCTERQA NFFREDLAFQ QREARELSPE QTRANSPTSR EPRARRGDPL PETGAEGQGT
     VSSNFPQITL WQRPLVTIRI GGQLREALLD TGADDTVLED IDLPRKWKPK MIGGIGGFIK
     VRQYNEVPIE IEGKKAIGTV LIGPTPVNII GRNMLTQLGC TLNFPISPIE TVPVKLKPGM
     DGPKIKQWPL TEEKIKALTQ ICAEMEEEGK ISRVGPENPY NTPVFAIKKK DSTKWRKLVD
     FRELNKRTQD FWEVQLGIPH PAGLKKKKSV TVLDVGDAYF SVPLYEDFRK YTAFTIPSIN
     NETPGIRYQY NVLPQGWKGS PAIFQCSMTK ILKPFRERNP EIVIYQYMDD LYVGSDLEIE
     QHRRKIKELR EHLLKWGFTT PDKKHQKEPP FLWMGYELHP DKWTVQPIQL PEKEDWTVND
     IQKLVGKLNW ASQIYPGIKV KQLCKLLKGA KALTDIVPLT REAELELAEN KEILKEPVHG
     VYYDSAKELI AEVQKQGLDQ WTYQIYQEPF KNLKTGKYAK RRSAHTNDVK QLAEVVQKIA
     LEAIVIWGKT PKFRLPIQRE TWETWWTDYW QATWIPEWEF VNTPPLVKLW YQLEKEPIMG
     AETFYVDGAS NRETKTGKAG YVTDKGRQKV VTLTDTTNQK TELHAIYLAL RDSGLEVNIV
     TDSQYALGII QAQPDKSESE LVNQIIEELI KKEKVYLSWV PAHKGIGGNE QVDKLVSSGI
     RKVLFLDGID KAQEDHEKYH SNWRAMASDF NLPPVVAKEI VASCDKCQLK GEAMHGQVDC
     SPGIWQLDCT HLEGKVILVA VHVASGYIEA EVIPAETGQE AAFFILKLAG RWPVKVIHTD
     NGSNFTSGAV KAACWWADIK QEFGIPYNPQ SQGVVESMNK ELKKIIGQVR EQAEHLKTAV
     QMAVFIHNFK RKGGIGGYSA GERIIDIIAT DIQTRELQKQ ITKIQNFRVY YRDSRDPIWK
     GPAKLPWKGE GAVVIQDNSE IKVVPRRKAK IIRDYGKQMA GDDCVAGRQD ED
```

Mascot: http://www.matrixscience.com/

MATRIX SCIENCE MASCOT Search Results

Protein View: DARB_BPP1

Defense against restriction protein B OS=Enterobacteria phage P1 GN=darB PE=3 SV=1

**Database:**	SwissProt
**Score:**	162
**Expect:**	1e-12
**Monoisotopic mass (M_r):**	251899
**Calculated pI:**	5.41
**Taxonomy:**	Escherichia virus P1

Sequence similarity is available as an NCBI BLAST search of DARB_BPP1 against nr.

Search parameters

**Enzyme:**	Trypsin: cuts C-term side of KR unless next residue is P.
**Fixed modifications:**	Carbamidomethyl (C)
**Variable modifications:**	Acetyl (N-term), Oxidation (M)
**Mass values searched:**	31
**Mass values matched:**	30

Protein sequence coverage: 13%

Matched peptides shown in ***bold red***.

1	MNK**LSMGVFR**	CSSVSEILKY	IRAITSHRAP	IKYGVEKVEG	K**SYDRLR**REA
51	NQKAIDLLNS	LVDGATLTDE	QRQILAGYTG	EGGIGGSVSE	YYTPKPIAEG
101	VWEIMKLYGA	DVGNTLEPSA	GTGVFNETKP	VGTVMTATEI	SSVSGRINQL
151	LHPEDSVQIS	PFEQLAVSTP	NDSFDHVVGN	VPFGGRDNTR	**NIDKPYAEET**
201	**DMGSYFMLRM**	**LDK**IKPGGFM	CVIVPPSIVS	GSNMKRLRLR	LSRK**AEFLGA**
251	**HR**LPTGTFDA	NGTSTVVDVV	LMRK**HPAEMA**	**EK**IPLVHEST	LESANVLWPT
301	FISGKWFEKD	GR**RFVHGTQE**	**K**GFQGRIEVR	ADGQIDNQAL	KAKLIHCFES
351	RIDWYLLDMA	EPSPTADVVD	EGEMRLINGV	WQKYAGGRWI	ESDAGKELKI
401	DVASYGADSW	EALQRNLTTT	EGRLGMTFTQ	MANVRDKYTT	SISDDMVQLV
451	DWINSQPEKY	RERLYRGAMI	GRMLIEYQDM	KAAGHSAEQI	EQQRLSLVSR
501	LQAEIDRFGN	PGRGPIAK**LS**	**GSGARAWFAF**	**R**GAIKLDGTI	SDELTGKLVT
551	HDSSASYDST	SYQDTLR**YLY**	**SDLTR**DPIQL	DDFRLAFTGE	LPASDDELLN
601	LLASTPGIAV	SPYGGIVPFA	RATSGDINEI	VAPKQEFLAT	LTDGPVKNNV
651	LNQLAAIEEK	RIK**TPAENIR**	FKLNSRWFDR	SVILEFLQEN	GYPDLRYVQS
701	VQLEGDEMVS	DTYHGGDGLF	VGHRYGVVQR	KDKETGEIRY	EWDRKSGENA
751	TGFPAQLEKY	LNGAR**IGGKD**	**SATANGYR**EQ	MALLEDQFNK	WIKTHDR**YDE**
801	**LVAKYNDVFN**	**SNIPYEHSGD**	**PLGLKGLSGK**	RQPFDYQNSE	VRRLSEDGRG
851	ILGFGTGLGK	TTTALALEAF	NYENGRSTRT	AYVVPKSVLE	NWYYEAKEFL
901	SEEAFSNYLF	VGLDVLMDGD	QIRQVPVLDE	NGKPVLGTDG	TPVMRDALKL
951	ADEATITARM	NAIPHSNYR**A**	**VVFTKEQYAR**	**IPLRDDTVDE**	**HAQDMLYDFV**
1001	**AAGR**VASAMD	SDSHRKEAAR	RRVLSEYSDT	GTEKAEKYPY	FEDMGFDSVI
1051	ADEGHNYRNS	YK**NGREASQL**	**AYLPTSAVAQ**	**SAR**DMAIK**NA**	**YLMKK**NGGRG
1101	PVLLTATPVV	NTPIDAYNML	SHVLPKEYWQ	KMGIYGPDDF	VKFFGKTRLE
1151	TVQKISGEVE	EKMALVGFEN	LDALRGIFHR	WTTLKTAEDV	KDTVEIPELD
1201	EHQQDAPLTE	EQLAAYEELR	QQAEAAAKAN	NGVTTSVNED	GVIEHEKARP
1251	IFSIIRDMDR	VCTDMDLYYR	RITYRFLPEY	ADAVQQLADS	LPKQATSEDD
1301	DSDDSITQQS	QYSLIDKGEF	IQLQVPEAFE	QEVNKRLARF	GIDEQTVTHP
1351	VTPKYAKLIA	TLK**EFFPEGK**	QIIFTDEKTQ	HQKLKRIICN	ALNLEPSKVG
1401	ILNAQTVAEA	GKTGKKLKAV	KPPKELPDEP	TDAQIAKYNE	QMALYDAYIA
1451	QQNEMSLGGL	EKIAADFQEG	RTPIIICNKK	AEVGINLHRG	TTDIHHLTLP
1501	WTPASIAQRN	GRGARVGSNR	**ASVRVHYYCG**	**K**GSFDEYRLK	TLKRKAGWIS
1551	DILRSDKSEM	ENADANDMIE	MQMYTAKDDG	ERLAMMQVQM	DKAKAAQRAR
1601	QKEQATIDLQ	NYIKAQHAAG	EDVEVLTAEL	ERSKAELEKT	TAEVAKFKQA
1651	VMAK**AADNAD**	**WKAR**WGSVHH	TDR**MLLAQYR**	**ASLK**SAIQRK	ANISQAISPY
1701	EKLLNRTQKA	ATDIKRLRPL	VEDAINKGIL	DVDPDLVNHA	SEFLVIGDRS
1751	WRVGQYYDCA	GDIVRIKSLD	FDSQRADVEI	IFTFK**GTKSG**	**NWDVK**TLDKQ
1801	VDVTPDEDAV	MQKISGGVSI	AGINDIISCD	DFYRFQQRGM	IKITDSYGVQ
1851	TTESGYSIDF	VGTYTDPLK**H**	**AVYPDRR**DGA	LKSSIAKWVL	GMMSEGNNRQ
1901	VRLAEVFLTE	LFGSNYGDVI	ASYGDTLSPE	AIQEKIADAI	AR**MPEKTSQG**
1951	**ATR**NGDSELE	VTNAIFGTHE	FR**ASDYEITT**	**AQFGTIGIYS**	**NKAEIK**QAMD
2001	AASARIAAER	EANLNHAVAA	LTQSWVTAIR	EAATTGKITP	AIADVVNDGS
2051	KFMDAYKMDA	VQLPSAYGQL	SYRMTYNLVS	MFSDLAILGL	VDLNEVTPEL
2101	LSMR**KNHVEI**	**LQR**INTVLAG	RTDEEKQADA	DRINLALGNI	TEEEIAARNE

2151 KQEELSSIQG DATSIAQSLG LNYRVSTADL KMMYAPKFAA GEVFGLQEAS
2201 GMKGVLFRAK DAIKTK**FGAR WLPAK**AKNSD FPGNWWIIET KHNVADVLAV
2251 IQQYA

Unformatted sequence string: 2255 residues (for pasting into other applications).

Sort by ◉ residue number ○ increasing mass ○ decreasing mass
Show ◉ matched peptides only ○ predicted peptides also

Start – End	Observed	Mr(expt)	Mr(calc)	Delta	M	Peptide
4 – 10	825.3318	824.3245	824.4215	-0.0969	0	K.LSMGVFR.C + Oxidation (M)
42 – 47	809.3675	808.3602	808.4191	-0.0589	1	K.SYDRLR.R
191 – 213	2825.2542	2824.2469	2824.2757	-0.0288	1	R.NIDKPYAEETDMGSYFMLRMLDK.I + Acetyl (N-term); Oxidation (M)
245 – 252	900.4571	899.4499	899.4613	-0.0115	0	K.AEFLGAHR.L
275 – 282	928.5975	927.5902	927.4120	0.1782	0	K.HPAEMAEK.I + Oxidation (M)
313 – 321	1101.5757	1100.5684	1100.5727	-0.0043	1	R.RFVHGTQEK.G
519 – 531	1425.7670	1424.7597	1424.7313	0.0284	1	K.LSGSGARAWFAFR.G
568 – 575	1030.4738	1029.4665	1029.5131	-0.0466	0	R.YLYSDLTR.D
664 – 670	842.4247	841.4175	841.4294	-0.0119	0	K.TPAENIR.F + Acetyl (N-term)
766 – 778	1309.6626	1308.6553	1308.6422	0.0131	1	R.IGGKDSATANGYR.E
798 – 804	837.3813	836.3741	836.4280	-0.0539	0	R.YDELVAK.Y
805 – 830	2863.2097	2862.2024	2862.3824	-0.1800	1	K.YNDVFNSNIPYEHSGDPLGLKGLSGK.R + Acetyl (N-term)
970 – 980	1353.6761	1352.6688	1352.7088	-0.0400	1	R.AVVFTKEQYAR.I + Acetyl (N-term)
976 – 984	1145.5754	1144.5681	1144.6353	-0.0671	1	K.EQYARIPLR.D
985 – 1004	2284.0381	2283.0308	2282.9750	0.0558	0	R.DDTVDEHAQDMLYDFVAAGR.V + Oxidation (M)
1063 – 1083	2232.0989	2231.0916	2231.1294	-0.0378	1	K.NGREASQLAYLPTSAVAQSAR.D + Acetyl (N-term)
1089 – 1094	781.2904	780.2831	780.3840	-0.1008	0	K.NAYLMK.K + Acetyl (N-term)
1089 – 1095	925.4584	924.4512	924.4738	-0.0227	1	K.NAYLMKK.N + Acetyl (N-term); Oxidation (M)
1364 – 1370	853.3976	852.3904	852.4018	-0.0114	0	K.EFFPEGK.Q
1521 – 1531	1381.7407	1380.7334	1380.6608	0.0726	1	R.ASVRVHYYCGK.G + Acetyl (N-term)
1655 – 1664	1117.5670	1116.5597	1116.5312	0.0285	1	K.AADNADWKAR.W
1674 – 1684	1293.6737	1292.6664	1292.7274	-0.0610	1	R.MLLAQYRASLK.S
1786 – 1795	1091.4645	1090.4572	1090.5407	-0.0835	1	K.GTKSGNWDVK.T
1786 – 1795	1133.5444	1132.5371	1132.5513	-0.0142	1	K.GTKSGNWDVK.T + Acetyl (N-term)
1870 – 1877	1013.4809	1012.4736	1012.5202	-0.0466	1	K.HAVYPDRR.D
1943 – 1953	1205.6116	1204.6043	1204.5870	0.0173	1	R.MPEKTSQGATR.N
1943 – 1953	1221.5941	1220.5868	1220.5819	0.0049	1	R.MPEKTSQGATR.N + Oxidation (M)
1973 – 1996	2662.1765	2661.1692	2661.3173	-0.1481	1	R.ASDYEITTAQFGTIGIYSNKAEIK.Q + Acetyl (N-term)
2105 – 2113	1178.5331	1177.5258	1177.6567	-0.1309	1	R.KNHVEILQR.I + Acetyl (N-term)
2217 – 2225	1045.4888	1044.4815	1044.5869	-0.1053	1	K.FGARWLPAK.A

No match to: 1161.5974

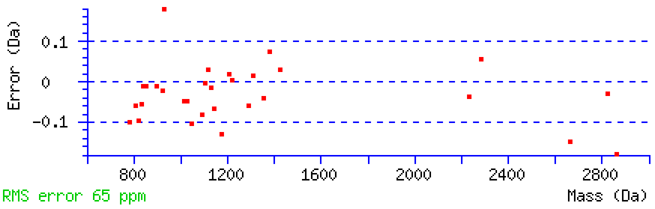

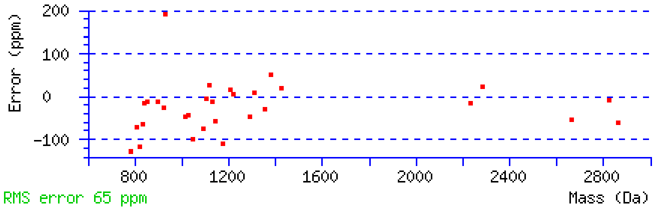


```
ID   DARB_BPP1               Reviewed;         2255 AA.
AC   Q71TF8;
DT   24-JUN-2015, integrated into UniProtKB/Swiss-Prot.
DT   05-JUL-2004, sequence version 1.
DT   25-APR-2018, entry version 67.
DE   RecName: Full=Defense against restriction protein B {ECO:0000303|PubMed:3029954};
DE            EC=3.6.4.- {ECO:0000305};
DE   AltName: Full=DarB {ECO:0000312|EMBL:AAQ14093.1};
GN   Name=darB {ECO:0000312|EMBL:AAQ14093.1};
OS   Escherichia phage P1 (Bacteriophage P1).
OC   Viruses; dsDNA viruses, no RNA stage; Caudovirales; Myoviridae;
OC   P1virus.
OX   NCBI_TaxID=10678 {ECO:0000312|Proteomes:UP000008091};
OH   NCBI_TaxID=543; Enterobacteriaceae.
RN   [1]
RP   NUCLEOTIDE SEQUENCE [GENOMIC DNA].
RC   STRAIN=Mod1902::IS5 c1.100 rev dmt {ECO:0000312|EMBL:AAQ14093.1}, and
RC   Mod749::IS5 c1.100 mutant {ECO:0000312|EMBL:AAQ13985.1};
RX   PubMed=15489417; DOI=10.1128/JB.186.21.7032-7068.2004;
RA   Lobocka M.B., Rose D.J., Plunkett G. III, Rusin M., Samojedny A.,
RA   Lehnherr H., Yarmolinsky M.B., Blattner F.R.;
RT   "Genome of bacteriophage P1.";
RL   J. Bacteriol. 186:7032-7068(2004).
RN   [2]
RP   FUNCTION, AND SUBCELLULAR LOCATION.
RX   PubMed=3029954; DOI=10.1016/0042-6822(87)90324-2;
RA   Iida S., Streiff M.B., Bickle T.A., Arber W.;
RT   "Two DNA antirestriction systems of bacteriophage P1, darA, and darB:
RT   characterization of darA- phages.";
RL   Virology 157:156-166(1987).
CC   -!- FUNCTION: Capsid internal protein that is ejected along with the
```

```
CC       viral DNA and prevents degradation of viral DNA by the host
CC       restriction-modification antiviral defense system.
CC       {ECO:0000269|PubMed:3029954}.
CC   -!- SUBCELLULAR LOCATION: Virion {ECO:0000269|PubMed:3029954}.
CC       Note=Internal capsid protein. {ECO:0000269|PubMed:3029954}.
CC   -!- SIMILARITY: Belongs to the helicase family. {ECO:0000305}.
DR   EMBL; AF234172; AAQ13985.1; -; Genomic_DNA.
DR   EMBL; AF234173; AAQ14093.1; -; Genomic_DNA.
DR   RefSeq; YP_006479.1; NC_005856.1.
DR   ProteinModelPortal; Q71TF8; -.
DR   GeneID; 2777481; -.
DR   KEGG; vg:2777481; -.
DR   Proteomes; UP000001577; Genome.
DR   Proteomes; UP000008091; Genome.
DR   GO; GO:0019012; C:virion; IEA:UniProtKB-SubCell.
DR   GO; GO:0005524; F:ATP binding; IEA:UniProtKB-KW.
DR   GO; GO:0003677; F:DNA binding; IEA:InterPro.
DR   GO; GO:0004386; F:helicase activity; IEA:UniProtKB-KW.
DR   GO; GO:0008170; F:N-methyltransferase activity; IEA:InterPro.
DR   GO; GO:0006306; P:DNA methylation; IEA:InterPro.
DR   GO; GO:0099018; P:restriction-modification system evasion by virus; IEA:UniProtKB-KW.
DR   Gene3D; 3.40.50.10810; -; 2.
DR   InterPro; IPR003356; DNA_methylase_A-5.
DR   InterPro; IPR014001; Helicase_ATP-bd.
DR   InterPro; IPR001650; Helicase_C.
DR   InterPro; IPR027417; P-loop_NTPase.
DR   InterPro; IPR029063; SAM-dependent_MTases.
DR   InterPro; IPR038718; SNF2-like_sf.
DR   InterPro; IPR000330; SNF2_N.
DR   Pfam; PF00271; Helicase_C; 1.
DR   Pfam; PF02384; N6_Mtase; 1.
DR   Pfam; PF00176; SNF2_N; 1.
DR   SMART; SM00487; DEXDc; 1.
DR   SMART; SM00490; HELICc; 1.
DR   SUPFAM; SSF52540; SSF52540; 5.
DR   SUPFAM; SSF53335; SSF53335; 1.
DR   PROSITE; PS51192; HELICASE_ATP_BIND_1; 1.
DR   PROSITE; PS51194; HELICASE_CTER; 1.
PE   3: Inferred from homology;
KW   ATP-binding; Coiled coil; Complete proteome; Helicase;
KW   Host-virus interaction; Hydrolase; Nucleotide-binding;
KW   Reference proteome; Restriction-modification system evasion by virus;
KW   Virion.
FT   CHAIN         1   2255       Defense against restriction protein B.
FT                                /FTId=PRO_0000433213.
FT   DOMAIN      841   1126       Helicase ATP-binding.
FT                                {ECO:0000255|PROSITE-ProRule:PRU00541}.
FT   DOMAIN     1383   1568       Helicase C-terminal.
FT                                {ECO:0000255|PROSITE-ProRule:PRU00542}.
FT   NP_BIND     854    861       ATP. {ECO:0000255|PROSITE-
FT                                ProRule:PRU00541}.
FT   COILED     1198   1234       {ECO:0000255}.
FT   COILED     1617   1654       {ECO:0000255}.
FT   MOTIF      1052   1055       DEAH box. {ECO:0000255|PROSITE-
FT                                ProRule:PRU00541}.
SQ   SEQUENCE   2255 AA;  251543 MW;  383BAA4D9D28341F CRC64;
     MNKLSMGVFR CSSVSEILKY IRAITSHRAP IKYGVEKVEG KSYDRLRREA NQKAIDLLNS
     LVDGATLTDE QRQILAGYTG EGGIGGSVSE YYTPKPIAEG VWEIMKLYGA DVGNTLEPSA
     GTGVFNETKP VGTVMTATEI SSVSGRINQL LHPEDSVQIS PFEQLAVSTP NDSFDHVVGN
     VPFGGRDNTR NIDKPYAEET DMGSYFMLRM LDKIKPGGFM CVIVPPSIVS GSNMKRLRLR
     LSRKAEFLGA HRLPTGTFDA NGTSTVVDVV LMRKHPAEMA EKIPLVHEST LESANVLWPT
     FISGKWFEKD GRRFVHGTQE KGFQGRIEVR ADGQIDNQAL KAKLIHCFES RIDWYLLDMA
     EPSPTADVVD EGEMRLINGV WQKYAGGRWI ESDAGKELKI DVASYGADSW EALQRNLTTT
     EGRLGMTFTQ MANVRDKYTT SISDDMVQLV DWINSQPEKY RERLYRGAMI GRMLIEYQDM
     KAAGHSAEQI EQQRLSLVSR LQAEIDRFGN PGRGPIAKLS GSGARAWFAF RGAIKLDGTI
     SDELTGKLVT HDSSASYDST SYQDTLRYLY SDLTRDPIQL DDFRLAFTGE LPASDDELLN
     LLASTPGIAV SPYGGIVPFA RATSGDINEI VAPKQEFLAT LTDGPVKNNV LNQLAAIEEK
     RIKTPAENIR FKLNSRWFDR SVILEFLQEN GYPDLRYVQS VQLEGDEMVS DTYHGGDGLF
     VGHRYGVVQR KDKETGEIRY EWDRKSGENA TGFPAQLEKY LNGARIGGKD SATANGYREQ
     MALLEDQFNK WIKTHDRYDE LVAKYNDVFN SNIPYEHSGD PLGLKGLSGK RQPFDYQNSE
     VRRLSEDGRG ILGFGTGLGK TTTALALEAF NYENGRSTRT AYVVPKSVLE NWYYEAKEFL
     SEEAFSNYLF VGLDVLMDGD QIRQVPVLDE NGKPVLGTDG TPVMRDALKL ADEATITARM
     NAIPHSNYRA VVFTKEQYAR IPLRDDTVDE HAQDMLYDFV AAGRVASAMD SDSHRKEAAR
     RRVLSEYSDT GTEKAEKYPY FEDMGFDSVI ADEGHNYRNS YKNGREASQL AYLPTSAVAQ
     SARDMAIKNA YLMKKNGGRG PVLLTATPVV NTPIDAYNML SHVLPKEYWQ KMGIYGPDDF
     VKFFGKTRLE TVQKISGEVE EKMALVGFEN LDALRGIFHR WTTLKTAEDV KDTVEIPELD
     EHQQDAPLTE EQLAAYEELR QQAEAAAKAN NGVTTSVNED GVIEHEKARP IFSIIRDMDR
     VCTDMDLYYR RITYRFLPEY ADAVQQLADS LPKQATSEDD DSDDSITQQS QYSLIDKGEF
     IQLQVPEAFE QEVNKRLARF GIDEQTVTHP VTPKYAKLIA TLKEFFPEGK QIIFTDEKTQ
     HQKLKRIICN ALNLEPSKVG ILNAQTVAEA GKTGKKLKAV KPPKELPDEP TDAQIAKYNE
     QMALYDAYIA QQNEMSLGGL EKIAADFQEG RTPIIICNKK AEVGINLHRG TTDIHHLTLP
     WTPASIAQRN GRGARVGSNR ASVRVHYYCG KGSFDEYRLK TLKRKAGWIS DILRSDKSEM
     ENADANDMIE MQMYTAKDDG ERLAMMQVQM DKAKAAQRAR QKEQATIDLQ NYIKAQHAAG
     EDVEVLTAEL ERSKAELEKT TAEVAKFKQA VMAKAADNAD WKARWGSVHH TDRMLLAQYR
     ASLKSAIQRK ANISQAISPY EKLLNRTQKA ATDIKRLRPL VEDAINKGIL DVDPDLVNHA
     SEFLVIGDRS WRVGQYYDCA GDIVRIKSLD FDSQRADVEI IFTFKGTKSG NWDVKTLDKQ
     VDVTPDEDAV MQKISGGVSI AGINDIISCD DFYRFQQRGM IKITDSYGVQ TTESGYSIDF
     VGTYTDPLKH AVYPDRRDGA LKSSIAKWVL GMMSEGNNRQ VRLAEVFLTE LFGSNYGDVI
     ASYGDTLSPE AIQEKIADAI ARMPEKTSQG ATRNGDSELE VTNAIFGTHE FRASDYEITT
     AQFGTIGIYS NKAEIKQAMD AASARIAAER EANLNHAVAA LTQSWVTAIR EAATTGKITP
     AIADVVNDGS KFMDAYKMDA VQLPSAYGQL SYRMTYNLVS MFSDLAILGL VDLNEVTPEL
     LSMRKNHVEI LQRINTVLAG RTDEEKQADA DRINLALGNI TEEEIAARNE KQEELSSIQG
```

```
DATSIAQSLG LNYRVSTADL KMMYAPKFAA GEVFGLQEAS GMKGVLFRAK DAIKTKFGAR
WLPAKAKNSD FPGNWWIIET KHNVADVLAV IQQYA
```

Mascot: http://www.matrixscience.com/

MATRIX SCIENCE MASCOT Search Results

Protein View: RDRP_SHVX

RNA replication protein OS=Shallot virus X GN=ORF1 PE=3 SV=1

**Database:**	SwissProt
**Score:**	83
**Expect:**	8.3e-05
**Monoisotopic mass (M_r):**	195947
**Calculated pI:**	8.56
**Taxonomy:**	Shallot virus X

Sequence similarity is available as an NCBI BLAST search of RDRP_SHVX against nr.

Search parameters

**Enzyme:**	Trypsin: cuts C-term side of KR unless next residue is P.
**Fixed modifications:**	Carbamidomethyl (C)
**Variable modifications:**	Acetyl (N-term), Oxidation (M)
**Mass values searched:**	19
**Mass values matched:**	15

Protein sequence coverage: 10%

Matched peptides shown in ***bold red***.

1 **MTAVQKLFDQ** **ISDPNTK**AGY SNACFEAAQR RPKKAMAIAP FSVTTPEALT
51 LERFGITTSP FATTSHTHAA DKIIENDCLT IIGHYLPKRE AVTLIQLKRS
101 KIHLLGRQPS QDNFQNYCHE PKDVLR**YGIT** **HPNSCPVVNT** **EYAVLADTLH**
151 **FMSPR**QLYHL FSRNPKLERL FATLVLPIEA QHRLPSLFPD VYRLEYYKDH
201 FAYMPGGHGG GAYVHSYGTL KWLDTAQVGP VDYTKSSITN PWPITDYLSI
251 EKIETK**AAHH** **IMFIQR**TRAQ VDWPLPPIWV YHASEYVKLP LIFYPPEANV
301 QKTYPHTLIK RMQLYCFSVK AVSLRDIFAK LRQVIETQEL VRYSMADLIR
351 LANYFLFITG MNQVSDYESP LLENLFGKMC ASIRMRLRTF FQNLLGKTSY
401 AALLTVTDVI PVHFTTQPKR REAVGELWFQ EPKWSVSTMT QPRKEHHRLQ
451 MTWTLLAWFH QLESSGSMSE PCNNSESTPQ RTATSQQKAA KLTTSQKHNR
501 R**TDQTTMNPQ** **YPPLMLTIAP** **MMPRHSLMK**K TIATPCRTLE EISDLDLDDF
551 DDLPNEASNE PPSANEQSPD NHAETTTRGV FPCECGTEIT VNSFGRAIEV
601 AGVNLTDHMK GR**LAAFYSR**D GQGYSYTGYS HKSQGWLEGL DKLIEACGEK
651 PTTYNQCLVQ K**YEQGSRIGF** **HSDEQAIYPK** GNKILTVNAA GSGTFGIKCA
701 KGETTLNLED GDYFQMPSGF QETHKHNVVA VTPRLSFTFR STVVNSQKKP
751 AEPEKLNQNN ACPKPSDPSN ASGKQHKKTH PAKGNEKSSS PNLEPLDAPT
801 VEILKLHGFT ALTPQHDGTC QIRPVYFNKD IHLRRKAVKT DMSPPARPFF
851 DLATSLHRGI YTHKIDNRR**A** **TAYMSDVK**NN LTGLVLPKLD RDLLSSWVAL
901 AETTTREVAV LAIHGAGGAG KSRALQELLR SSPELADSIN IVVPTINLAN
951 DWKAKLPQMD PR**RVMTFQK**A CERECKSVTI FDDYGKLPAG FVDAYLAIKV
1001 NVELAILTGD QRQSTHHQER ESQISSLQSN IAQFSKYADY YLNATHRQPR
1051 **RLANPIK**VHA ERQLGGAVLK ANIVPDLAMV LVPAFR**SQSL** **LTDLGR**HAMT
1101 YAGCQGLTLN HLTIILDKDT PLCSDEVLYT AFSRASESIT FVNTHSDNPA
1151 FLAKLDATPY LKTLISWVRE DEEAGADCPA TEPLVKDVPT KTHIPVANDK
1201 VQLEGKIEAM EDKDTRELWS GEEKTNLMQT QDPVVQLFPH QQAKDEALFK
1251 **ITIGERIR**MA TPEQNAKQLR HTLNAGDLLF EAYAQFMKVP KETQPFDKRL
1301 WTHCRQLALR TYLSKPTSNL QQGARQDPDF PDNAIALFNK SQWVKKLEKV
1351 GARFKAGQTI SAFKQEVVLL TTTMALYLRK KREQHQPDNV FIMCERTPEQ
1401 FNAFVMTKWD FDRPNYTSDY TQYDQSQDAA FLNFEIRKAR HLGVPEDVLS
1451 FYKFIKTHAK **TFLGNLAIMR** LSAEGPTFDA NTECNIAYDA LRFRLGDDVR
1501 **ASYAGDDLVR** **DK**ACEERAGW VYSESLFSLK AKPLVTNKPD FCGWRLTRHG
1551 IVKSPIQLYQ SLQLALRLGK IDEVKRSYAI DYLFAYRLGD KIYDIFDEDE
1601 LEKHQLVTRT LIKKGMQPPE SGNHLPIFHI TSDRLIRDPD AVKVQSYECD
1651 RILLKQPHII DDYIPAGTQP RNTEHPASAD RRDMTRACNL SAEKLAFGGN
1701 TINHLFRTSW EGRSPLSN

Unformatted sequence string: 1718 residues (for pasting into other applications).

Sort by ◉ residue number ○ increasing mass ○ decreasing mass
Show ◉ matched peptides only ○ predicted peptides also

Start - End	Observed	Mr(expt)	Mr(calc)	Delta	M	Peptide
1 - 17	1993.8740	1992.8667	1992.9826	-0.1159	1	-.MTAVQKLFDQISDPNTK.A + Acetyl (N-term); Oxidation (M)
127 - 155	3347.4663	3346.4590	3346.5751	-0.1161	0	R.YGITHPNSCPVVNTEYAVLADTLHFMSPR.Q + Acetyl (N-term); Oxidation (M)
257 - 266	1265.7153	1264.7080	1264.6499	0.0582	0	K.AAHHIMFIQR.T + Acetyl (N-term)
502 - 524	2663.0703	2662.0630	2662.2627	-0.1997	0	R.TDQTTMNPQYPPLMLTIAPMMPR.H + Oxidation (M)
502 - 524	2695.0667	2694.0594	2694.2525	-0.1931	0	R.TDQTTMNPQYPPLMLTIAPMMPR.H + 3 Oxidation (M)
502 - 529	3323.5437	3322.5364	3322.5528	-0.0164	1	R.TDQTTMNPQYPPLMLTIAPMMPRHSLMK.K + 5 Oxidation (M)
613 - 619	869.4517	868.4444	868.4443	0.0001	0	R.LAAFYSR.D + Acetyl (N-term)

Start – End	Observed	Mr(expt)	Mr(calc)	Delta	M	Peptide
662 – 680	2224.9736	2223.9663	2224.0549	-0.0885	1	K.YEQGSRIGFHSDEQAIYPK.G
870 – 878	1001.5526	1000.5453	1000.4535	0.0918	0	R.ATAYMSDVK.N + Oxidation (M)
963 – 969	925.5176	924.5103	924.4851	0.0252	1	R.RVMTFQK.A + Oxidation (M)
1051 – 1057	853.4665	852.4592	852.5181	-0.0589	1	R.RLANPIK.V + Acetyl (N-term)
1087 – 1096	1089.6101	1088.6028	1088.5826	0.0202	0	R.SQSLLTDLGR.H
1251 – 1258	957.5149	956.5076	956.5767	-0.0691	1	K.ITIGERIR.M
1461 – 1470	1177.6738	1176.6665	1176.6325	0.0340	0	K.TFLGNLAIMR.L + Acetyl (N-term)
1501 – 1512	1309.7448	1308.7375	1308.6310	0.1065	1	R.ASYAGDDLVRDK.A

No match to: 1133.6364, 1353.7568, 2691.0818, 2748.1172

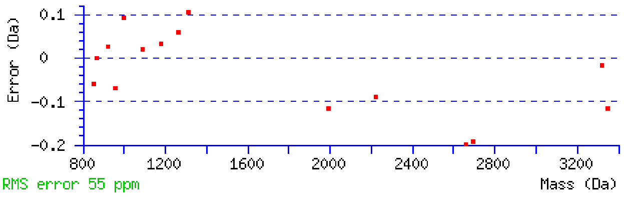

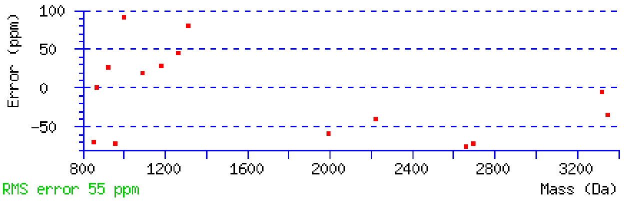


```
ID   RDRP_SHVX               Reviewed;         1718 AA.
AC   Q04575;
DT   01-JUN-1994, integrated into UniProtKB/Swiss-Prot.
DT   01-JUN-1994, sequence version 1.
DT   25-OCT-2017, entry version 89.
DE   RecName: Full=RNA replication protein;
DE   AltName: Full=194 kDa protein;
DE   Includes:
DE     RecName: Full=RNA-directed RNA polymerase;
DE              EC=2.7.7.48;
DE   Includes:
DE     RecName: Full=Helicase;
DE              EC=3.6.4.13;
GN   ORFNames=ORF1;
OS   Shallot virus X (ShVX).
OC   Viruses; ssRNA viruses; ssRNA positive-strand viruses, no DNA stage;
OC   Tymovirales; Alphaflexiviridae; Allexivirus.
OX   NCBI_TaxID=31770;
OH   NCBI_TaxID=28911; Allium cepa var. aggregatum (Shallot) (Allium ascalonicum).
RN   [1]
RP   NUCLEOTIDE SEQUENCE [GENOMIC RNA].
RX   PubMed=1339468; DOI=10.1099/0022-1317-73-10-2553;
RA   Kanyuka K.V., Vishnichenko V.K., Levay K.E., Kondrikov D.Y.,
RA   Ryabov E.V., Zavriev S.K.;
RT   "Nucleotide sequence of shallot virus X RNA reveals a 5'-proximal
RT   cistron closely related to those of potexviruses and a unique
RT   arrangement of the 3'-proximal cistrons.";
RL   J. Gen. Virol. 73:2553-2560(1992).
CC   -!- FUNCTION: RNA replication. The central part of this protein
CC       possibly functions as an ATP-binding helicase (Probable).
CC       {ECO:0000305}.
CC   -!- CATALYTIC ACTIVITY: Nucleoside triphosphate + RNA(n) = diphosphate
CC       + RNA(n+1). {ECO:0000255|PROSITE-ProRule:PRU00539}.
CC   -!- CATALYTIC ACTIVITY: ATP + H(2)O = ADP + phosphate.
CC   -!- SIMILARITY: Belongs to the potexvirus/carlavirus RNA replication
CC       protein family. {ECO:0000305}.
DR   EMBL; M97264; AAA47787.1; -; Genomic_RNA.
DR   PIR; JQ1734; JQ1734.
DR   RefSeq; NP_620648.1; NC_003795.1.
DR   ProteinModelPortal; Q04575; -.
DR   GeneID; 944368; -.
DR   KEGG; vg:944368; -.
DR   OrthoDB; VOG0900004V; -.
DR   Proteomes; UP000001663; Genome.
DR   GO; GO:0005524; F:ATP binding; IEA:UniProtKB-KW.
DR   GO; GO:0004386; F:helicase activity; IEA:UniProtKB-KW.
DR   GO; GO:0008174; F:mRNA methyltransferase activity; IEA:InterPro.
DR   GO; GO:0016491; F:oxidoreductase activity; IEA:InterPro.
DR   GO; GO:0003723; F:RNA binding; IEA:InterPro.
DR   GO; GO:0003968; F:RNA-directed 5'-3' RNA polymerase activity; IEA:UniProtKB-KW.
DR   GO; GO:0006396; P:RNA processing; IEA:InterPro.
DR   GO; GO:0006351; P:transcription, DNA-templated; IEA:InterPro.
DR   GO; GO:0039694; P:viral RNA genome replication; IEA:InterPro.
DR   Gene3D; 2.60.120.590; -; 1.
DR   InterPro; IPR027351; (+)RNA_virus_helicase_core_dom.
DR   InterPro; IPR027450; AlkB-like.
DR   InterPro; IPR037151; AlkB-like_sf.
DR   InterPro; IPR002588; Alphavirus-like_MT_dom.
DR   InterPro; IPR005123; Oxoglu/Fe-dep_dioxygenase.
DR   InterPro; IPR027417; P-loop_NTPase.
DR   InterPro; IPR007094; RNA-dir_pol_PSvirus.
DR   InterPro; IPR001788; Tymovirus_RNA-dep_RNA_pol.
DR   Pfam; PF13532; 2OG-FeII_Oxy_2; 1.
DR   Pfam; PF00978; RdRP_2; 1.
DR   Pfam; PF01443; Viral_helicase1; 1.
DR   Pfam; PF01660; Vmethyltransf; 1.
DR   SUPFAM; SSF52540; SSF52540; 1.
DR   PROSITE; PS51743; ALPHAVIRUS_MT; 1.
DR   PROSITE; PS51471; FE2OG_OXY; 1.
DR   PROSITE; PS51657; PSRV_HELICASE; 1.
DR   PROSITE; PS50507; RDRP_SSRNA_POS; 1.
PE   3: Inferred from homology;
```

```
KW   ATP-binding; Complete proteome; Helicase; Hydrolase;
KW   Multifunctional enzyme; Nucleotide-binding; Nucleotidyltransferase;
KW   Reference proteome; RNA-directed RNA polymerase; Transferase;
KW   Viral RNA replication.
FT   CHAIN         1   1718       RNA replication protein.
FT                                /FTId=PRO_0000222564.
FT   DOMAIN       59    223       Alphavirus-like MT. {ECO:0000255|PROSITE-
FT                                ProRule:PRU01079}.
FT   DOMAIN      653    743       Fe2OG dioxygenase. {ECO:0000255|PROSITE-
FT                                ProRule:PRU00805}.
FT   DOMAIN      889   1042       (+)RNA virus helicase ATP-binding.
FT   DOMAIN     1043   1176       (+)RNA virus helicase C-terminal.
FT   DOMAIN     1413   1520       RdRp catalytic. {ECO:0000255|PROSITE-
FT                                ProRule:PRU00539}.
FT   NP_BIND     915    922       ATP. {ECO:0000255}.
SQ   SEQUENCE   1718 AA;  194531 MW;  820FEAE1EB62415C CRC64;
     MTAVQKLFDQ ISDPNTKAGY SNACFEAAQR RPKKAMAIAP FSVTTPEALT LERFGITTSP
     FATTSHTHAA DKIIENDCLT IIGHYLPKRE AVTLIQLKRS KIHLLGRQPS QDNFQNYCHE
     PKDVLRYGIT HPNSCPVVNT EYAVLADTLH FMSPRQLYHL FSRNPKLERL FATLVLPIEA
     QHRLPSLFPD VYRLEYYKDH FAYMPGGHGG GAYVHSYGTL KWLDTAQVGP VDYTKSSITN
     PWPITDYLSI EKIETKAAHH IMFIQRTRAQ VDWPLPPIWV YHASEYVKLP LIFYPPEANV
     QKTYPHTLIK RMQLYCFSVK AVSLRDIFAK LRQVIETQEL VRYSMADLIR LANYFLFITG
     MNQVSDYESP LLENLFGKMC ASIRMRLRTF FQNLLGKTSY AALLTVTDVI PVHFTTQPKR
     REAVGELWFQ EPKWSVSTMT QPRKEHHRLQ MTWTLLAWFH QLESSGSMSE PCNNSESTPQ
     RTATSQQKAA KLTTSQKHNR RTDQTTMNPQ YPPLMLTIAP MMPRHSLMKK TIATPCRTLE
     EISDLDLDDF DDLPNEASNE PPSANEQSPD NHAETTTRGV FPCECGTEIT VNSFGRAIEV
     AGVNLTDHMK GRLAAFYSRD GQGYSYTGYS HKSQGWLEGL DKLIEACGEK PTTYNQCLVQ
     KYEQGSRIGF HSDEQAIYPK GNKILTVNAA GSGTFGIKCA KGETTLNLED GDYFQMPSGF
     QETHKHNVVA VTPRLSFTFR STVVNSQKKP AEPEKLNQNN ACPKPSDPSN ASGKQHKKTH
     PAKGNEKSSS PNLEPLDAPT VEILKLHGFT ALTPQHDGTC QIRPVYFNKD IHLRRKAVKT
     DMSPPARPFF DLATSLHRGI YTHKIDNRRA TAYMSDVKNN LTGLVLPKLD RDLLSSWVAL
     AETTTREVAV LAIHGAGGAG KSRALQELLR SSPELADSIN IVVPTINLAN DWKAKLPQMD
     PRRVMTFQKA CERECKSVTI FDDYGKLPAG FVDAYLAIKV NVELAILTGD QRQSTHHQER
     ESQISSLQSN IAQFSKYADY YLNATHRQPR RLANPIKVHA ERQLGGAVLK ANIVPDLAMV
     LVPAFRSQSL LTDLGRHAMT YAGCQGLTLN HLTIILDKDT PLCSDEVLYT AFSRASESIT
     FVNTHSDNPA FLAKLDATPY LKTLISWVRE DEEAGADCPA TEPLVKDVPT KTHIPVANDK
     VQLEGKIEAM EDKDTRELWS GEEKTNLMQT QDPVVQLFPH QQAKDEALFK ITIGERIRMA
     TPEQNAKQLR HTLNAGDLLF EAYAQFMKVP KETQPFDKRL WTHCRQLALR TYLSKPTSNL
     QQGARQDPDF PDNAIALFNK SQWVKKLEKV GARFKAGQTI SAFKQEVVLL TTTMALYLRK
     KREQHQPDNV FIMCERTPEQ FNAFVMTKWD FDRPNYTSDY TQYDQSQDAA FLNFEIRKAR
     HLGVPEDVLS FYKFIKTHAK TFLGNLAIMR LSAEGPTFDA NTECNIAYDA LRFRLGDDVR
     ASYAGDDLVR DKACEERAGW VYSESLFSLK AKPLVTNKPD FCGWRLTRHG IVKSPIQLYQ
     SLQLALRLGK IDEVKRSYAI DYLFAYRLGD KIYDIFDEDE LEKHQLVTRT LIKKGMQPPE
     SGNHLPIFHI TSDRLIRDPD AVKVQSYECD RILLKQPHII DDYIPAGTQP RNTEHPASAD
     RRDMTRACNL SAEKLAFGGN TINHLFRTSW EGRSPLSN
```

Mascot: http://www.matrixscience.com/

MATRIX SCIENCE MASCOT Search Results

Protein View: RDRP_SHVX

RNA replication protein OS=Shallot virus X GN=ORF1 PE=3 SV=1

**Database:**	SwissProt
**Score:**	115
**Expect:**	5.2e-08
**Monoisotopic mass (M_r):**	195947
**Calculated pI:**	8.56
**Taxonomy:**	Shallot virus X

Sequence similarity is available as an NCBI BLAST search of RDRP_SHVX against nr.

Search parameters

**Enzyme:**	Trypsin: cuts C-term side of KR unless next residue is P.
**Fixed modifications:**	Carbamidomethyl (C)
**Variable modifications:**	Acetyl (N-term), Oxidation (M)
**Mass values searched:**	27
**Mass values matched:**	20

Protein sequence coverage: 14%

Matched peptides shown in ***bold red***.

1	MTAVQK**LFDQ**	**ISDPNTK**AGY	SNACFEAAQR	RPKKAMAIAP	FSVTTPEALT
51	LERFGITTSP	FATTSHTHAA	DKIIENDCLT	IIGHYLPKRE	AVTLIQLKRS
101	KIHLLGRQPS	QDNFQNYCHE	PKDVLR**YGIT**	**HPNSCPVVNT**	**EYAVLADTLH**
151	**FMSPR**QLYHL	FSRNPKLERL	FATLVLPIEA	QHRLPSLFPD	VYRLEYYKDH
201	FAYMPGGHGG	GAYVHSYGTL	KWLDTAQVGP	VDYTKSSITN	PWPITDYLSI
251	EKIETK**AAHH**	**IMFIQR**TRAQ	VDWPLPPIWV	YHASEYVKLP	LIFYPPEANV
301	QK**TYPHTLIK**	RMQLYCFSVK	AVSLRDIFAK	LRQVIETQEL	VRYSMADLIR
351	LANYFLFITG	MNQVSDYESP	LLENLFGKMC	ASIRMRLRTF	FQNLLGKTSY
401	AALLTVTDVI	PVHFTTQPKR	REAVGELWFQ	EPKWSVSTMT	QPRKEHHRLQ
451	MTWTLLAWFH	QLESSGSMSE	PCNNSESTPQ	RTATSQQKAA	KLTTSQKHNR
501	**RTDQTTMNPQ**	**YPPLMLTIAP**	**MMPR**HSLMKK	TIATPCRTLE	EISDLDLDDF
551	DDLPNEASNE	PPSANEQSPD	NHAETTTRGV	FPCECGTEIT	VNSFGRAIEV
601	AGVNLTDHMK	GR**LAAFYSR**D	GQGYSYTGYS	HKSQGWLEGL	DKLIEACGEK
651	PTTYNQCLVQ	K**YEQGSRIGF**	**HSDEQAIYPK**	GNKILTVNAA	GSGTFGIKCA
701	KGETTLNLED	GDYFQMPSGF	QETHKHNVVA	VTPRLSFTFR	STVVNSQKKP
751	AEPEKLNQNN	ACPKPSDPSN	ASGKQHKKTH	PAKGNEKSSS	PNLEPLDAPT
801	VEILKLHGFT	ALTPQHDGTC	QIRPVYFNKD	IHLRRKAVKT	DMSPPARPFF
851	DLATSLHRGI	YTHKIDNRR**A**	**TAYMSDVK**NN	LTGLVLPKLD	RDLLSSWVAL
901	AETTTREVAV	LAIHGAGGAG	KSRALQELLR	SSPELADSIN	IVVPTINLAN
951	DWKAKLPQMD	PR**RVMTFQK**A	CERECKSVTI	FDDYGKLPAG	FVDAYLAIKV
1001	NVELAILTGD	QRQSTHHQER	ESQISSLQSN	IAQFSKYADY	YLNATHRQPR
1051	**RLANPIK**VHA	ERQLGGAVLK	ANIVPDLAMV	LVPAFR**SQSL**	**LTDLGR**HAMT
1101	YAGCQGLTLN	HLTIILDKDT	PLCSDEVLYT	AFSRASESIT	FVNTHSDNPA
1151	FLAKLDATPY	LKTLISWVRE	DEEAGADCPA	TEPLVKDVPT	KTHIPVANDK
1201	VQLEGKIEAM	EDKDTR**ELWS**	**GEEKTNLMQT**	**QDPVVQLFPH**	**QQAK**DEALFK
1251	**ITIGERIRMA**	**TPEQNAK**QLR	HTLNAGDLLF	EAYAQFMKVP	KETQPFDKRL
1301	WTHCRQLALR	TYLSKPTSNL	QQGARQDPDF	PDNAIALFNK	SQWVKKLEKV
1351	GARFKAGQTI	SAFKQEVVLL	TTTMALYLRK	KREQHQPDNV	FIMCERTPEQ
1401	FNAFVMTKWD	FDRPNYTSDY	TQYDQSQDAA	FLNFEIRKAR	HLGVPEDVLS
1451	FYKFIKTHAK	**TFLGNLAIMR**	LSAEGPTFDA	NTECNIAYDA	LRFRLGDDVR
1501	**ASYAGDDLVR**	**DK**ACEERAGW	VYSESLFSLK	AKPLVTNKPD	FCGWRLTRHG
1551	IVKSPIQLYQ	SLQLALRLGK	IDEVKRSYAI	DYLFAYRLGD	KIYDIFDEDE
1601	LEKHQLVTRT	LIKK**GMQPPE**	**SGNHLPIFHI**	**TSDR**LIRDPD	AVKVQSYECD
1651	RILLKQPHII	DDYIPAGTQP	RNTEHPASAD	RRDMTRACNL	SAEK**LAFGGN**
1701	**TINHLFRTSW**	**EGRSPLSN**			

Unformatted sequence string: 1718 residues (for pasting into other applications).

Sort by ◉ residue number ○ increasing mass ○ decreasing mass
Show ◉ matched peptides only ○ predicted peptides also

Start – End	Observed	Mr(expt)	Mr(calc)	Delta	M	Peptide
7 – 17	1277.7921	1276.7848	1276.6299	0.1549	0	K.LFDQISDPNTK.A
127 – 155	3347.5381	3346.5308	3346.5751	-0.0443	0	R.YGITHPNSCPVVNTEYAVLADTLHFMSPR.Q + Acetyl (N-term); Oxidation (M)
257 – 266	1265.7299	1264.7226	1264.6499	0.0728	0	K.AAHHIMFIQR.T + Acetyl (N-term)
303 – 310	1014.5367	1013.5294	1013.5546	-0.0251	0	K.TYPHTLIK.R + Acetyl (N-term)
501 – 524	2893.2424	2892.2351	2892.3642	-0.1291	1	R.RTDQTTMNPQYPPLMLTIAPMMPR.H + Acetyl (N-term); 3 Oxidation (M)
502 – 524	2663.1553	2662.1480	2662.2627	-0.1147	0	R.TDQTTMNPQYPPLMLTIAPMMPR.H + Oxidation (M)
613 – 619	869.4612	868.4540	868.4443	0.0097	0	R.LAAFYSR.D + Acetyl (N-term)

Start – End	Observed	Mr(expt)	Mr(calc)	Delta	M	Peptide
662 – 680	2225.0017	2223.9944	2224.0549	-0.0604	1	K.YEQGSRIGFHSDEQAIYPK.G
870 – 878	1001.5524	1000.5451	1000.4535	0.0916	0	R.ATAYMSDVK.N + Oxidation (M)
963 – 969	925.5306	924.5233	924.4851	0.0382	1	R.RVMTFQK.A + Oxidation (M)
1051 – 1057	853.4627	852.4554	852.5181	-0.0627	1	R.RLANPIK.V + Acetyl (N-term)
1087 – 1096	1089.6296	1088.6223	1088.5826	0.0397	0	R.SQSLLTDLGR.H
1217 – 1244	3323.6331	3322.6258	3322.6292	-0.0034	1	R.ELWSGEEKTNLMQTQDPVVQLFPHQQAK.D + Acetyl (N-term)
1251 – 1258	957.5207	956.5134	956.5767	-0.0633	1	K.ITIGERIR.M
1259 – 1267	1047.5728	1046.5655	1046.4702	0.0953	0	R.MATPEQNAK.Q + Acetyl (N-term); Oxidation (M)
1461 – 1470	1177.6792	1176.6719	1176.6325	0.0394	0	K.TFLGNLAIMR.L + Acetyl (N-term)
1501 – 1512	1309.7596	1308.7523	1308.6310	0.1213	1	R.ASYAGDDLVRDK.A
1615 – 1634	2232.9902	2231.9829	2232.0746	-0.0917	0	K.GMQPPESGNHLPIFHITSDR.L
1695 – 1713	2175.9656	2174.9583	2175.0974	-0.1390	1	K.LAFGGNTINHLFRTSWEGR.S
1708 – 1718	1233.7428	1232.7355	1232.5786	0.1570	1	R.TSWEGRSPLSN.-

No match to: 1133.6508, 1189.6995, 1637.8265, 2210.9878, 2807.1619, 2915.2930, 3794.8865

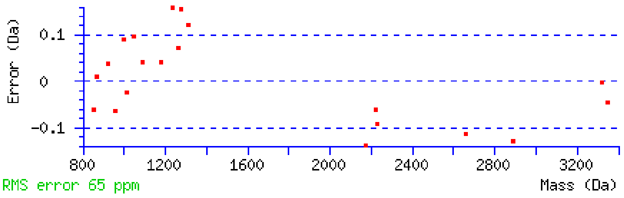

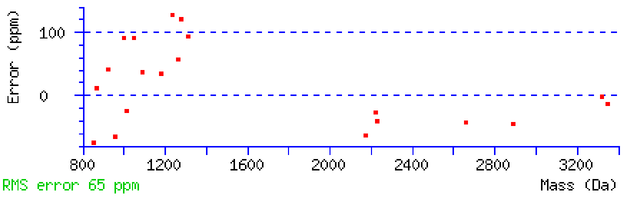


```
ID   RDRP_SHVX               Reviewed;        1718 AA.
AC   Q04575;
DT   01-JUN-1994, integrated into UniProtKB/Swiss-Prot.
DT   01-JUN-1994, sequence version 1.
DT   25-OCT-2017, entry version 89.
DE   RecName: Full=RNA replication protein;
DE   AltName: Full=194 kDa protein;
DE   Includes:
DE     RecName: Full=RNA-directed RNA polymerase;
DE              EC=2.7.7.48;
DE   Includes:
DE     RecName: Full=Helicase;
DE              EC=3.6.4.13;
GN   ORFNames=ORF1;
OS   Shallot virus X (ShVX).
OC   Viruses; ssRNA viruses; ssRNA positive-strand viruses, no DNA stage;
OC   Tymovirales; Alphaflexiviridae; Allexivirus.
OX   NCBI_TaxID=31770;
OH   NCBI_TaxID=28911; Allium cepa var. aggregatum (Shallot) (Allium ascalonicum).
RN   [1]
RP   NUCLEOTIDE SEQUENCE [GENOMIC RNA].
RX   PubMed=1339468; DOI=10.1099/0022-1317-73-10-2553;
RA   Kanyuka K.V., Vishnichenko V.K., Levay K.E., Kondrikov D.Y.,
RA   Ryabov E.V., Zavriev S.K.;
RT   "Nucleotide sequence of shallot virus X RNA reveals a 5'-proximal
RT   cistron closely related to those of potexviruses and a unique
RT   arrangement of the 3'-proximal cistrons.";
RL   J. Gen. Virol. 73:2553-2560(1992).
CC   -!- FUNCTION: RNA replication. The central part of this protein
CC       possibly functions as an ATP-binding helicase (Probable).
CC       {ECO:0000305}.
CC   -!- CATALYTIC ACTIVITY: Nucleoside triphosphate + RNA(n) = diphosphate
CC       + RNA(n+1). {ECO:0000255|PROSITE-ProRule:PRU00539}.
CC   -!- CATALYTIC ACTIVITY: ATP + H(2)O = ADP + phosphate.
CC   -!- SIMILARITY: Belongs to the potexvirus/carlavirus RNA replication
CC       protein family. {ECO:0000305}.
DR   EMBL; M97264; AAA47787.1; -; Genomic_RNA.
DR   PIR; JQ1734; JQ1734.
DR   RefSeq; NP_620648.1; NC_003795.1.
DR   ProteinModelPortal; Q04575; -.
DR   GeneID; 944368; -.
DR   KEGG; vg:944368; -.
DR   OrthoDB; VOG0900004V; -.
DR   Proteomes; UP000001663; Genome.
DR   GO; GO:0005524; F:ATP binding; IEA:UniProtKB-KW.
DR   GO; GO:0004386; F:helicase activity; IEA:UniProtKB-KW.
DR   GO; GO:0008174; F:mRNA methyltransferase activity; IEA:InterPro.
DR   GO; GO:0016491; F:oxidoreductase activity; IEA:InterPro.
DR   GO; GO:0003723; F:RNA binding; IEA:InterPro.
DR   GO; GO:0003968; F:RNA-directed 5'-3' RNA polymerase activity; IEA:UniProtKB-KW.
DR   GO; GO:0006396; P:RNA processing; IEA:InterPro.
DR   GO; GO:0006351; P:transcription, DNA-templated; IEA:InterPro.
DR   GO; GO:0039694; P:viral RNA genome replication; IEA:InterPro.
DR   Gene3D; 2.60.120.590; -; 1.
DR   InterPro; IPR027351; (+)RNA_virus_helicase_core_dom.
DR   InterPro; IPR027450; AlkB-like.
DR   InterPro; IPR037151; AlkB-like_sf.
DR   InterPro; IPR002588; Alphavirus-like_MT_dom.
DR   InterPro; IPR005123; Oxoglu/Fe-dep_dioxygenase.
DR   InterPro; IPR027417; P-loop_NTPase.
DR   InterPro; IPR007094; RNA-dir_pol_PSvirus.
DR   InterPro; IPR001788; Tymovirus_RNA-dep_RNA_pol.
DR   Pfam; PF13532; 2OG-FeII_Oxy_2; 1.
DR   Pfam; PF00978; RdRP_2; 1.
DR   Pfam; PF01443; Viral_helicase1; 1.
DR   Pfam; PF01660; Vmethyltransf; 1.
```

```
DR   SUPFAM; SSF52540; SSF52540; 1.
DR   PROSITE; PS51743; ALPHAVIRUS_MT; 1.
DR   PROSITE; PS51471; FE2OG_OXY; 1.
DR   PROSITE; PS51657; PSRV_HELICASE; 1.
DR   PROSITE; PS50507; RDRP_SSRNA_POS; 1.
PE   3: Inferred from homology;
KW   ATP-binding; Complete proteome; Helicase; Hydrolase;
KW   Multifunctional enzyme; Nucleotide-binding; Nucleotidyltransferase;
KW   Reference proteome; RNA-directed RNA polymerase; Transferase;
KW   Viral RNA replication.
FT   CHAIN         1   1718       RNA replication protein.
FT                                /FTId=PRO_0000222564.
FT   DOMAIN       59    223       Alphavirus-like MT. {ECO:0000255|PROSITE-
FT                                ProRule:PRU01079}.
FT   DOMAIN      653    743       Fe2OG dioxygenase. {ECO:0000255|PROSITE-
FT                                ProRule:PRU00805}.
FT   DOMAIN      889   1042       (+)RNA virus helicase ATP-binding.
FT   DOMAIN     1043   1176       (+)RNA virus helicase C-terminal.
FT   DOMAIN     1413   1520       RdRp catalytic. {ECO:0000255|PROSITE-
FT                                ProRule:PRU00539}.
FT   NP_BIND     915    922       ATP. {ECO:0000255}.
SQ   SEQUENCE   1718 AA;  194531 MW;  820FEAE1EB62415C CRC64;
     MTAVQKLFDQ ISDPNTKAGY SNACFEAAQR RPKKAMAIAP FSVTTPEALT LERFGITTSP
     FATTSHTHAA DKIIENDCLT IIGHYLPKRE AVTLIQLKRS KIHLLGRQPS QDNFQNYCHE
     PKDVLRYGIT HPNSCPVVNT EYAVLADTLH FMSPRQLYHL FSRNPKLERL FATLVLPIEA
     QHRLPSLFPD VYRLEYYKDH FAYMPGGHGG GAYVHSYGTL KWLDTAQVGP VDYTKSSITN
     PWPITDYLSI EKIETKAAHH IMFIQRTRAQ VDWPLPPIWV YHASEYVKLP LIFYPPEANV
     QKTYPHTLIK RMQLYCFSVK AVSLRDIFAK LRQVIETQEL VRYSMADLIR LANYFLFITG
     MNQVSDYESP LLENLFGKMC ASIRMRLRTF FQNLLGKTSY AALLTVTDVI PVHFTTQPKR
     REAVGELWFQ EPKWSVSTMT QPRKEHHRLQ MTWTLLAWFH QLESSGSMSE PCNNSESTPQ
     RTATSQQKAA KLTTSQKHNR RTDQTTMNPQ YPPLMLTIAP MMPRHSLMKK TIATPCRTLE
     EISDLDLDDF DDLPNEASNE PPSANEQSPD NHAETTTRGV FPCECGTEIT VNSFGRAIEV
     AGVNLTDHMK GRLAAFYSRD GQGYSYTGYS HKSQGWLEGL DKLIEACGEK PTTYNQCLVQ
     KYEQGSRIGF HSDEQAIYPK GNKILTVNAA GSGTFGIKCA KGETTLNLED GDYFQMPSGF
     QETHKHNVVA VTPRLSFTFR STVVNSQKKP AEPEKLNQNN ACPKPSDPSN ASGKQHKKTH
     PAKGNEKSSS PNLEPLDAPT VEILKLHGFT ALTPQHDGTC QIRPVYFNKD IHLRRKAVKT
     DMSPPARPFF DLATSLHRGI YTHKIDNRRA TAYMSDVKNN LTGLVLPKLD RDLLSSWVAL
     AETTTREVAV LAIHGAGGAG KSRALQELLR SSPELADSIN IVVPTINLAN DWKAKLPQMD
     PRRVMTFQKA CERECKSVTI FDDYGKLPAG FVDAYLAIKV NVELAILTGD QRQSTHHQER
     ESQISSLQSN IAQFSKYADY YLNATHRQPR RLANPIKVHA ERQLGGAVLK ANIVPDLAMV
     LVPAFRSQSL LTDLGRHAMT YAGCQGLTLN HLTIILDKDT PLCSDEVLYT AFSRASESIT
     FVNTHSDNPA FLAKLDATPY LKTLISWVRE DEEAGADCPA TEPLVKDVPT KTHIPVANDK
     VQLEGKIEAM EDKDTRELWS GEEKTNLMQT QDPVVQLFPH QQAKDEALFK ITIGERIRMA
     TPEQNAKQLR HTLNAGDLLF EAYAQFMKVP KETQPFDKRL WTHCRQLALR TYLSKPTSNL
     QQGARQDPDF PDNAIALFNK SQWVKKLEKV GARFKAGQTI SAFKQEVVLL TTTMALYLRK
     KREQHQPDNV FIMCERTPEQ FNAFVMTKWD FDRPNYTSDY TQYDQSQDAA FLNFEIRKAR
     HLGVPEDVLS FYKFIKTHAK TFLGNLAIMR LSAEGPTFDA NTECNIAYDA LRFRLGDDVR
     ASYAGDDLVR DKACEERAGW VYSESLFSLK AKPLVTNKPD FCGWRLTRHG IVKSPIQLYQ
     SLQLALRLGK IDEVKRSYAI DYLFAYRLGD KIYDIFDEDE LEKHQLVTRT LIKKGMQPPE
     SGNHLPIFHI TSDRLIRDPD AVKVQSYECD RILLKQPHII DDYIPAGTQP RNTEHPASAD
     RRDMTRACNL SAEKLAFGGN TINHLFRTSW EGRSPLSN
```

Mascot: http://www.matrixscience.com/

MATRIX SCIENCE MASCOT Search Results

Protein View: 017L_FRG3G

Uncharacterized protein 017L OS=Frog virus 3 (isolate Goorha) GN=FV3-017L PE=4 SV=1

**Database:**	SwissProt
**Score:**	59
**Expect:**	0.021
**Monoisotopic mass (M_r):**	54006
**Calculated pI:**	6.15
**Taxonomy:**	Frog virus 3 (isolate Goorha)

Sequence similarity is available as an NCBI BLAST search of 017L_FRG3G against nr.

Search parameters

**Enzyme:**	Trypsin: cuts C-term side of KR unless next residue is P.
**Fixed modifications:**	Carbamidomethyl (C)
**Variable modifications:**	Acetyl (N-term), Oxidation (M)
**Mass values searched:**	77
**Mass values matched:**	18

Protein sequence coverage: 31%

Matched peptides shown in ***bold red***.

```
  1 METMSDYSKE VSEALSALRG ELSALSAAIS NTVRAGSYSA PVAKDCKAGH
 51 CDSKAVLKSL SRSARDLDSA VEAVSSNCEW ASSGYGKQIA RALRDDAVRV
101 KREVESTRDA VDVVTPSCCV QGLAEEAGKL SEMAAVYRCM ATVFETADSH
151 GVREMLAKVD GLKQTMSGFK RLLGKTAEID GLSDSVIRLG RSIGEVLPAT
201 EGKAMRDLVK QCERLNGLVV DGSRKVEEQC SKLRDMASQS YVVADLASQY
251 DVLGGKAQEA LSASDALEQA AAVALRAKAA ADAVAKSLDS LDVKKLDRLL
301 EQASAVSGLL AKKNDLDAVV TSLAGLEALV AKKDELYKIC AAVNSVDKSK
351 LELLNVKPDR LKSLTEQTVV VSQMTTALAT FNEDKLDSVL GKYMQMHRFL
401 GMATQLKLMS DSLAEFQPAK MAQMAAAASQ LKDFLTDQTV SRLEKVSAAV
451 DATDVTKYAS AFSDGGMVSD MTKAYETVKA FAAVVNSLDS KKLKLVAECA
501 KK
```

Unformatted sequence string: 502 residues (for pasting into other applications).

Sort by ◉ residue number ○ increasing mass ○ decreasing mass
Show ◉ matched peptides only ○ predicted peptides also

Start – End	Observed	Mr(expt)	Mr(calc)	Delta	M	Peptide
1 – 9	1133.5773	1132.5700	1132.4416	0.1284	0	-.METMSDYSK.E + Acetyl (N-term)
1 – 9	1149.5775	1148.5702	1148.4366	0.1337	0	-.METMSDYSK.E + Acetyl (N-term); Oxidation (M)
1 – 19	2221.0857	2220.0784	2219.9926	0.0858	1	-.METMSDYSKEVSEALSALR.G + Acetyl (N-term); 2 Oxidation (M)
35 – 47	1353.7116	1352.7044	1352.6394	0.0649	1	R.AGSYSAPVAKDCK.A
45 – 54	1177.6083	1176.6010	1176.4652	0.1358	1	K.DCKAGHCDSK.A
88 – 94	869.4230	868.4158	868.5242	-0.1085	1	K.QIARALR.D + Acetyl (N-term)
92 – 99	957.4712	956.4639	956.5039	-0.0400	1	R.ALRDDAVR.V + Acetyl (N-term)
154 – 163	1145.6128	1144.6056	1144.6162	-0.0106	1	R.EMLAKVDGLK.Q + Acetyl (N-term)
154 – 163	1161.6192	1160.6119	1160.6111	0.0009	1	R.EMLAKVDGLK.Q + Acetyl (N-term); Oxidation (M)
164 – 171	970.5019	969.4946	969.4702	0.0244	1	K.QTMSGFKR.L + Oxidation (M)
207 – 214	1089.5534	1088.5461	1088.5284	0.0177	1	R.DLVKQCER.L + Acetyl (N-term)
215 – 224	1029.5405	1028.5332	1028.5615	-0.0283	0	R.LNGLVVDGSR.K
299 – 312	1441.7582	1440.7509	1440.8188	-0.0679	0	R.LLEQASAVSGLLAK.K + Acetyl (N-term)
333 – 338	837.4256	836.4183	836.4279	-0.0096	1	K.KDELYK.I + Acetyl (N-term)
393 – 398	897.4551	896.4478	896.3633	0.0845	0	K.YMQMHR.F + 2 Oxidation (M)
458 – 479	2374.1059	2373.0987	2373.0505	0.0482	1	K.YASAFSDGGMVSDMTKAYETVK.A + Oxidation (M)
480 – 491	1221.6299	1220.6226	1220.6401	-0.0174	0	K.AFAAVVNSLDSK.K
493 – 501	1073.5705	1072.5633	1072.5950	-0.0318	1	K.LKLVAECAK.K + Acetyl (N-term)

No match to: 749.4151, 765.3894, 781.3475, 787.4009, 793.4032, 825.3863, 853.4293, 855.0248, 877.0183, 892.9791, 898.4584, 913.4485, 925.4693, 928.4316, 941.4831, 947.5390, 985.5113, 1001.4968, 1013.5301, 1017.4951, 1057.5562, 1061.5179, 1101.5774, 1105.5479, 1117.5931, 1189.6405, 1205.6442, 1233.6604, 1237.6197, 1249.6758, 1293.6984, 1309.6821, 1337.7208, 1347.6965, 1381.7466, 1397.7386, 1409.6556, 1425.7736, 1603.8455, 1605.8078, 1672.8675, 1735.8426, 1790.8715, 1935.9080, 2233.0410, 2342.9279, 2398.9733, 2465.1078, 2662.2453, 2831.0738, 2877.2624, 2888.2567, 3015.4869, 3025.2830, 3047.4099, 3052.4633, 3114.2800, 3323.5142, 3635.7359

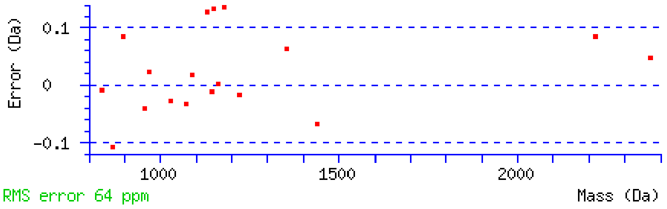

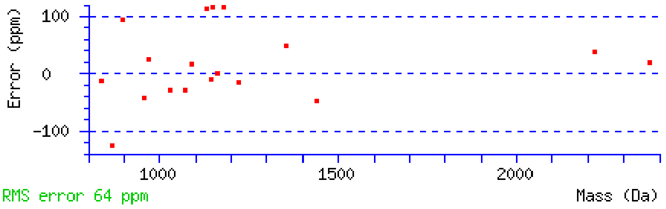


```
ID   017L_FRG3G              Reviewed;         502 AA.
AC   Q6GZV8;
DT   28-JUN-2011, integrated into UniProtKB/Swiss-Prot.
DT   19-JUL-2004, sequence version 1.
DT   27-SEP-2017, entry version 27.
DE   RecName: Full=Uncharacterized protein 017L;
GN   ORFNames=FV3-017L;
OS   Frog virus 3 (isolate Goorha) (FV-3).
OC   Viruses; dsDNA viruses, no RNA stage; Iridoviridae; Alphairidovirinae;
OC   Ranavirus.
OX   NCBI_TaxID=654924;
OH   NCBI_TaxID=8295; Ambystoma (mole salamanders).
OH   NCBI_TaxID=30343; Dryophytes versicolor (chameleon treefrog).
OH   NCBI_TaxID=8404; Lithobates pipiens (Northern leopard frog) (Rana pipiens).
OH   NCBI_TaxID=8316; Notophthalmus viridescens (Eastern newt) (Triturus viridescens).
OH   NCBI_TaxID=45438; Rana sylvatica (Wood frog).
RN   [1]
RP   NUCLEOTIDE SEQUENCE [LARGE SCALE GENOMIC DNA].
RX   PubMed=15165820; DOI=10.1016/j.virol.2004.02.019;
RA   Tan W.G., Barkman T.J., Gregory Chinchar V., Essani K.;
RT   "Comparative genomic analyses of frog virus 3, type species of the
RT   genus Ranavirus (family Iridoviridae).";
RL   Virology 323:70-84(2004).
DR   EMBL; AY548484; AAT09676.1; -; Genomic_DNA.
DR   RefSeq; YP_031595.1; NC_005946.1.
DR   SMR; Q6GZV8; -.
DR   GeneID; 2947737; -.
DR   KEGG; vg:2947737; -.
DR   OrthoDB; VOG0900003R; -.
DR   Proteomes; UP000008770; Genome.
PE   4: Predicted;
KW   Complete proteome; Reference proteome.
FT   CHAIN         1    502       Uncharacterized protein 017L.
FT                                /FTId=PRO_0000410557.
FT   COMPBIAS    425    428       Poly-Ala.
SQ   SEQUENCE   502 AA;  53469 MW;  A747EE6F952CBAD7 CRC64;
     METMSDYSKE VSEALSALRG ELSALSAAIS NTVRAGSYSA PVAKDCKAGH CDSKAVLKSL
     SRSARDLDSA VEAVSSNCEW ASSGYGKQIA RALRDDAVRV KREVESTRDA VDVVTPSCCV
     QGLAEEAGKL SEMAAVYRCM ATVFETADSH GVREMLAKVD GLKQTMSGFK RLLGKTAEID
     GLSDSVIRLG RSIGEVLPAT EGKAMRDLVK QCERLNGLVV DGSRKVEEQC SKLRDMASQS
     YVVADLASQY DVLGGKAQEA LSASDALEQA AAVALRAKAA ADAVAKSLDS LDVKKLDRLL
     EQASAVSGLL AKKNDLDAVV TSLAGLEALV AKKDELYKIC AAVNSVDKSK LELLNVKPDR
     LKSLTEQTVV VSQMTTALAT FNEDKLDSVL GKYMQMHRFL GMATQLKLMS DSLAEFQPAK
     MAQMAAAASQ LKDFLTDQTV SRLEKVSAAV DATDVTKYAS AFSDGGMVSD MTKAYETVKA
     FAAVVNSLDS KKLKLVAECA KK
```

Mascot: http://www.matrixscience.com/

MATRIX SCIENCE MASCOT Search Results

Protein View: VP3_LDCPR

Putative structural protein VP3 OS=Lymantria dispar cypovirus 1 (isolate Rao) GN=S3 PE=4 SV=1

**Database:**	SwissProt
**Score:**	137
**Expect:**	3.3e-10
**Monoisotopic mass (M_r):**	140252
**Calculated pI:**	5.35
**Taxonomy:**	Lymantria dispar cypovirus 1 isolate Rao

Sequence similarity is available as an NCBI BLAST search of VP3_LDCPR against nr.

Search parameters

**Enzyme:**	Trypsin: cuts C-term side of KR unless next residue is P.
**Fixed modifications:**	Carbamidomethyl (C)
**Variable modifications:**	Acetyl (N-term), Oxidation (M)
**Mass values searched:**	24
**Mass values matched:**	19

Protein sequence coverage: 18%

Matched peptides shown in ***bold red***.

1	**MEINRAEIR**R	EITRYAGLIE	QQTQINITDN	DQDILKTLIA	DYNLRMRRDA
51	LLGELARLDE	LRDISQIKGD	EYKLTIPLLP	IISTLNQHEF	EIIQANIETD
101	FIADNVTFIT	SFIPADLDLE	QTIQHVFFR**T**	**TATTPYFR**SF	NLVIAILDYD
151	EDKGDVKLDV	KITITRSNNG	VFNYNYTWAG	KDYERVSICY	NLISYLQQIN
201	GPRGRDDEAE	MPIYEIVRQN	NGSQPSYASG	EHLYIVSSHL	HVDEIVRDRE
251	HRDISVDVTE	LNLMFPIVRM	FDPVDLRDIR	IEDVTPGIEF	TINMEVSTYL
301	TELSGSHVDT	QR**TIMNHAEK**	**IVGNYTGQQW**	**NVQSNMLSEV**	**R**TQKLEEEDE
351	EARQRGDYTT	STLVQTMAQV	SDLFSSTILY	RYAEAELDNT	VGAFELLRPV
401	MSIPTEYIHD	GRIGPITNIS	ASASIVTSSN	NGVGEVRNIF	KPIGDQTINE
451	AHFANVYSND	EYAIYLRFSY	RQAPVQSETV	YLQQALPSMR	IVSPSSVSTT
501	VSTALIGGNT	IRINCPIRPH	R**EDNFVAGGV**	**QIPRQSTAVE**	**IHVQEILIGY**
551	**R**QATTFPIDT	EGR**LSLELMY**	**GLESR**SAVGN	TMSPVRFVTV	NDGEFFGLTC
601	PIDLTLSTIV	DPASYLSDGV	ILVTTAFEDL	RGYAWLATLG	GDWPRTYNSS
651	MGAFNIFTGG	DINLSTEYGS	EMTYTFKVEL	PINYMFNNMT	ISSHNVPRVP
701	VLGVTYASIY	QDSRTDLEAR	**RFLQTLVFRI**	**HGSWSAR**VPY	PPGNLPTRNT
751	ANQHQDIQQV	INDSIFQELD	RLSDELLDLE	NRLDHLERQF	EMFIQSQESE
801	WWEILLNVVI	DISIGYFSTF	AGDALKNAQR	AITKAVGYTR	R**VLMTVTKTM**
851	**R**NGTIFTRLL	GAKNLSGQAL	ASLETLVESA	LRSINMKKSR	FMRGAEPLYK
901	TNK**VAQHIDN**	**TEKMNMMMDF**	**SFANR**NNR**QN**	**ITADTLSKMH**	**TQNAHGTSDT**
951	**ILPAMR**VYYR	PLGFLDKRVG	DALHTGITRP	EALKKQLRSD	VANVGTRAPS
1001	HAFMTYTDVL	YEDAGSYIVS	KRYLGIGELN	KFGRTTSDKN	AGIGGVNIKY
1051	RVNKITADGK	YIIDRLDYTE	SGYTALDVDR	LYSSLFGK**QG**	**DGLSTEQKWM**
1101	**DISKGVDAK**I	ISADMVSEEF	LSSK**YTGQMI**	**DELINSPPQF**	**NYSLVYR**NCQ
1151	DFALDVLRVA	QGFSPSNKWD	VSTAARMQQR	RVISLMDDLM	GESETFARSG
1201	RASQLLLRQV	RESYVKARK**R**	**GDLQAVKALQ**	**LRFK**GFF	

Unformatted sequence string: 1237 residues (for pasting into other applications).

Sort by ◉ residue number ○ increasing mass ○ decreasing mass
Show ◉ matched peptides only ○ predicted peptides also

Start – End	Observed	Mr(expt)	Mr(calc)	Delta	M	Peptide
1 – 9	1189.7052	1188.6979	1188.5921	0.1059	1	-.MEINRAEIR.R + Acetyl (N-term); Oxidation (M)
130 – 138	1057.6345	1056.6272	1056.5240	0.1032	0	R.TTATTPYFR.S
313 – 320	1001.5765	1000.5692	1000.4648	0.1045	0	R.TIMNHAEK.I + Acetyl (N-term); Oxidation (M)
313 – 341	3347.5530	3346.5457	3346.6187	-0.0729	1	R.TIMNHAEKIVGNYTGQQWNVQSNMLSEVR.T
522 – 551	3338.6755	3337.6682	3337.7419	-0.0737	1	R.EDNFVAGGVQIPRQSTAVEIHVQEILIGYR.Q
564 – 575	1426.8344	1425.8271	1425.7173	0.1098	0	R.LSLELMYGLESR.S + Oxidation (M)
721 – 729	1221.7096	1220.7023	1220.7030	-0.0006	1	R.RFLQTLVFR.I + Acetyl (N-term)
730 – 737	913.5563	912.5490	912.4566	0.0924	0	R.IHGSWSAR.V
842 – 851	1179.6614	1178.6541	1178.6515	0.0026	1	R.VLMTVTKTMR.N
904 – 925	2678.1401	2677.1328	2677.1393	-0.0065	1	K.VAQHIDNTEKMNMMMDFSFANR.N + 3 Oxidation (M)
904 – 925	2720.1306	2719.1233	2719.1499	-0.0265	1	K.VAQHIDNTEKMNMMMDFSFANR.N + Acetyl (N-term); 3 Oxidation (M)
929 – 956	3052.5044	3051.4971	3051.4866	0.0105	1	R.QNITADTLSKMHTQNAHGTSDTILPAMR.V
1089 – 1104	1822.9117	1821.9044	1821.8567	0.0477	1	K.QGDGLSTEQKWMDISK.G
1099 – 1104	837.4761	836.4689	836.3738	0.0950	0	K.WMDISK.G + Acetyl (N-term); Oxidation (M)
1099 – 1109	1265.7496	1264.7423	1264.6122	0.1302	1	K.WMDISKGVDAK.I + Oxidation (M)

Start – End	Observed	Mr(expt)	Mr(calc)	Delta	M	Peptide
1125 – 1147	2748.1890	2747.1817	2747.3265	-0.1448	0	K.YTGQMIDELINSPPQFNYSLVYR.N
1220 – 1227	928.5372	927.5300	927.5138	0.0162	1	K.RGDLQAVK.A + Acetyl (N-term)
1221 – 1232	1353.7897	1352.7824	1352.7776	0.0049	1	R.GDLQAVKALQLR.F + Acetyl (N-term)
1228 – 1234	875.5357	874.5284	874.5388	-0.0104	1	K.ALQLRFK.G

No match to: 1861.9130, 2398.9067, 2691.1431, 2831.0728, 3300.1628

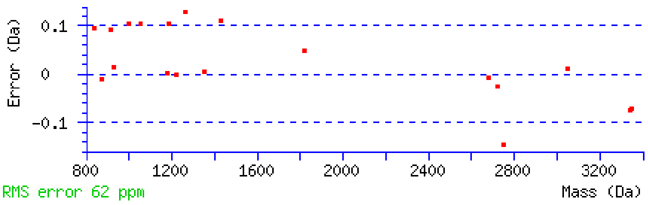

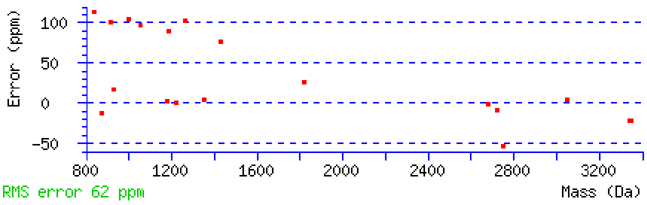


```
ID   VP3_LDCPR               Reviewed;        1237 AA.
AC   Q91ID9;
DT   11-JAN-2011, integrated into UniProtKB/Swiss-Prot.
DT   01-DEC-2001, sequence version 1.
DT   25-APR-2018, entry version 29.
DE   RecName: Full=Putative structural protein VP3;
GN   Name=S3;
OS   Lymantria dispar cypovirus 1 (isolate Rao) (LdCPV-1).
OC   Viruses; dsRNA viruses; Reoviridae; Spinareovirinae; Cypovirus.
OX   NCBI_TaxID=648169;
OH   NCBI_TaxID=13123; Lymantria dispar (Gypsy moth) (Porthetria dispar).
RN   [1]
RP   NUCLEOTIDE SEQUENCE [GENOMIC RNA].
RA   Rao S., Shapiro M., Lynn D., Hagiwara K., Blackmon B., Fang G.,
RA   Carner G.R.;
RT   "Identification of dsRNA electrophoretypes of two cypoviruses from a
RT   dual infection in gypsy moth, Lymantria dispar.";
RL   Submitted (JUN-2001) to the EMBL/GenBank/DDBJ databases.
CC   -!- SUBCELLULAR LOCATION: Virion {ECO:0000305}.
DR   EMBL; AF389464; AAK73522.1; -; Genomic_RNA.
DR   RefSeq; NP_149148.1; NC_003018.1.
DR   GeneID; 2598190; -.
DR   KEGG; vg:2598190; -.
DR   OrthoDB; VOG0900029C; -.
DR   Proteomes; UP000006712; Genome.
DR   GO; GO:0019028; C:viral capsid; IEA:UniProtKB-KW.
DR   PROSITE; PS51858; PPPDE; 1.
PE   4: Predicted;
KW   Capsid protein; Complete proteome; Reference proteome; Virion.
FT   CHAIN         1   1237       Putative structural protein VP3.
FT                                /FTId=PRO_0000403206.
FT   DOMAIN      963   1178       PPPDE. {ECO:0000255|PROSITE-
FT                                ProRule:PRU01205}.
FT   ACT_SITE   1001   1001       {ECO:0000255|PROSITE-ProRule:PRU01205}.
FT   ACT_SITE   1149   1149       {ECO:0000255|PROSITE-ProRule:PRU01205}.
SQ   SEQUENCE   1237 AA;  140111 MW;  618484A9F552461A CRC64;
     MEINRAEIRR EITRYAGLIE QQTQINITDN DQDILKTLIA DYNLRMRRDA LLGELARLDE
     LRDISQIKGD EYKLTIPLLP IISTLNQHEF EIIQANIETD FIADNVTFIT SFIPADLDLE
     QTIQHVFFRT TATTPYFRSF NLVIAILDYD EDKGDVKLDV KITITRSNNG VFNYNYTWAG
     KDYERVSICY NLISYLQQIN GPRGRDDEAE MPIYEIVRQN NGSQPSYASG EHLYIVSSHL
     HVDEIVRDRE HRDISVDVTE LNLMFPIVRM FDPVDLRDIR IEDVTPGIEF TINMEVSTYL
     TELSGSHVDT QRTIMNHAEK IVGNYTGQQW NVQSNMLSEV RTQKLEEEDE EARQRGDYTT
     STLVQTMAQV SDLFSSTILY RYAEAELDNT VGAFELLRPV MSIPTEYIHD GRIGPITNIS
     ASASIVTSSN NGVGEVRNIF KPIGDQTINE AHFANVYSND EYAIYLRFSY RQAPVQSETV
     YLQQALPSMR IVSPSSVSTT VSTALIGGNT IRINCPIRPH REDNFVAGGV QIPRQSTAVE
     IHVQEILIGY RQATTFPIDT EGRLSLELMY GLESRSAVGN TMSPVRFVTV NDGEFFGLTC
     PIDLTLSTIV DPASYLSDGV ILVTTAFEDL RGYAWLATLG GDWPRTYNSS MGAFNIFTGG
     DINLSTEYGS EMTYTFKVEL PINYMFNNMT ISSHNVPRVP VLGVTYASIY QDSRTDLEAR
     RFLQTLVFRI HGSWSARVPY PPGNLPTRNT ANQHQDIQQV INDSIFQELD RLSDELLDLE
     NRLDHLERQF EMFIQSQESE WWEILLNVVI DISIGYFSTF AGDALKNAQR AITKAVGYTR
     RVLMTVTKTM RNGTIFTRLL GAKNLSGQAL ASLETLVESA LRSINMKKSR FMRGAEPLYK
     TNKVAQHIDN TEKMNMMMDF SFANRNNRQN ITADTLSKMH TQNAHGTSDT ILPAMRVYYR
     PLGFLDKRVG DALHTGITRP EALKKQLRSD VANVGTRAPS HAFMTYTDVL YEDAGSYIVS
     KRYLGIGELN KFGRTTSDKN AGIGGVNIKY RVNKITADGK YIIDRLDYTE SGYTALDVDR
     LYSSLFGKQG DGLSTEQKWM DISKGVDAKI ISADMVSEEF LSSKYTGQMI DELINSPPQF
     NYSLVYRNCQ DFALDVLRVA QGFSPSNKWD VSTAARMQQR RVISLMDDLM GESETFARSG
     RASQLLLRQV RESYVKARKR GDLQAVKALQ LRFKGFF
```

Mascot: http://www.matrixscience.com/

MATRIX SCIENCE MASCOT Search Results

Protein View: PHD_BPP1

Antitoxin phd OS=Enterobacteria phage P1 GN=phd PE=1 SV=1

**Database:**	SwissProt
**Score:**	77
**Expect:**	0.00032
**Monoisotopic mass (M_r):**	8128
**Calculated pI:**	5.08
**Taxonomy:**	Escherichia virus P1

Sequence similarity is available as an NCBI BLAST search of PHD_BPP1 against nr.

Search parameters

**Enzyme:**	Trypsin: cuts C-term side of KR unless next residue is P.
**Fixed modifications:**	Carbamidomethyl (C)
**Variable modifications:**	Acetyl (N-term), Oxidation (M)
**Mass values searched:**	9
**Mass values matched:**	5

Protein sequence coverage: 35%

Matched peptides shown in ***bold red***.

```
 1 MQSINFRTAR GNLSEVLNNV EAGEEVEITR RGREPAVIVS KATFEAYKKA
51 ALDAEFASLF DTLDSTNKEL VNR
```

Unformatted sequence string: 73 residues (for pasting into other applications).

Sort by ◉ residue number ○ increasing mass ○ decreasing mass
Show ◉ matched peptides only ○ predicted peptides also

Start - End	Observed	Mr(expt)	Mr(calc)	Delta	M	Peptide
1 - 10	1265.7721	1264.7648	1264.6346	0.1302	1	-.MQSINFRTAR.G + Acetyl (N-term)
2 - 10	1134.5840	1133.5767	1133.5941	-0.0174	1	M.QSINFRTAR.G + Acetyl (N-term)
34 - 41	842.6375	841.6302	841.4909	0.1393	0	R.EPAVIVSK.A
42 - 48	871.3471	870.3398	870.4123	-0.0725	0	K.ATFEAYK.K + Acetyl (N-term)
42 - 49	957.5691	956.5618	956.4967	0.0651	1	K.ATFEAYKK.A

No match to: 2211.7358, 2225.7349, 2705.9341, 3052.5081

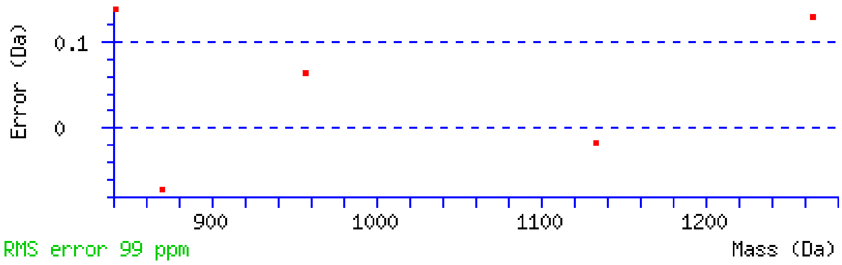

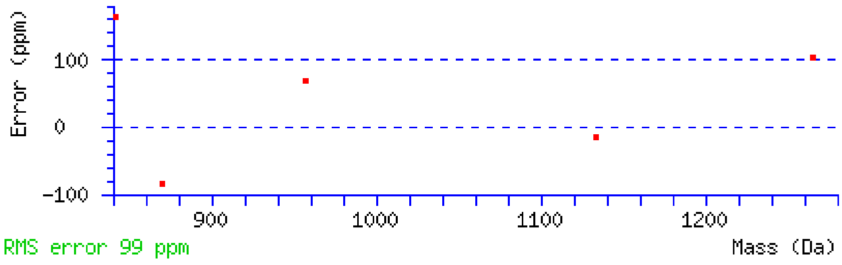

```
ID   PHD_BPP1                Reviewed;          73 AA.
AC   Q06253;
DT   01-FEB-1995, integrated into UniProtKB/Swiss-Prot.
DT   01-FEB-1995, sequence version 1.
DT   20-DEC-2017, entry version 90.
DE   RecName: Full=Antitoxin phd;
DE   AltName: Full=Addiction protein pdh;
DE   AltName: Full=Prevent host death protein;
GN   Name=phd;
OS   Escherichia phage P1 (Bacteriophage P1).
OC   Viruses; dsDNA viruses, no RNA stage; Caudovirales; Myoviridae;
OC   P1virus.
OX   NCBI_TaxID=10678;
OH   NCBI_TaxID=543; Enterobacteriaceae.
RN   [1]
RP   NUCLEOTIDE SEQUENCE.
RX   PubMed=8411153; DOI=10.1006/jmbi.1993.1521;
RA   Lehnherr H., Maguin E., Jafri S., Yarmolinsky M.B.;
RT   "Plasmid addiction genes of bacteriophage P1: doc, which causes cell
RT   death on curing of prophage, and phd, which prevents host death when
RT   prophage is retained.";
RL   J. Mol. Biol. 233:414-428(1993).
RN   [2]
RP   NUCLEOTIDE SEQUENCE [LARGE SCALE GENOMIC DNA].
RX   PubMed=15489417; DOI=10.1128/JB.186.21.7032-7068.2004;
RA   Lobocka M.B., Rose D.J., Plunkett G. III, Rusin M., Samojedny A.,
RA   Lehnherr H., Yarmolinsky M.B., Blattner F.R.;
RT   "Genome of bacteriophage P1.";
RL   J. Bacteriol. 186:7032-7068(2004).
RN   [3]
RP   CLEAVAGE BY THE CLPXP PROTEASE.
RX   PubMed=7724551; DOI=10.1073/pnas.92.8.3274;
RA   Lehnherr H., Yarmolinsky M.B.;
RT   "Addiction protein Phd of plasmid prophage P1 is a substrate of the
RT   ClpXP serine protease of Escherichia coli.";
RL   Proc. Natl. Acad. Sci. U.S.A. 92:3274-3277(1995).
RN   [4]
RP   FUNCTION AS A TRANSCRIPTION REGULATOR, DNA-BINDING, AND SUBUNIT.
RX   PubMed=9829946;
RA   Magnuson R., Yarmolinsky M.B.;
RT   "Corepression of the P1 addiction operon by Phd and Doc.";
RL   J. Bacteriol. 180:6342-6351(1998).
RN   [5]
RP   FUNCTION AS AN ANTITOXIN, AND SUBUNIT.
RX   PubMed=18398006; DOI=10.1073/pnas.0711949105;
RA   Liu M., Zhang Y., Inouye M., Woychik N.A.;
RT   "Bacterial addiction module toxin Doc inhibits translation elongation
RT   through its association with the 30S ribosomal subunit.";
RL   Proc. Natl. Acad. Sci. U.S.A. 105:5885-5890(2008).
```

```
RN   [6]
RP   FUNCTION AS ANTITOXIN, AND DOMAIN.
RX   PubMed=24141193; DOI=10.1038/nchembio.1364;
RA   Castro-Roa D., Garcia-Pino A., De Gieter S., van Nuland N.A.,
RA   Loris R., Zenkin N.;
RT   "The Fic protein Doc uses an inverted substrate to phosphorylate and
RT   inactivate EF-Tu.";
RL   Nat. Chem. Biol. 9:811-817(2013).
RN   [7]
RP   X-RAY CRYSTALLOGRAPHY (1.7 ANGSTROMS) OF 51-73 IN COMPLEX WITH TOXIN
RP   DOC.
RX   PubMed=18757857; DOI=10.1074/jbc.M805654200;
RA   Garcia-Pino A., Christensen-Dalsgaard M., Wyns L., Yarmolinsky M.,
RA   Magnuson R.D., Gerdes K., Loris R.;
RT   "Doc of prophage P1 is inhibited by its antitoxin partner Phd through
RT   fold complementation.";
RL   J. Biol. Chem. 283:30821-30827(2008).
RN   [8]
RP   X-RAY CRYSTALLOGRAPHY (2.4 ANGSTROMS), MODE OF TRANSCRIPTION
RP   REGULATION, AND MUTAGENESIS OF PHE-44; TYR-47 AND LYS-48.
RX   PubMed=20603017; DOI=10.1016/j.cell.2010.05.039;
RA   Garcia-Pino A., Balasubramanian S., Wyns L., Gazit E., De Greve H.,
RA   Magnuson R.D., Charlier D., van Nuland N.A., Loris R.;
RT   "Allostery and intrinsic disorder mediate transcription regulation by
RT   conditional cooperativity.";
RL   Cell 142:101-111(2010).
RN   [9]
RP   X-RAY CRYSTALLOGRAPHY (2.71 ANGSTROMS), AND SUBUNIT.
RX   PubMed=20696400; DOI=10.1016/j.str.2010.04.018;
RA   Arbing M.A., Handelman S.K., Kuzin A.P., Verdon G., Wang C., Su M.,
RA   Rothenbacher F.P., Abashidze M., Liu M., Hurley J.M., Xiao R.,
RA   Acton T., Inouye M., Montelione G.T., Woychik N.A., Hunt J.F.;
RT   "Crystal structures of Phd-Doc, HigA, and YeeU establish multiple
RT   evolutionary links between microbial growth-regulating toxin-antitoxin
RT   systems.";
RL   Structure 18:996-1010(2010).
CC   -!- FUNCTION: Antitoxin component of a type II toxin-antitoxin (TA)
CC       system (PubMed:18398006, PubMed:24141193, PubMed:18757857). A
CC       labile antitoxin that binds to cognate doc toxin and neutralizes
CC       its ability to phosphorylate host EF-Tu. Does not reverse
CC       phosphorylation. Bacteriophage P1 lysogenizes bacteria as a low-
CC       copy number plasmid; phd and doc proteins function in unison to
CC       stabilize plasmid number by inducing a lethal response to P1
CC       plasmid prophage loss (PubMed:8411153).
CC       {ECO:0000269|PubMed:18398006, ECO:0000269|PubMed:18757857,
CC       ECO:0000269|PubMed:24141193, ECO:0000269|PubMed:8411153}.
CC   -!- FUNCTION: Binds to its own promoter repressing its expression;
CC       toxin doc acts as a corepressor or derepressor depending on the
CC       ratio, repressing or inducing expression.
CC       {ECO:0000269|PubMed:20603017, ECO:0000269|PubMed:9829946}.
CC   -!- SUBUNIT: Homodimer. Interacts with cognate toxin doc, the exact
CC       ratio of doc:phd varies from 1:1 to 1:3. Interaction with doc
CC       prevents both kinase activity and dephosphorylation of EF-Tu.
CC       {ECO:0000269|PubMed:18398006, ECO:0000269|PubMed:18757857,
CC       ECO:0000269|PubMed:20696400, ECO:0000269|PubMed:9829946}.
CC   -!- INTERACTION:
CC       Q06259:doc; NbExp=4; IntAct=EBI-2908787, EBI-2908816;
CC   -!- PTM: Degraded by the ClpXP protease. {ECO:0000269|PubMed:7724551}.
CC   -!- MISCELLANEOUS: The concentration of phd in P1 lysogens is far
CC       greater than that of the toxin it antagonizes. Such an excess may
CC       assure the well-being of carriers of the addicting plasmid.
CC       {ECO:0000305|PubMed:8411153}.
CC   -!- SIMILARITY: Belongs to the phD/YefM antitoxin family.
CC       {ECO:0000305}.
DR   EMBL; M95666; AAA16932.1; -; Unassigned_DNA.
DR   EMBL; AF234172; AAQ14074.1; -; Genomic_DNA.
DR   PIR; S40015; S40015.
DR   RefSeq; YP_006570.1; NC_005856.1.
DR   PDB; 3DD7; X-ray; 1.70 A; B/D=51-73.
DR   PDB; 3HRY; X-ray; 2.25 A; A/B/C=1-73.
DR   PDB; 3HS2; X-ray; 2.20 A; A/B/C/D/E/F/G/H=1-58.
DR   PDB; 3K33; X-ray; 2.40 A; B/C/D=1-73.
```

```
DR   PDB; 3KH2; X-ray; 2.71 A; E/F/G/H=1-73.
DR   PDB; 4ZLX; X-ray; 2.31 A; A/B=1-45.
DR   PDB; 4ZM0; X-ray; 3.17 A; A/B/C/D=1-73.
DR   PDB; 4ZM2; X-ray; 3.88 A; A/B/C/D=1-73.
DR   PDBsum; 3DD7; -.
DR   PDBsum; 3HRY; -.
DR   PDBsum; 3HS2; -.
DR   PDBsum; 3K33; -.
DR   PDBsum; 3KH2; -.
DR   PDBsum; 4ZLX; -.
DR   PDBsum; 4ZM0; -.
DR   PDBsum; 4ZM2; -.
DR   DisProt; DP00288; -.
DR   ProteinModelPortal; Q06253; -.
DR   SMR; Q06253; -.
DR   DIP; DIP-62083N; -.
DR   IntAct; Q06253; 1.
DR   GeneID; 2777473; -.
DR   KEGG; vg:2777473; -.
DR   KO; K19165; -.
DR   EvolutionaryTrace; Q06253; -.
DR   Proteomes; UP000008091; Genome.
DR   GO; GO:0032993; C:protein-DNA complex; IDA:CAFA.
DR   GO; GO:0042803; F:protein homodimerization activity; IDA:CAFA.
DR   GO; GO:0043565; F:sequence-specific DNA binding; IDA:CAFA.
DR   GO; GO:0006355; P:regulation of transcription, DNA-templated; IEA:UniProtKB-KW.
DR   GO; GO:0006351; P:transcription, DNA-templated; IEA:UniProtKB-KW.
DR   InterPro; IPR006442; Antitoxin_Phd/YefM.
DR   InterPro; IPR036165; YefM-like_sf.
DR   Pfam; PF02604; PhdYeFM_antitox; 1.
DR   SUPFAM; SSF143120; SSF143120; 1.
PE   1: Evidence at protein level;
KW   3D-structure; Complete proteome; Reference proteome; Repressor;
KW   Toxin-antitoxin system; Transcription; Transcription regulation.
FT   CHAIN         1     73       Antitoxin phd.
FT                                /FTId=PRO_0000165279.
FT   REGION       50     73       Sufficient for antitoxin activity, its
FT                                presence prevents formation of a doc-EF-
FT                                Tu complex.
FT   MUTAGEN      44     44       F->A: Significantly decreases repressor
FT                                activity, binds DNA less well, inhibits
FT                                doc normally.
FT                                {ECO:0000269|PubMed:20603017}.
FT   MUTAGEN      47     47       Y->A: Decreases repressor activity, binds
FT                                DNA less well, inhibits doc normally.
FT                                {ECO:0000269|PubMed:20603017}.
FT   MUTAGEN      48     48       K->M: Decreases repressor activity, binds
FT                                DNA less well, inhibits doc normally.
FT                                {ECO:0000269|PubMed:20603017}.
FT   STRAND        2      5       {ECO:0000244|PDB:3HS2}.
FT   HELIX         6     11       {ECO:0000244|PDB:3HS2}.
FT   HELIX        13     21       {ECO:0000244|PDB:3HS2}.
FT   STRAND       26     29       {ECO:0000244|PDB:3HS2}.
FT   STRAND       31     33       {ECO:0000244|PDB:3HRY}.
FT   STRAND       36     40       {ECO:0000244|PDB:3HS2}.
FT   HELIX        41     49       {ECO:0000244|PDB:3HRY}.
FT   TURN         50     53       {ECO:0000244|PDB:3HRY}.
FT   HELIX        55     62       {ECO:0000244|PDB:3DD7}.
FT   HELIX        64     70       {ECO:0000244|PDB:3DD7}.
SQ   SEQUENCE   73 AA;  8133 MW;  5FDB9D3565440050 CRC64;
     MQSINFRTAR GNLSEVLNNV EAGEEVEITR RGREPAVIVS KATFEAYKKA ALDAEFASLF
     DTLDSTNKEL VNR
```

Mascot: http://www.matrixscience.com/

MATRIX SCIENCE MASCOT Search Results

Protein View: DPOL_BPKVM

DNA-directed DNA polymerase OS=Vibrio phage KVP40 (isolate Vibrio parahaemolyticus/Japan/Matsuzaki/1991) GN=43 PE=3 SV=1

**Database:**	SwissProt
**Score:**	93
**Expect:**	7.8e-06
**Monoisotopic mass (M_r):**	98921
**Calculated pI:**	5.59
**Taxonomy:**	Vibrio phage KVP40 Japan/Matsuzaki /1991

Sequence similarity is available as an NCBI BLAST search of DPOL_BPKVM against nr.

Search parameters

**Enzyme:**	Trypsin: cuts C-term side of KR unless next residue is P.
**Fixed modifications:**	Carbamidomethyl (C)
**Variable modifications:**	Acetyl (N-term), Oxidation (M)
**Mass values searched:**	26
**Mass values matched:**	17

Protein sequence coverage: 22%

Matched peptides shown in ***bold red***.

1 MTIYTSIER**I** **GSNLCERYID** **DDGFEHMR**KV KYEPTLFIHC NEETGYK**DIY**
51 **GRNCRPK**MFD TMGEASKYIK ETKQFNEVLG MDDFIVTYIS DVYKQREFDM
101 SRIRIANIDI ETPSVEFPEA AHAPVPITSI GHYDNIDDKF YVYGIPSNTE
151 WKRESSIVKP ELLEKTVYIR CATEKELLVK **YLQFWR**EKTP AIVTGWNIES
201 FDMPYIVNRY K**NLFGEK**VMN SLSPWGKVQV STTVNDYGQE ICKVNILGVS
251 ELDYLQLYKK FTYVTRPSYR **LDYIGEVELD** **EK**KVEFEQAN YLEFYEQDYQ
301 NFIDYQIQDV NLVKRLDEKL QLMLLTISLA YYAGINYQTV LGTIKPWDAI
351 IFNSLKAEKK VVPMMRSHEG GR**FMGAFVKA** **PQVGYHR**GIG SFDLTSLYPS
401 IIRECNISPE TIVGQLDYEG SLEDRIDKIV EGLITFPADE LSNSANGMQY
451 RK**DVRGVIPV** **EIEK**VFFQRK ANKKK**AFEYE** **QQAIDIQKQM** **DEQGETPELR**
501 AAYEEAKHQA K**IYDVQQMAR** **K**ILINSLYGA LGNEYFRFYD LRNAEAVTAY
551 GQLAIKWVAR **DVNIWLNK**VC KTTDKDYVIY GDTDSIYVNF DPLLELTGIN
601 KLEGDDYTDK FAKVCETVET K**VINPSYEAL** **HKYMNTYER**Q MFMDREVLAR
651 TGFFIAKKR**Y** **ALDVQDNEGI** **R**KPKLKIMGI ETQRSSTPPL CQKGLKEAIR
701 LILQEGEAKL QEFVKGYEKE FKAAPYQEVS FVSSANNMNK YSDDKGNPGK
751 GCPGHVKGAL YYNK**LAEEHG** **FDK**INEGDKI AVVFLTR**NKH** **GIDRIAYPSG**
801 **GKLPEAISYL** **IDHVDYNR**LY EDKFIKPLSA ISEAIKFDYK KTITLESFFG

Unformatted sequence string: 850 residues (for pasting into other applications).

Sort by ◉ residue number ○ increasing mass ○ decreasing mass
Show ◉ matched peptides only ○ predicted peptides also

Start – End	Observed	Mr(expt)	Mr(calc)	Delta	M	Peptide
10 – 28	2342.9771	2341.9698	2342.0056	-0.0357	1	R.IGSNLCERYIDDDGFEHMR.K + Oxidation (M)
48 – 57	1320.7290	1319.7217	1319.6404	0.0813	1	K.DIYGRNCRPK.M + Acetyl (N-term)
181 – 186	912.6708	911.6635	911.4654	0.1981	0	K.YLQFWR.E
212 – 217	749.4519	748.4446	748.3755	0.0691	0	K.NLFGEK.V + Acetyl (N-term)
271 – 282	1422.7952	1421.7879	1421.6926	0.0954	0	R.LDYIGEVELDEK.K
373 – 387	1707.8656	1706.8583	1706.8715	-0.0132	1	R.FMGAFVKAPQVGYHR.G
373 – 387	1765.8289	1764.8216	1764.8770	-0.0554	1	R.FMGAFVKAPQVGYHR.G + Acetyl (N-term); Oxidation (M)
453 – 464	1353.8245	1352.8172	1352.7664	0.0509	1	K.DVRGVIPVEIEK.V
476 – 500	3038.3560	3037.3487	3037.3974	-0.0487	1	K.AFEYEQQAIDIQKQMDEQGETPELR.A + Acetyl (N-term)
512 – 521	1293.8473	1292.8400	1292.6547	0.1853	1	K.IYDVQQMARK.I + Acetyl (N-term)
512 – 521	1309.8186	1308.8113	1308.6496	0.1617	1	K.IYDVQQMARK.I + Acetyl (N-term); Oxidation (M)
561 – 568	1001.6204	1000.6131	1000.5342	0.0790	0	R.DVNIWLNK.V
622 – 639	2244.2314	2243.2241	2243.0681	0.1561	1	K.VINPSYEALHKYMNTYER.Q + Oxidation (M)
660 – 671	1434.8562	1433.8489	1433.6787	0.1703	0	R.YALDVQDNEGIR.K + Acetyl (N-term)
765 – 773	1045.6526	1044.6453	1044.4876	0.1577	0	K.LAEEHGFDK.I
788 – 794	881.5192	880.5120	880.4515	0.0605	1	R.NKHGIDR.I + Acetyl (N-term)
795 – 818	2691.1899	2690.1826	2690.3704	-0.1878	1	R.IAYPSGGKLPEAISYLIDHVDYNR.L

No match to: 952.5543, 1161.7587, 1374.8462, 2166.1741, 2208.2063, 2287.0842, 2587.2947, 2748.2292, 3052.5535

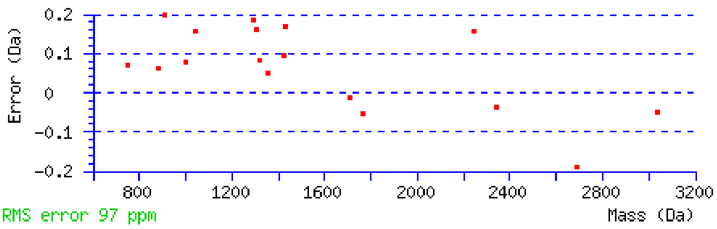

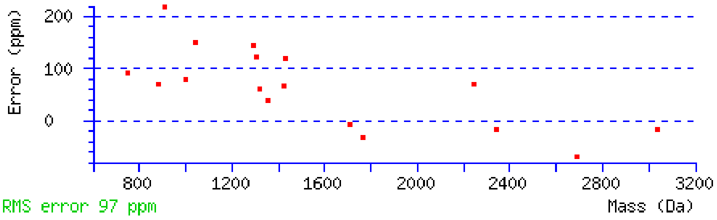

```
ID   DPOL_BPKVM              Reviewed;         850 AA.
AC   Q6WI70;
DT   04-FEB-2015, integrated into UniProtKB/Swiss-Prot.
DT   05-JUL-2004, sequence version 1.
DT   28-FEB-2018, entry version 79.
DE   RecName: Full=DNA-directed DNA polymerase {ECO:0000255|HAMAP-Rule:MF_04100};
DE            EC=2.7.7.7 {ECO:0000255|HAMAP-Rule:MF_04100, ECO:0000255|RuleBase:RU000442};
DE            EC=3.1.11.- {ECO:0000250|UniProtKB:P04415, ECO:0000255|HAMAP-Rule:MF_04100};
DE   AltName: Full=Gene product 43;
DE            Short=Gp43;
GN   Name=43 {ECO:0000312|EMBL:AAQ64153.1};
GN   ORFNames=KVP40.0082 {ECO:0000312|EMBL:AAQ64153.1};
OS   Vibrio phage KVP40 (isolate Vibrio
OS   parahaemolyticus/Japan/Matsuzaki/1991) (KVP40) (Bacteriophage KVP40).
OC   Viruses; dsDNA viruses, no RNA stage; Caudovirales; Myoviridae;
OC   Tevenvirinae; Schizot4virus.
OX   NCBI_TaxID=1283340;
OH   NCBI_TaxID=670; Vibrio parahaemolyticus.
RN   [1]
RP   NUCLEOTIDE SEQUENCE [GENOMIC DNA].
RC   STRAIN=Isolate Vibrio parahaemolyticus/Japan/Matsuzaki/1991
RC   {ECO:0000312|Proteomes:UP000001785};
RX   PubMed=12923095; DOI=10.1128/JB.185.17.5220-5233.2003;
RA   Miller E.S., Heidelberg J.F., Eisen J.A., Nelson W.C., Durkin A.S.,
RA   Ciecko A., Feldblyum T.V., White O., Paulsen I.T., Nierman W.C.,
RA   Lee J., Szczypinski B., Fraser C.M.;
RT   "Complete genome sequence of the broad-host-range vibriophage KVP40:
RT   comparative genomics of a T4-related bacteriophage.";
RL   J. Bacteriol. 185:5220-5233(2003).
CC   -!- FUNCTION: Replicates the viral genomic DNA. This polymerase
CC       possesses two enzymatic activities: DNA synthesis (polymerase) and
CC       an exonucleolytic activity that degrades single-stranded DNA in
CC       the 3'- to 5'-direction for proofreading purpose.
CC       {ECO:0000255|HAMAP-Rule:MF_04100}.
CC   -!- CATALYTIC ACTIVITY: Deoxynucleoside triphosphate + DNA(n) =
CC       diphosphate + DNA(n+1). {ECO:0000255|HAMAP-Rule:MF_04100}.
CC   -!- COFACTOR:
CC       Name=Mg(2+); Xref=ChEBI:CHEBI:18420;
CC         Evidence={ECO:0000255|HAMAP-Rule:MF_04100};
CC   -!- SUBUNIT: Part of the replicase complex that includes the DNA
CC       polymerase, the polymerase clamp, the clamp loader complex, the
CC       single-stranded DNA binding protein, and the primase/helicase.
CC       Interacts with the polymerase clamp; this interaction constitutes
CC       the polymerase holoenzyme. {ECO:0000255|HAMAP-Rule:MF_04100}.
CC   -!- DOMAIN: The N-terminus contains the 3'-5' exonuclease activity.
CC       The C-terminus contains the polymerase activity and is involved in
CC       binding to the polymerase clamp protein. A beta hairpin structure
CC       is necessary for the proofreading function of the polymerase.
CC       {ECO:0000255|HAMAP-Rule:MF_04100}.
CC   -!- SIMILARITY: Belongs to the DNA polymerase type-B family.
CC       {ECO:0000255|HAMAP-Rule:MF_04100}.
DR   EMBL; AY283928; AAQ64153.1; -; Genomic_DNA.
DR   RefSeq; NP_899330.1; NC_005083.2.
DR   ProteinModelPortal; Q6WI70; -.
DR   SMR; Q6WI70; -.
DR   GeneID; 2545927; -.
DR   KEGG; vg:2545927; -.
DR   KO; K18942; -.
DR   OrthoDB; VOG0900001M; -.
DR   Proteomes; UP000001785; Genome.
DR   GO; GO:0008408; F:3'-5' exonuclease activity; IEA:InterPro.
DR   GO; GO:0003677; F:DNA binding; IEA:UniProtKB-KW.
```

```
DR   GO; GO:0003887; F:DNA-directed DNA polymerase activity; IEA:UniProtKB-KW.
DR   GO; GO:0046872; F:metal ion binding; IEA:UniProtKB-KW.
DR   GO; GO:0000166; F:nucleotide binding; IEA:InterPro.
DR   GO; GO:0006260; P:DNA replication; IEA:UniProtKB-KW.
DR   GO; GO:0039693; P:viral DNA genome replication; IEA:UniProtKB-KW.
DR   Gene3D; 3.30.420.10; -; 1.
DR   HAMAP; MF_04100; DPOL_T4; 1.
DR   InterPro; IPR006172; DNA-dir_DNA_pol_B.
DR   InterPro; IPR017964; DNA-dir_DNA_pol_B_CS.
DR   InterPro; IPR006133; DNA-dir_DNA_pol_B_exonuc.
DR   InterPro; IPR006134; DNA-dir_DNA_pol_B_multi_dom.
DR   InterPro; IPR034749; DPOL_T4.
DR   InterPro; IPR012337; RNaseH-like_sf.
DR   InterPro; IPR036397; RNaseH_sf.
DR   Pfam; PF00136; DNA_pol_B; 1.
DR   Pfam; PF03104; DNA_pol_B_exo1; 1.
DR   PRINTS; PR00106; DNAPOLB.
DR   SMART; SM00486; POLBc; 1.
DR   SUPFAM; SSF53098; SSF53098; 1.
DR   PROSITE; PS00116; DNA_POLYMERASE_B; 1.
PE   3: Inferred from homology;
KW   Complete proteome; DNA replication; DNA-binding;
KW   DNA-directed DNA polymerase; Exonuclease; Hydrolase; Magnesium;
KW   Metal-binding; Multifunctional enzyme; Nuclease;
KW   Nucleotidyltransferase; Reference proteome; Transferase;
KW   Viral DNA replication.
FT   CHAIN         1    850       DNA-directed DNA polymerase.
FT                                /FTId=PRO_0000431825.
FT   REGION       98    322       3'-5'exonuclease. {ECO:0000255|HAMAP-
FT                                Rule:MF_04100}.
FT   REGION      228    245       Beta hairpin. {ECO:0000255|HAMAP-
FT                                Rule:MF_04100}.
FT   REGION      362    850       Polymerase. {ECO:0000255|HAMAP-
FT                                Rule:MF_04100}.
FT   REGION      396    398       Substrate binding. {ECO:0000255|HAMAP-
FT                                Rule:MF_04100}.
FT   REGION      657    660       Binding of DNA in B-conformation.
FT                                {ECO:0000255|HAMAP-Rule:MF_04100}.
FT   REGION      845    850       Interaction with the polymerase clamp.
FT                                {ECO:0000255|HAMAP-Rule:MF_04100}.
FT   METAL       109    109       Magnesium 1; catalytic; for 3'-5'
FT                                exonuclease activity. {ECO:0000255|HAMAP-
FT                                Rule:MF_04100}.
FT   METAL       111    111       Magnesium 1; catalytic; for 3'-5'
FT                                exonuclease activity. {ECO:0000255|HAMAP-
FT                                Rule:MF_04100}.
FT   METAL       202    202       Magnesium 2; catalytic; for 3'-5'
FT                                exonuclease activity. {ECO:0000255|HAMAP-
FT                                Rule:MF_04100}.
FT   METAL       309    309       Magnesium 1; catalytic; for 3'-5'
FT                                exonuclease activity. {ECO:0000255|HAMAP-
FT                                Rule:MF_04100}.
FT   METAL       309    309       Magnesium 2; catalytic; for 3'-5'
FT                                exonuclease activity. {ECO:0000255|HAMAP-
FT                                Rule:MF_04100}.
FT   METAL       393    393       Magnesium 3; catalytic; for polymerase
FT                                activity. {ECO:0000255|HAMAP-
FT                                Rule:MF_04100}.
FT   METAL       393    393       Magnesium 4; catalytic; for polymerase
FT                                activity. {ECO:0000255|HAMAP-
FT                                Rule:MF_04100}.
FT   METAL       394    394       Magnesium 4; catalytic; via carbonyl
FT                                oxygen; for polymerase activity.
FT                                {ECO:0000255|HAMAP-Rule:MF_04100}.
FT   METAL       584    584       Magnesium 3; catalytic; for polymerase
FT                                activity. {ECO:0000255|HAMAP-
FT                                Rule:MF_04100}.
FT   METAL       584    584       Magnesium 4; catalytic; for polymerase
FT                                activity. {ECO:0000255|HAMAP-
FT                                Rule:MF_04100}.
FT   BINDING     469    469       Substrate. {ECO:0000255|HAMAP-
FT                                Rule:MF_04100}.
FT   BINDING     521    521       Substrate. {ECO:0000255|HAMAP-
FT                                Rule:MF_04100}.
FT   SITE        582    582       Optimization of metal coordination by the
FT                                polymerase active site.
FT                                {ECO:0000255|HAMAP-Rule:MF_04100}.
FT   SITE        658    658       Optimization of metal coordination by the
FT                                polymerase active site.
FT                                {ECO:0000255|HAMAP-Rule:MF_04100}.
FT   SITE        666    666       Essential for viral replication.
FT                                {ECO:0000255|HAMAP-Rule:MF_04100}.
SQ   SEQUENCE   850 AA;  98413 MW;  03A7599EF571E470 CRC64;
     MTIYTSIERI GSNLCERYID DDGFEHMRKV KYEPTLFIHC NEETGYKDIY GRNCRPKMFD
     TMGEASKYIK ETKQFNEVLG MDDFIVTYIS DVYKQREFDM SRIRIANIDI ETPSVEFPEA
     AHAPVPITSI GHYDNIDDKF YVYGIPSNTE WKRESSIVKP ELLEKTVYIR CATEKELLVK
```

```
YLQFWREKTP AIVTGWNIES FDMPYIVNRY KNLFGEKVMN SLSPWGKVQV STTVNDYGQE
ICKVNILGVS ELDYLQLYKK FTYVTRPSYR LDYIGEVELD EKKVEFEQAN YLEFYEQDYQ
NFIDYQIQDV NLVKRLDEKL QLMLLTISLA YYAGINYQTV LGTIKPWDAI IFNSLKAEKK
VVPMMRSHEG GRFMGAFVKA PQVGYHRGIG SFDLTSLYPS IIRECNISPE TIVGQLDYEG
SLEDRIDKIV EGLITFPADE LSNSANGMQY RKDVRGVIPV EIEKVFFQRK ANKKKAFEYE
QQAIDIQKQM DEQGETPELR AAYEEAKHQA KIYDVQQMAR KILINSLYGA LGNEYFRFYD
LRNAEAVTAY GQLAIKWVAR DVNIWLNKVC KTTDKDYVIY GDTDSIYVNF DPLLELTGIN
KLEGDDYTDK FAKVCETVET KVINPSYEAL HKYMNTYERQ MFMDREVLAR TGFFIAKKRY
ALDVQDNEGI RKPKLKIMGI ETQRSSTPPL CQKGLKEAIR LILQEGEAKL QEFVKGYEKE
FKAAPYQEVS FVSSANNMNK YSDDKGNPGK GCPGHVKGAL YYNKLAEEHG FDKINEGDKI
AVVFLTRNKH GIDRIAYPSG GKLPEAISYL IDHVDYNRLY EDKFIKPLSA ISEAIKFDYK
KTITLESFFG
```

Mascot: http://www.matrixscience.com/

MATRIX SCIENCE MASCOT Search Results

Protein View: VG29_BPMD2

Gene 29 protein OS=Mycobacterium phage D29 GN=29 PE=4 SV=1

**Database:**	SwissProt
**Score:**	72
**Expect:**	0.0011
**Monoisotopic mass (M_r):**	17028
**Calculated pI:**	5.61
**Taxonomy:**	Mycobacterium virus D29

Sequence similarity is available as an NCBI BLAST search of VG29_BPMD2 against nr.

Search parameters

**Enzyme:**	Trypsin: cuts C-term side of KR unless next residue is P.
**Fixed modifications:**	Carbamidomethyl (C)
**Variable modifications:**	Acetyl (N-term), Oxidation (M)
**Mass values searched:**	14
**Mass values matched:**	6

Protein sequence coverage: 41%

Matched peptides shown in ***bold red***.

```
  1 MIPSQETHNP NDPRQHVVWA LRNLPLIAGV GAITHPAYLA DWSEHLWKCG
 51 FRHVDWLREL ADEDGNIHVS QLPDQQIKFQ PAFRGQRHDM NNAARWAEKD
101 APDPEPVRIP DIRKLTDQEN RAMLAQYERD GWIKNDRPGP AMAEVVE
```

Unformatted sequence string: 147 residues (for pasting into other applications).

Sort by ◉ residue number ○ increasing mass ○ decreasing mass
Show ◉ matched peptides only ○ predicted peptides also

Start – End	Observed	Mr(expt)	Mr(calc)	Delta	M	Peptide
53 – 58	825.4445	824.4372	824.4293	0.0079	0	R.HVDWLR.E
53 – 78	3055.4990	3054.4917	3054.5159	-0.0242	1	R.HVDWLRELADEDGNIHVSQLPDQQIK.F
79 – 84	765.4356	764.4283	764.3970	0.0314	0	K.FQPAFR.G
88 – 95	986.5886	985.5813	985.4035	0.1778	0	R.HDMNNAAR.W + Acetyl (N-term); Oxidation (M)
114 – 121	1045.6342	1044.6269	1044.5200	0.1070	1	R.KLTDQENR.A + Acetyl (N-term)
122 – 134	1638.9562	1637.9489	1637.7871	0.1618	1	R.AMLAQYERDGWIK.N + Acetyl (N-term); Oxidation (M)

No match to: 1133.6824, 1221.7356, 2398.9775, 2663.1685, 2691.1846, 2748.2229, 3052.5325, 3323.7000

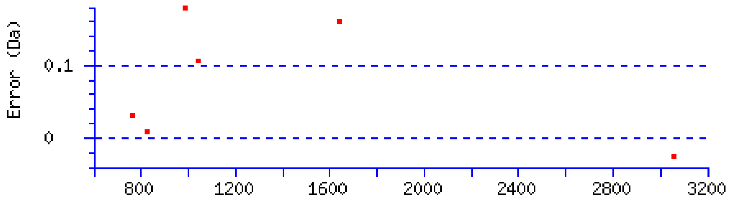

RMS error 95 ppm

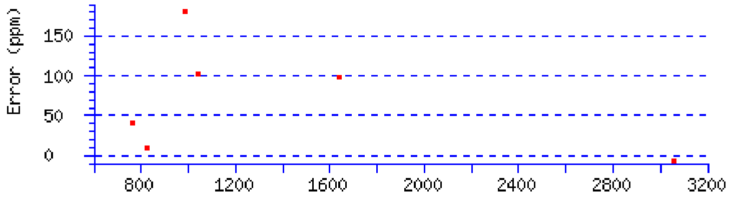

RMS error 95 ppm

```
ID   VG29_BPMD2              Reviewed;         147 AA.
AC   O64223;
DT   15-DEC-1998, integrated into UniProtKB/Swiss-Prot.
DT   01-AUG-1998, sequence version 1.
DT   15-FEB-2017, entry version 40.
DE   RecName: Full=Gene 29 protein;
```

```
DE   AltName: Full=Gp29;
GN   Name=29;
OS   Mycobacterium phage D29 (Mycobacteriophage D29).
OC   Viruses; dsDNA viruses, no RNA stage; Caudovirales; Siphoviridae;
OC   L5virus.
OX   NCBI_TaxID=28369;
OH   NCBI_TaxID=1763; Mycobacterium.
RN   [1]
RP   NUCLEOTIDE SEQUENCE [LARGE SCALE GENOMIC DNA].
RX   PubMed=9636706; DOI=10.1006/jmbi.1997.1610;
RA   Ford M.E., Sarkis G.J., Belanger A.E., Hendrix R.W., Hatfull G.F.;
RT   "Genome structure of mycobacteriophage D29: implications for phage
RT   evolution.";
RL   J. Mol. Biol. 279:143-164(1998).
DR   EMBL; AF022214; AAC18470.1; -; Genomic_DNA.
DR   PIR; C72803; C72803.
DR   RefSeq; NP_046845.1; NC_001900.1.
DR   GeneID; 1261602; -.
DR   KEGG; vg:1261602; -.
DR   OrthoDB; VOG0900010Z; -.
DR   Proteomes; UP000002131; Genome.
DR   InterPro; IPR021226; DUF2744.
DR   Pfam; PF10910; DUF2744; 1.
PE   4: Predicted;
KW   Complete proteome; Reference proteome.
FT   CHAIN         1    147       Gene 29 protein.
FT                                /FTId=PRO_0000164746.
SQ   SEQUENCE   147 AA;  16982 MW;  54B877CBEDA82C33 CRC64;
     MIPSQETHNP NDPRQHVVWA LRNLPLIAGV GAITHPAYLA DWSEHLWKCG FRHVDWLREL
     ADEDGNIHVS QLPDQQIKFQ PAFRGQRHDM NNAARWAEKD APDPEPVRIP DIRKLTDQEN
     RAMLAQYERD GWIKNDRPGP AMAEVVE
```

Mascot: http://www.matrixscience.com/
